# Antenatal prediction of small for gestational age at birth based on four birthweight standards using machine learning algorithms

**DOI:** 10.3389/frai.2025.1679979

**Published:** 2026-01-12

**Authors:** Qiu-Yan Yu, Ying Lin, Yu-Run Zhou, Xin-Jun Yang, Joris Hemelaar

**Affiliations:** 1 Nuffield Department of Population Health, University of Oxford, Oxford, United Kingdom; 2 School of Preventive Medicine, Wenzhou Medical University, Wenzhou, Zhejiang, China; 3 Wenzhou Women and Children Health Guidance Center, Wenzhou, Zhejiang, China

**Keywords:** artificial intelligence, birthweight standards, feature selection, machine learning, prediction models, small-for-gestational-age

## Abstract

**Background:**

Accurate antenatal prediction of SGA at birth is essential to improve development and delivery of preventative and therapeutic interventions. This study aimed to assess the performance of machine learning (ML) models to predict SGA at birth among Chinese pregnancies classified according to the Chinese birthweight standard and three international birthweight standards.

**Methods:**

We collected multimodal, longitudinal, antenatal surveillance data on 350,135 singleton pregnancies in Wenzhou City, China, between Jan 1, 2014 and Dec 31, 2016. For three pregnancy intervals we developed ML prediction models for newborns classified as SGA using the China, Intergrowth 21st, Fetal Medicine Foundation (FMF), and Gestation-related Optimal Weight (GROW) standards. We applied lasso regression to conduct feature selection, and CatBoost, XGBoost, LightBoost, Artificial Neural Networks, Random Forest, Stacked ensemble model, and logistic regression for predictive modeling in training data sets, with validation in testing data sets.

**Results:**

Among 22,603 singleton pregnancies with complete data, the rate of SGA using the China standard was 6.1%, compared to 4.3, 6.0, and 9.7% for the Intergrowth 21st, GROW, and FMF standards, respectively. This pattern was maintained in the imputed data set (*n* = 225,523), with corresponding SGA rates of 6.8, 4.8, 7.4, and 10.7%. Late pregnancy models (<37 weeks) had the best power to predict SGA, compared to middle (<26 weeks) and early pregnancy (<18 weeks) models. With the China standard, the logistic regression model in late pregnancy performed best with an area under the receiver operating characteristic curve (ROC-AUC) of 0.74. Logistic regression also performed better than ML algorithms with the Intergrowth-21st and GROW standards at each pregnancy interval, although differences were small. The Random Forest model with the FMF standard achieved superior performance at each pregnancy interval, reaching a ROC-AUC of 0.79 in late pregnancy. Notably, the middle pregnancy Random Forest model with the FMF standard already attained a ROC-AUC of 0.72 at 26 weeks’ gestation. Symphysis-fundal height, maternal abdominal circumference, maternal age, maternal height and weight, and parity were consistently identified as key predictors of SGA across the different standards.

**Conclusion:**

There are important differences in the classification of SGA at birth between national and international birthweight standards. Both machine learning models and traditional logistic regression demonstrated comparable predictive performance for SGA identification. These findings hold promise for guiding risk-stratified prenatal care and optimizing resource allocation in clinical settings.

## Introduction

Small-for-gestational-age (SGA) is defined as birthweight for gestational age below the 10th centile according to a birthweight chart (American College of Obstetricians and Gynecologists' Committee on Practice Bulletins—Obstetrics, 2021). SGA newborns are a major cause of global neonatal and child mortality and morbidity, especially in low- and middle-income countries (LMICs) (Lee et al., 2013). An estimated 23.3 million infants (19.3% of live births) per year are born SGA in LMICs, which contribute to 21.9% of neonatal deaths (Lee et al., 2017). The highest rates and numbers of SGA infants are born in Asia, and China has the fifth highest number of SGA newborns annually (Lee et al., 2017). Sustainable Development Goal 3 (SDG3) target 3.2 aims to reduce neonatal and child mortality to 12 and 25 per 1,000 live births, respectively, in all countries by 2030 (Liu et al., 2016). However, many LMICs are not on track to meet these targets, highlighting an urgent need to address the adverse perinatal outcomes that contribute to neonatal and child mortality (Sharrow et al., 2022; GBD 2019 Under-5 Mortality Collaborators, 2021).

Crucially, SGA classification depends on the birthweight charts used, which include reference charts, prescriptive standards, and customized growth charts (Capital Institute of Pediatrics and Coordinating Study Group of Nine Cities on the Physical Growth and Development of Children, 2020; Gardosi et al., 2018; Nicolaides et al., 2018; Villar et al., 2014). Many countries use charts derived from their own population. For example, the Chinese newborn chart is a population-based chart based on healthy pregnant women from nine cities across China (Capital Institute of Pediatrics and Coordinating Study Group of Nine Cities on the Physical Growth and Development of Children, 2020). The Intergrowth 21st birthweight standard is a prescriptive international population-based standard derived from multi-ethnic urban populations in eight countries and selected healthy, well-nourished women receiving adequate antenatal care and at low risk of fetal growth impairment (Villar et al., 2014). The Fetal Medicine Foundation (FMF) chart is based on fetal estimated weight and birthweight data from unselected singleton pregnancies at two UK hospitals, including pregnancies at risk of complications and preterm babies in utero (Nicolaides et al., 2018). Unlike these universal charts, the customized Gestation-related Optimal Weight (GROW) chart adjusts for maternal weight, height, parity, ethnicity or country of origin, and fetal sex (Gardosi et al., 2018). Each birthweight chart classifies different populations of newborn babies as SGA. To our knowledge, few studies have compared SGA classification among Chinese pregnancies according to different birthweight standards.

It is crucial to improve antenatal prediction of SGA to enable development and implementation of preventative and therapeutic interventions. The traditional approach to risk prediction has been logistic regression based on known risk factors. However, this approach has proven to have poor predictive power for SGA (Bai et al., 2022; Bai et al., 2022). Given this limitation, there is a pressing need for more sophisticated analytical approaches. The field of perinatal epidemiology is now leveraging artificial intelligence (AI) to harness complex datasets for public health impact. AI promises a paradigm shift by uncovering subtle, non-linear interactions within routine clinical data that elude conventional methods (Mennickent et al., 2023). Large-scale, multimodal, longitudinal electronic health records facilitate the use of AI for predicting the risk of clinical outcomes (Hunter and Holmes, 2023). To date, studies to predict SGA at birth using Machine Learning (ML) have had important limitations, including small sample sizes, highly selected patient groups, and design or analysis biases (Bai et al., 2022; Bai et al., 2022; Vicoveanu et al., 2022). Some popular ML methods, such as a Stacked ensemble model that combines predictions from multiple base models using a meta-model to achieve superior performance, have not been applied to SGA prediction (Naimi and Balzer, 2018), and the predictive performance of these methods compared to other ML methods, such as Random Forests and Catboost, is unknown (Cho et al., 2022; Choi et al., 2021). In addition, a review of perinatal outcome prediction found that many ML models failed to explain their decision-making process to enable clinicians to understand the importance of input features (Ramakrishnan et al., 2021).

The development of accurate antenatal models for predicting SGA at birth requires high-performing ML algorithms. However, the accuracy of any such model is fundamentally dependent on the birthweight standards used to define SGA. Each standard identifies a different neonatal subpopulation, leading to substantial variation in clinical management. For example, infants classified as SGA by a customized standard (e.g., GROW) but not by a population standard (e.g., Intergrowth-21st) may miss essential hypoglycemia or hypothermia monitoring, whereas misclassifying a constitutionally small infant as SGA may prompt unnecessary investigations and parental anxiety. Thus, the choice of standard directly shapes risk stratification, resource use, and quality of care.

Therefore, this study aims to compare six machine learning (ML) models and logistic regression in predicting SGA based on four birthweight standards—the Chinese national standard, Intergrowth-21st, FMF, and GROW—and to evaluate how standard selection influences prediction accuracy.

## Methods

### Study design

**Figure 1:**
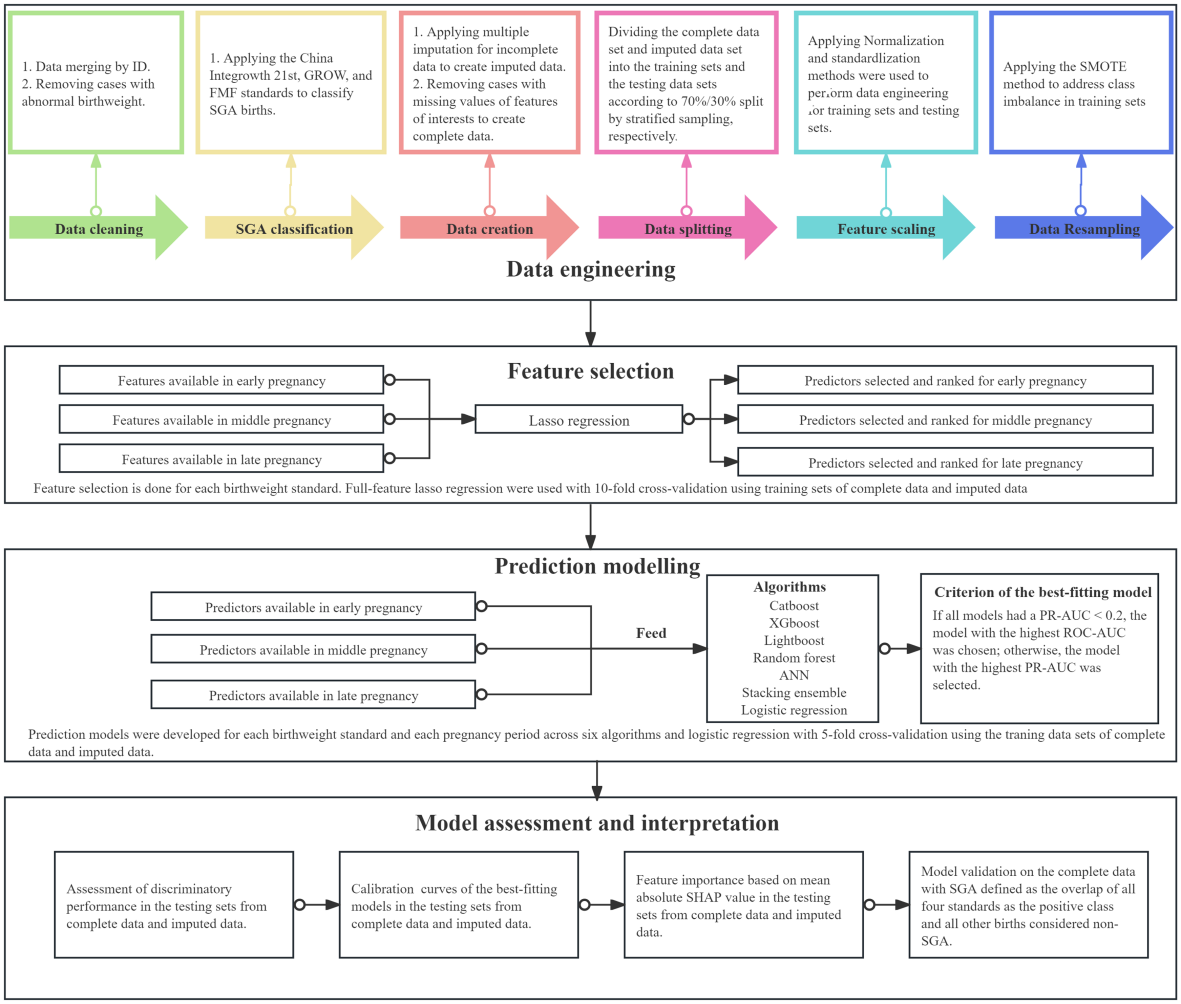
Study methodology. Steps to develop the machine learning models to predict small for gestational age are shown. Each step consists of several processes, as indicated. ANN, Artificial Neural Networks; FMF, Fetal Medicine Foundation; GROW, Gestation-Related Optimal Weight; SGA, small for gestational age (birthweight <10th centile for gestational age), SHAP, Shapley Additive Explanations; SMOTE, Synthetic Minority Over-sampling Technique; PR-AUC, Area Under the Precision-Recall Curve; ROC-AUC, Area Under the Receiver Operating Characteristic curve.

The Wenzhou maternal and child health information management platform covers 51 midwifery clinics and hospitals in Wenzhou City in Zhejiang Province, China, and was used to collect maternal and perinatal health records. We included all 350,135 singleton pregnancies registered from 1 January 2014 to 31 December 2016. Of these, 225,523 pregnancies were registered, had antenatal follow-up, and had delivery records (Supplementary Figure S1). The data analysis workflow, encompassing data engineering, feature selection, prediction modeling, and model performance and interpretation, is illustrated in Figure 1.

### Participant features

A prospective pregnancy health survey was conducted at registration at around 12 weeks’ gestation, collecting information regarding demographics, social, medical, obstetric and gynecological history, anthropometric measurements, and laboratory analyses. Gestational age at birth was determined at first-trimester ultrasound (standard practice). Birthweight was measured within 1 h of birth. Symphysis fundal height (SFH), maternal abdominal circumference (MAC), systolic blood pressure (SBP), diastolic blood pressure (DBP) and weight were measured at each antenatal care visit. Pregnancy was divided into three intervals which were determined based on a combination of clinical practice and the distribution of our dataset: early pregnancy (< 18 weeks’ gestation), middle of pregnancy (18 to 25^+6^ weeks), and late pregnancy (26 to 36^+6^ weeks) (Supplementary Table S1). Fifteen variables were created by dividing follow-up measurements into separate variables according to the three pregnancy intervals. If there were multiple visits during a given pregnancy interval, the average value of measurements was used for analysis. 43 features from registration and follow-up visits as well as four features from delivery data are shown in Supplementary Tables S2, S3. Additional variables were derived from the differences between pregnancy intervals (e.g., diffSBP12).

### Birthweight for gestational age standards

Singleton newborns with birthweight less than the 10th centile were classified as SGA based on four birthweight standards. SGA classification was according to newborn sex, except for the FMF standard (Nicolaides et al., 2018). Birthweight centiles for the China standard were based on the national reference, which was used for the primary endpoint of SGA classification in this study (Capital Institute of Pediatrics and Coordinating Study Group of Nine Cities on the Physical Growth and Development of Children, 2020). The GROW standard applied maternal height and weight at registration, parity, country of origin (China), fetal sex, and gestational age to calculate the birthweight centiles (www.gestation.net). Birthweight centiles for the Intergrowth 21st standard were calculated through its dedicated software (Villar et al., 2014). Therefore, four separate data sets were generated with SGA at birth classified according to each of the four birthweight standards, with the China Standard serving as the primary classification method for defining SGA and the other three standards as secondary classification methods.

### Data preprocessing

Data preprocessing for the cohort of 225,523 singleton pregnancies with registration, follow-up, and delivery records involved a staged process. Prior to imputation, variables with over 30% missing data were removed, reducing the feature set from 53 to 25. The MICE algorithm was then applied to these 25 variables to generate an imputed data set (*n* = 225,523), with the fifth iteration retained. In parallel, a complete data set (*n* = 22,603) was formed by excluding all pregnancy records with missing values from the 53 variables. The datasets were subsequently processed as follows: a 70%/30% stratified split was performed, using individual pregnancy records as the sampling unit. This approach was necessitated by the anonymized nature of the data, which precluded the identification of women with multiple pregnancies and ensured complete separation between training and testing sets. Following the split, all numeric features underwent normalization via the Yeo-Johnson method, followed by standardization (centering and scaling to achieve zero mean and unit variance). To address class imbalance, the Synthetic Minority Over-sampling Technique (SMOTE) was subsequently applied exclusively to the training sets.

### Feature selection

For each birthweight standard, Lasso regression was used to select important features for SGA prediction at three different time points: early (<18 weeks), middle (<26 weeks), and late pregnancy (<37 weeks). This analysis was performed on the training data sets of the imputed data and complete data using 10-fold cross-validation. Lasso regression was chosen for its advantage in handling multicollinearity among predictors. By applying an L1 penalty to the coefficients, lasso regression automatically identifies relevant predictors—shrinking the coefficients of less informative variables to zero—to yield a sparse subset of features.

### Design and development of prediction models

For each birthweight standard, we developed distinct prediction models for the early, middle, and late pregnancy intervals. The primary analysis was based on the complete data, while the imputed data were used in sensitivity analyses to evaluate the robustness of the models to missing data. In both analyses, the features were selected from variables available at each gestational interval using Lasso regression. The selected features were used to train the following algorithms: CatBoost, XGBoost, LightGBM, Random Forest, Artificial Neural Networks (ANN), a Stacked Ensemble model, and logistic regression (for baseline comparison). Hyperparameters for all individual models except the Stacked ensemble model were optimized via a random search (Supplementary Table S4). The Stacked Ensemble model was then constructed using these individually tuned models (CatBoost, XGBoost, LightGBM, Random Forest, ANN, and logistic regression) as base learners. Their predictions were combined using a logistic regression meta-learner with a regularization strength (C) of 0.1, a fixed random state for reproducibility, and a maximum iteration limit of 500. The tuning was guided by the area under the receiver operating characteristic curve (ROC-AUC) value, which was evaluated using 5-fold cross-validation on the training sets.

### Model performance and interpretation

For each prediction model developed based on the training data sets, performance metrics, including the ROC-AUC, accuracy, sensitivity, specificity, balanced accuracy (the average of sensitivity and specificity), positive predictive values (PPV), negative predictive values (NPV), and F1 scores (harmonic mean of PPV and sensitivity), were evaluated on the testing data sets using optimal threshold values. These metrics and their corresponding 95% confidence intervals were estimated using bootstrap resampling with 1,000 replicates. The optimal probability threshold for classifying a case as SGA was determined as the point on the ROC curve closest to the top-left corner (0,1). All metrics (e.g., sensitivity, specificity) are reported using this single, consistent threshold to facilitate model comparison. The best-fitting model was selected based on the following criteria: if all models had a precision-recall AUC (PR-AUC) below 0.2, the model with the highest ROC-AUC was chosen; otherwise, the model with the highest PR-AUC was selected. Calibration curves with a brier score were plotted to compare predicted and observed outcomes for the final optimal predictive model based on each birthweight standard. Model interpretation was performed by calculating Shapley Additive Explanation (SHAP) values on the testing datasets, employing a global approach to assess population-level feature importance. The mean absolute SHAP value was used to rank features by importance by their overall impact on the model output, while the distribution and central tendency of individual SHAP values (positive or negative) for each feature revealed its directional association with SGA risk. This analysis validated clinical relevance by confirming the alignment of top features with medical knowledge and used mean absolute SHAP values to rank features, identifying key determinants of SGA risk. To further evaluate model generalizability, an additional analysis was conducted using the complete data with a more stringent SGA definition. In this analysis, SGA status was defined by the overlap of all four birthweight standards, where a newborn was considered SGA only if classified as such by every standard, and all other births were defined as non-SGA. The optimal models identified under each individual standard were then evaluated when applied to identify SGA under this stringent, overlapping criterion. DeLong test was used to test the ROC-AUC difference between the best-fitting models, with P value < 0.001 considered statistically significant.

### Software and implementation

The analytical workflow was conducted using a dual-software approach. Data preprocessing and engineering were performed in R (version 3.6.1), which included multiple imputation via the MICE package to handle missing data, normalization and standardization using the recipes package with Yeo-Johnson transformation, and addressing class imbalance through the SMOTE algorithm implemented in the DMwR package. Subsequent predictive modeling and evaluation were implemented in Python 3.6 within the Spyder 6 environment, utilizing pandas and numpy for data manipulation, scikit-learn for machine learning algorithms and performance assessment, matplotlib and seaborn for visualization, SHAP for model interpretability, and scipy for statistical computations.

## Results

### SGA classification

**Figure 2:**
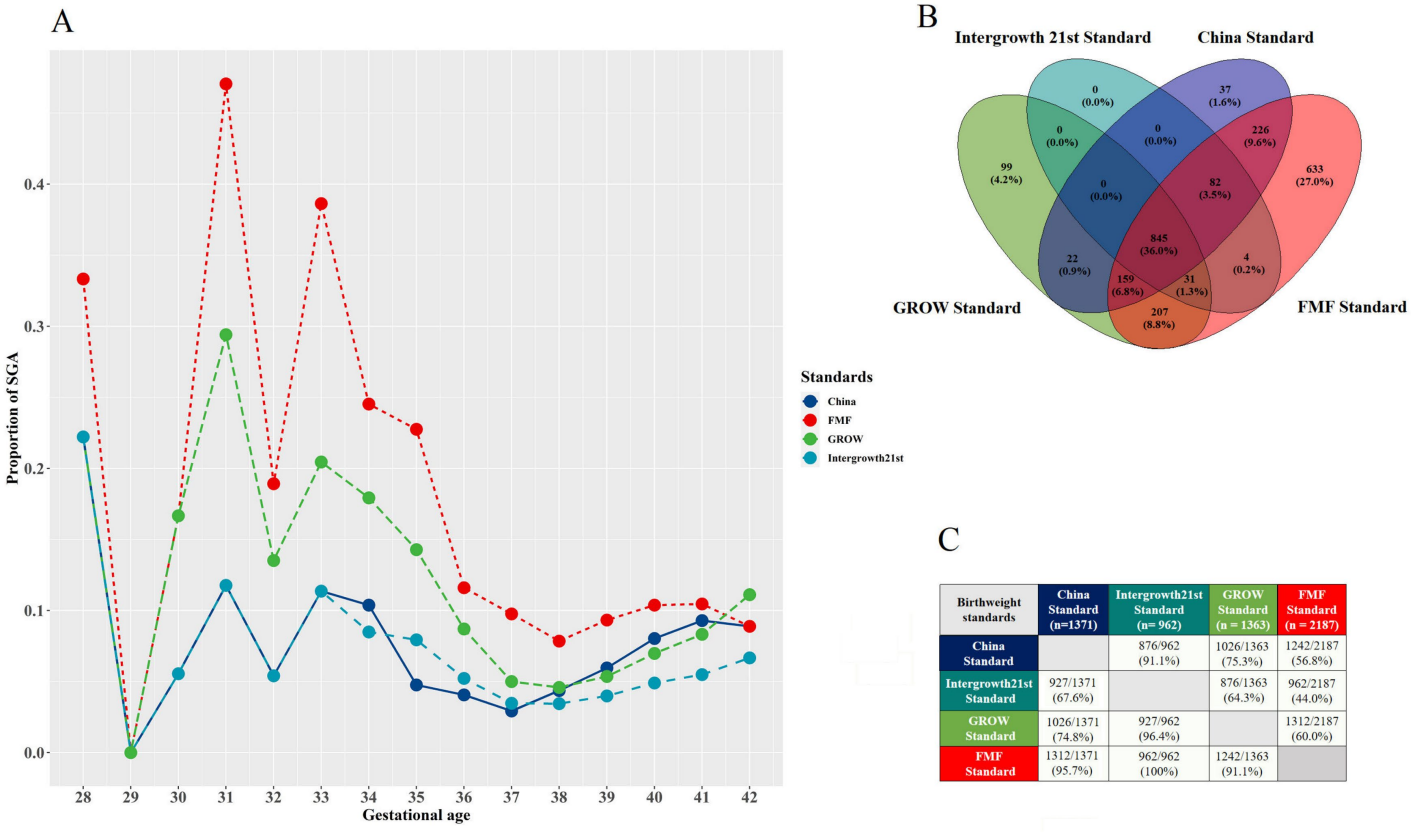
SGA classification according to different birthweight standards. **(A)** The proportion of SGA at birth at different gestational ages (in weeks) according to different birthweight for gestational age standards: China standard, Fetal Medicine Foundation (FMF) standard, Gestation-related Optimal Weight (GROW) standard, Intergrowth-21st standard. **(B)** Overlap of SGA cases classified according to the four birthweight standards. **(C)** Overlap of SGA cases classified according to pairs of birthweight standards. SGA, Small for gestational age (birthweight <10th centile for gestational age).

Among 22,603 singleton pregnancies with complete data, the rate of SGA with the China standard was 6.1%, which was similar to the GROW standard (6.0%), higher than the Intergrowth 21st standard (4.3%) and lower than the FMF standard (9.7%) (Table 1). Multiple imputation was performed for the cohort of 225,523 singleton pregnancies, with the distribution of variables before and after imputation compared in Supplementary Table S5. A similar trend in SGA rates across standards was observed in the larger imputed data (*n* = 225,523), with the China, GROW, Intergrowth 21st, and FMF standards yielding SGA rates of 6.8%, 7.4%, 4.8%, and 10.7%, respectively. SGA rates according to gestational age for each birthweight standard in 22,603 singleton pregnancies with complete data are shown in Figure 2A. There were similar proportions of SGA with the China and Intergrowth 21st standards at 28 to 37 weeks’ gestation, but a higher proportion of SGA with the China standard after 37 weeks. The GROW standard had intermediate rates of SGA before 37 weeks, but similar rates of SGA as the China standard after 37 weeks. The FMF standard classified the highest proportion of infants as SGA at all gestations (Figure 2A). 2,345 newborns were classified as SGA by at least one of the four standards, of which 845 (36.0%) were classified as SGA by all four standards (Figure 2B). 37 (1.6%) of infants were only classified as SGA by the China standard and not by any other standard (Figure 2B). The overlap of SGA cases classified by pairs of standards ranged from 44.0 to 100% (Figure 2C). SGA cases classified by the China standard were frequently also classified as SGA by the other three standards (67.6–95.7%) (Figure 2C). The overlap of non-SGA at birth classified by four birthweight standards is shown in Supplementary Figure S2.

**Table 1:** Comparison of maternal features in the complete data sets of four birthweight standards.

Features	China (SGA rate = 6.1%)			Intergrowth 21st (SGA rate = 4.3%)			GROW (SGA rate = 6.0%)			FMF (SGA rate = 9.7%)		
	Non-SGA	SGA	*p* value*	Non-SGA	SGA	*p* value*	Non-SGA	SGA	*p* value*	Non-SGA	SGA	*p* value*
*No.*	21,232	1,371		21,641	962		21,240	1,363		20,416	2,187	
Features collected at registration												
Gestational weeks at 1st visit	16.00 (13.00, 17.00)	16.00 (13.00, 17.00)	0.001	16.00 (13.00, 17.00)	16.00 (13.00, 17.00)	0.013	16.00 (13.00, 17.00)	16.00 (13.00, 17.00)	0.005	16.00 (13.00, 17.00)	16.00 (13.00, 17.00)	0.028
Maternal complications	80 (0.4)	10 (0.7)	0.074	81 (0.4)	9 (0.9)	0.015	78 (0.4)	12 (0.9)	0.007	76 (0.4)	14 (0.6)	0.087
Age at registration (y)	26.92 (4.54)	25.44 (4.20)	<0.001	26.89 (4.53)	25.44 (4.30)	<0.001	26.88 (4.52)	26.12 (4.56)	< 0.001	26.95 (4.54)	25.73 (4.30)	<0.001
Age at menarche (y)	14.06 (1.20)	14.14 (1.25)	0.017	14.06 (1.20)	14.19 (1.27)	0.001	14.06 (1.20)	14.18 (1.25)	< 0.001	14.06 (1.20)	14.12 (1.25)	0.021
Length of a menstrual cycle (days)	29.44 (2.59)	29.57 (2.60)	0.086	29.45 (2.59)	29.50 (2.67)	0.539	29.44 (2.59)	29.56 (2.66)	0.115	29.44 (2.59)	29.55 (2.61)	0.051
Length of a menstrual period	5.22 (1.36)	5.23 (1.40)	0.887	5.22 (1.36)	5.24 (1.41)	0.774	5.23 (1.36)	5.20 (1.39)	0.575	5.22 (1.36)	5.23 (1.39)	0.835
Occupation			0.283			0.709			0.636			0.314
Farmer or fishermen	8,097 (38.1%)	539 (39.3%)		8,252 (38.1%)	384 (39.9%)		8,093 (38.1%)	543 (39.8%)		7,790 (38.2%)	846 (38.7%)	
Employee	2,610 (12.3%)	142 (10.4%)		2,647 (12.2%)	105 (10.9%)		2,601 (12.2%)	151 (11.1%)		2,511 (12.3%)	241 (11.0%)	
Self-employed	2,438 (11.5%)	153 (11.2%)		2,483 (11.5%)	108 (11.2%)		2,437 (11.5%)	154 (11.3%)		2,354 (11.5%)	237 (10.8%)	
Stay at home without work	5,503 (25.9%)	369 (26.9%)		5,624 (26.0%)	248 (25.8%)		5,520 (26.0%)	352 (25.8%)		5,287 (25.9%)	585 (26.7%)	
Others	2,584 (12.2%)	168 (12.3%)		2,635 (12.2%)	117 (12.2%)		2,589 (12.2%)	163 (12.0%)		2,474 (12.1%)	278 (12.7%)	
Education			0.002			0.019			0.012			0.025
Primary school and below	972 (4.6%)	49 (3.6%)		989 (4.6%)	32 (3.3%)		966 (4.6%)	55 (4.0%)		941 (4.6%)	80 (3.7%)	
Secondary school and high school	12,961 (61.0%)	902 (65.8%)		13,235 (61.2%)	628 (65.3%)		12,975 (61.1%)	888 (65.2%)		12,472 (61.1%)	1,391 (63.6%)	
College and above	7,299 (34.4%)	420 (30.6%)		7,417 (34.3%)	302 (31.4%)		7,299 (34.4%)	420 (30.8%)		7,003 (34.3%)	716 (32.7%)	
Han Ethnicity	20,577 (96.9)	1,316 (96.0)	0.066	20,973 (96.9)	920 (95.6)	0.030	20,586 (96.9)	1,307 (95.9)	0.037	19,803 (97.0)	2090 (95.6)	<0.001
Smoking or Alcohol use	55 (0.3)	4 (0.3)	0.782	58 (0.3)	1 (0.1)	0.520	56 (0.3)	3 (0.2)	1.000	52 (0.3)	7 (0.3)	0.509
Contraception			0.117			0.372			0.268			0.087
Never	20,566 (96.9%)	1,332 (97.2%)		20,965 (96.9%)	933 (97.0%)		20,580 (96.9%)	1,318 (96.7%)		19,788 (96.9%)	2,110 (96.5%)	
Physical contraception	541 (2.5%)	26 (1.9%)		547 (2.5%)	20 (2.1%)		535 (2.5%)	32 (2.3%)		511 (2.5%)	56 (2.6%)	
Chemical contraception	119 (0.6%)	13 (0.9%)		123 (0.6%)	9 (0.9%)		119 (0.6%)	13 (1.0%)		111 (0.5%)	21 (1.0%)	
Both	6 (0.0%)	0 (0.0%)		6 (0.0%)	0 (0.0%)		6 (< 0.0%)	0 (0.0%)		6 (0.0%)	0 (0.0%)	
Medical history	259 (1.2%)	20 (1.5%)	0.447	262 (1.2%)	17 (1.8%)	0.133	258 (1.2%)	21 (1.5%)	0.309	250 (1.2%)	29 (1.3%)	0.683
Medicine use	185 (0.9%)	7 (0.5%)	0.221	187 (0.9%)	5 (0.5%)	0.365	183 (0.9%)	9 (0.7%)	0.542	177 (0.9%)	15 (0.7%)	0.461
Gynecological history	896 (4.2%)	40 (2.9%)	0.017	906 (4.2%)	30 (3.1%)	0.116	893 (4.2%)	43 (3.2%)	0.058	863 (4.2%)	73 (3.3%)	0.048
Parity			<0.001			<0.001			0.126			<0.001
0	11,595 (54.6%)	959 (69.9%)		11,879 (54.9%)	675 (70.2%)		11,761 (55.4%)	693 (58.2%)		11,081 (54.3%)	1,473 (67.4%)	
1	9,213 (43.4%)	394 (28.7%)		9,332 (43.1%)	275 (28.6%)		9,061 (42.7%)	546 (40.1%)		8,919 (43.7%)	688 (31.5%)	
>1	424 (2.0%)	18 (1.3%)		430 (2.0%)	12 (1.2%)		418 (2.0%)	24 (1.8%)		416 (2.0%)	26 (1.2%)	
Maternal height (cm)	159.45 (4.74)	157.87 (4.90)	<0.001	159.43 (4.74)	157.77 (4.98)	<0.001	159.36 (4.76)	159.37 (4.89)	0.922	159.51 (4.72)	157.93 (4.92)	<0.001
Maternal weight (kg)	53.38 (7.29)	50.33 (6.84)	<0.001	53.32 (7.29)	50.39 (6.95)	<0.001	53.19 (7.27)	53.21 (7.78)	0.924	53.49 (7.28)	50.44 (6.88)	<0.001
Heart rate (beats per minute)	80.38 (8.97)	80.65 (8.82)	0.270	80.38 (8.96)	80.71 (8.84)	0.266	80.38 (8.96)	80.63 (8.94)	0.317	80.37 (8.97)	80.55 (8.89)	0.371
Hemoglobin (g/L)	124.15 (9.85)	123.88 (10.25)	0.317	124.14 (9.85)	123.92 (10.42)	0.485	124.10 (9.85)	124.69 (10.21)	0.031	124.14 (9.82)	124.05 (10.37)	0.681
Leukocyte count (10^9/L)	8.14 (1.87)	8.03 (1.91)	0.045	8.13 (1.87)	8.08 (1.96)	0.397	8.13 (1.87)	8.08 (1.94)	0.304	8.14 (1.87)	8.06 (1.91)	0.056
Platelet count (10^9/L)	217.61 (47.14)	218.08 (47.51)	0.72	217.57 (47.07)	219.08 (49.12)	0.334	217.55 (47.11)	219.01 (47.91)	0.267	217.62 (47.20)	217.83 (46.79)	0.842
FBG (mmol/L)	4.70 (0.46)	4.67 (0.45)	0.017	4.70 (0.46)	4.66 (0.44)	0.022	4.69 (0.46)	4.69 (0.46)	0.789	4.70 (0.46)	4.67 (0.45)	0.016
ALT (U/L)	16.14 (10.16)	15.80 (9.91)	0.241	16.14 (10.16)	15.64 (9.82)	0.139	16.13 (10.16)	15.94 (9.99)	0.514	16.14 (10.18)	15.89 (9.89)	0.285
AST (U/L)	18.10 (6.06)	18.37 (6.14)	0.114	18.12 (6.07)	18.15 (5.98)	0.887	18.12 (6.07)	18.07 (6.05)	0.745	18.10 (6.07)	18.29 (6.03)	0.167
AIB (g/L)	41.89 (3.26)	42.28 (3.17)	<0.001	41.89 (3.25)	42.34 (3.27)	<0.001	41.89 (3.25)	42.26 (3.27)	<0.001	41.87 (3.26)	42.29 (3.21)	<0.001
TBil (mmol/L)	9.48 (3.66)	9.66 (3.85)	0.090	9.49 (3.66)	9.64 (3.79)	0.192	9.49 (3.66)	9.60 (3.78)	0.277	9.49 (3.66)	9.53 (3.77)	0.615
Scr (mmol/L)	49.16 (12.52)	49.20 (12.20)	0.907	49.17 (12.53)	49.04 (11.94)	0.761	49.14 (12.52)	49.58 (12.30)	0.202	49.16 (12.51)	49.20 (12.41)	0.894
BUN (mmol/L)	2.81 (0.76)	2.80 (0.75)	0.72	2.81 (0.76)	2.81 (0.77)	0.995	2.81 (0.76)	2.84 (0.76)	0.115	2.81 (0.76)	2.83 (0.78)	0.166
Features collected during antenatal visits												
Number of antenatal visits before 24 weeks	2.00 (2.00, 3.00)	2.00 (2.00, 3.00)	0.030	2.00 (2.00, 3.00)	2.00 (2.00, 3.00)	0.03	2.00 (2.00, 3.00)	2.00 (2.00, 3.00)	0.028	2.00 (2.00, 3.00)	2.00 (2.00, 3.00)	0.151
SBP1 (mmHg)	110.26 (11.24)	110.76 (11.69)	0.112	110.28 (11.24)	110.70 (11.86)	0.252	110.20 (11.22)	111.77 (11.83)	< 0.001	110.27 (11.23)	110.55 (11.57)	0.268
SBP2 (mmHg)	111.14 (10.68)	111.24 (11.16)	0.730	111.13 (10.67)	111.38 (11.40)	0.484	111.08 (10.67)	112.21 (11.23)	< 0.001	111.14 (10.69)	111.20 (10.86)	0.791
SBP3 (mmHg)	113.25 (9.29)	113.84 (10.54)	0.023	113.24 (9.29)	114.41 (10.94)	<0.001	113.19 (9.27)	114.80 (10.73)	< 0.001	113.24 (9.26)	113.74 (10.30)	0.017
DBP1 (mmHg)	67.02 (7.97)	67.62 (8.43)	0.006	67.03 (7.98)	67.65 (8.56)	0.018	66.97 (7.97)	68.36 (8.46)	< 0.001	67.00 (7.97)	67.51 (8.27)	0.005
DBP2 (mmHg)	66.29 (7.49)	66.87 (7.90)	0.006	66.30 (7.48)	66.98 (8.19)	0.006	66.25 (7.47)	67.52 (8.13)	< 0.001	66.28 (7.49)	66.81 (7.75)	0.002
DBP3 (mmHg)	68.05 (6.62)	69.15 (7.83)	<0.001	68.05 (6.62)	69.56 (8.27)	<0.001	68.01 (6.59)	69.77 (8.13)	<0.001	68.01 (6.60)	69.08 (7.58)	<0.001
WEIGHT1 (kg)	54.48 (7.48)	51.15 (7.20)	<0.001	54.42 (7.49)	51.17 (7.25)	<0.001	54.30 (7.47)	54.02 (7.98)	0.191	54.60 (7.47)	51.27 (7.15)	<0.001
WEIGHT2 (kg)	57.89 (7.52)	53.98 (7.19)	<0.001	57.82 (7.53)	54.00 (7.27)	<0.001	57.71 (7.53)	56.71 (7.90)	<0.001	58.03 (7.51)	54.12 (7.06)	<0.001
WEIGHT3 (kg)	63.64 (7.84)	59.31 (7.56)	<0.001	63.55 (7.85)	59.32 (7.76)	<0.001	63.46 (7.85)	61.99 (8.38)	<0.001	63.80 (7.82)	59.44 (7.53)	<0.001
SFH2 (cm)	21.43 (2.29)	20.78 (2.33)	<0.001	21.42 (2.30)	20.78 (2.32)	<0.001	21.42 (2.29)	20.87 (2.36)	<0.001	21.45 (2.29)	20.79 (2.30)	<0.001
SFH3 (cm)	30.08 (2.26)	28.81 (2.27)	<0.001	30.06 (2.27)	28.71 (2.23)	<0.001	30.08 (2.27)	28.88 (2.26)	<0.001	30.13 (2.25)	28.87 (2.25)	<0.001
MAC2 (cm)	83.78 (6.28)	80.64 (6.11)	<0.001	83.72 (6.29)	80.69 (6.16)	<0.001	83.67 (6.29)	82.44 (6.62)	<0.001	83.89 (6.27)	80.82 (6.10)	<0.001
MAC3 (cm)	92.59 (5.92)	88.94 (5.78)	<0.001	92.52 (5.93)	88.80 (5.74)	<0.001	92.49 (5.93)	90.37 (6.22)	<0.001	92.72 (5.90)	89.07 (5.60)	<0.001
diffSBP12 (mmHg)	0.50 (−5.00, 7.00)	0.00 (−5.50, 6.00)	0.034	0.50 (−5.00, 7.00)	0.00 (−5.50, 6.50)	0.248	0.50 (−5.00, 7.00)	0.00 (−5.50, 6.00)	0.029	0.55 (−5.00, 7.00)	0.00 (−5.50, 6.50)	0.092
diffSBP23 (mmHg)	2.10 (−3.33, 7.50)	2.60 (−3.32, 8.00)	0.066	2.00 (−3.33, 7.50)	3.00 (−2.79, 8.50)	0.002	2.12 (−3.33, 7.50)	2.33 (−2.79, 8.00)	0.167	2.10 (−3.33, 7.50)	2.40 (−3.10, 7.80)	0.090
diffSBP13 ((mmHg))	3.00 (−3.50, 9.50)	3.00 (−3.00, 9.33)	0.770	3.00 (−3.50, 9.50)	3.45 (−2.33, 9.96)	0.026	3.00 (−3.50, 9.50)	2.67 (−3.33, 9.00)	0.746	3.00 (−3.50, 9.50)	2.80 (−3.18, 9.45)	0.514
diffDBP12 ((mmHg))	−0.50 (−5.00, 4.00)	−0.50 (−5.00, 3.50)	0.570	−0.50 (−5.00, 4.00)	−0.50 (−5.50, 3.50)	0.633	−0.50 (−5.00, 4.00)	−0.50 (−5.25, 3.50)	0.352	−0.50 (−5.00, 4.00)	−0.50 (−5.17, 4.00)	0.846
diffDBP23 (mmHg)	1.75 (−2.00, 5.67)	2.40 (−1.54, 6.27)	0.002	1.75 (−2.00, 5.67)	2.80 (−1.33, 6.80)	<0.001	1.75 (−2.00, 5.67)	2.25 (−1.75, 6.31)	0.010	1.75 (−2.00, 5.67)	2.33 (−1.75, 6.27)	<0.001
diffDBP13 (mmHg)	1.00 (−3.75, 5.83)	1.17 (−3.33, 6.15)	0.061	1.00 (−3.75, 5.80)	1.67 (−3.19, 6.67)	0.003	1.00 (−3.75, 5.83)	1.00 (−3.50, 6.29)	0.251	1.00 (−3.80, 5.75)	1.33 (−3.50, 6.50)	0.007
diffWEIGHT12 (kg)	3.25 (2.25, 4.50)	2.85 (2.00, 3.99)	<0.001	3.25 (2.25, 4.50)	2.85 (2.00, 3.85)	<0.001	3.25 (2.25, 4.50)	2.75 (1.77, 3.75)	<0.001	3.30 (2.25, 4.50)	3.00 (2.00, 4.00)	<0.001
diffWEIGHT23 (kg)	5.62 (4.14, 7.17)	5.10 (3.91, 6.63)	<0.001	5.62 (4.14, 7.17)	5.10 (3.83, 6.56)	<0.001	5.63 (4.14, 7.17)	5.10 (3.75, 6.52)	<0.001	5.67 (4.17, 7.17)	5.10 (3.83, 6.60)	<0.001
diffWEIGHT13 (kg)	9.00 (7.07, 11.10)	8.00 (6.25, 10.00)	<0.001	9.00 (7.05, 11.08)	8.00 (6.25, 10.00)	<0.001	9.00 (7.08, 11.10)	7.85 (6.03, 10.00)	<0.001	9.00 (7.10, 11.13)	8.00 (6.33, 10.00)	<0.001
diffSFH23 (cm)	8.65 (7.17, 10.00)	8.00 (6.50, 9.50)	<0.001	8.62 (7.14, 10.00)	7.95 (6.40, 9.47)	<0.001	8.64 (7.17, 10.00)	8.00 (6.40, 9.50)	<0.001	8.67 (7.17, 10.07)	8.00 (6.50, 9.50)	<0.001
diffMAC23 (cm)	8.75 (6.67, 10.86)	8.29 (6.25, 10.25)	<0.001	8.75 (6.67, 10.86)	8.12 (6.00, 10.00)	<0.001	8.80 (6.75, 10.90)	8.00 (5.67, 10.00)	<0.001	8.80 (6.71, 10.90)	8.25 (6.17, 10.33)	<0.001

Data are *n* (%), mean (SD), or median (IQR). AIB, Albumin; ALT, Alanine aminotransferase; AST, Aspartate aminotransferase; BUN, Serum urea nitrogen; DBP, diastolic blood pressure; FBG, fasting blood glucose; MAC, maternal abdominal circumference; Maternal complications means clinical diagnosis with previa, eclampsia, or pregnancy-induced hypertension; SBP, systolic blood pressure; Scr, Serum creatinine; SGA, small for gestational age (birthweight <10th centile for gestational age); TBil, Total bilirubin; diffSBDP12, difference between SBP1 and SBP2.*p values were calculated with the student Student’s t test for normalized continuous variables, the Mann–Whitney U test for non-normalized continuous variables, the chi-squared test for categorical variables when all of the cells have counts more than 5, and the Fisher’s exact test for categorical variables when some of the cells have counts less than 5.

Significant differences in maternal age, weight, age at menarche, education and albumin at registration were observed between pregnancies with SGA and non-SGA infants for the China standard and the three other birthweight standards (Table 1). Blood pressure values (SBP3, DBP1, DBP2, DBP3), blood pressure change values (diffDBP23), and all maternal anthropometric measurements (maternal weight, MAC, and SFH) and their change values between each two pregnancy intervals differed significantly between pregnancies with SGA infants compared to non-SGA infants for all birthweight standards (Table 1).

### SGA prediction modeling

For each birthweight standard, feature selection was conducted using lasso regression, separately across three pregnancy intervals: early (<18 weeks), middle (<26 weeks), and late pregnancy (<37 weeks). The analysis was performed on both imputed and complete datasets, with the optimal *λ* value selected using the one-standard-error criterion. The number of predictors retained for each standard and pregnancy interval, along with the corresponding λ values, are summarized in Supplementary Table S6. The variable selection paths and importance rankings across all four birthweight standards are illustrated in Supplementary Figures S3–S6, which present coefficient shrinkage plots and variable importance bar charts for each pregnancy interval.

**Figure 3:**
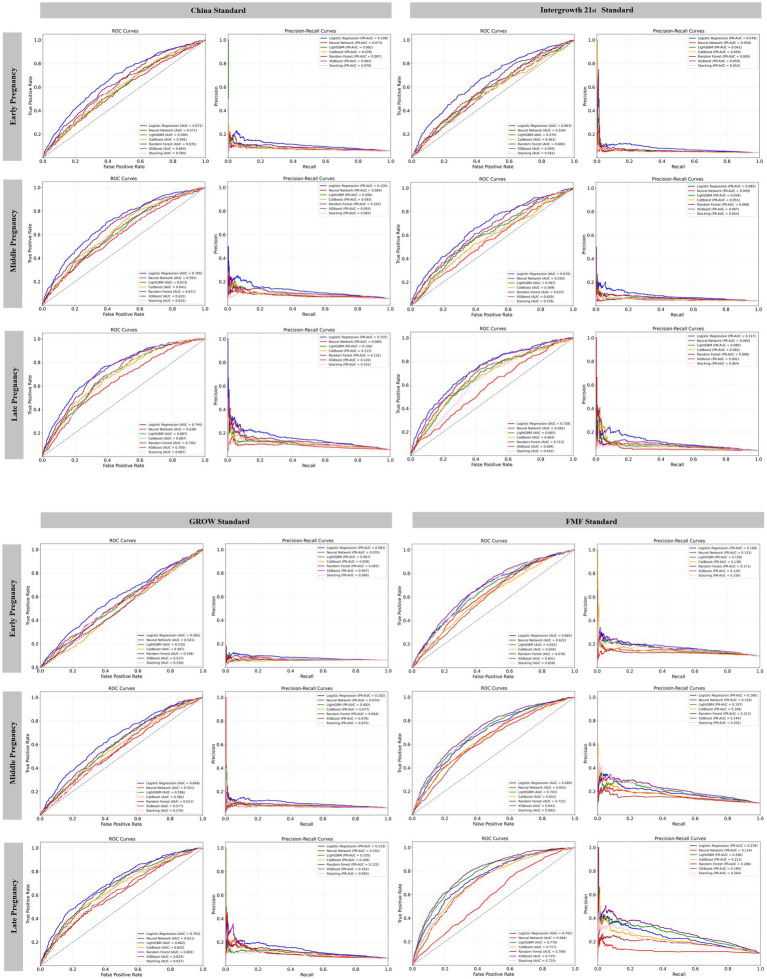
Receiver operating characteristic and precision-recall curves for prediction of small for gestational age at three pregnancy intervals in the testing set of complete data according to four birthweight standards using six machine learning algorithms and logistic regression prediction models. PR-AUC, Area under the precision-recall curve; ROC, Receiver Operating Characteristic.

ROC curves and PR-ROC curves for different pregnancy intervals in the testing sets are illustrated in Figure 3 for the complete data and Supplementary Figure S7 for the imputed data, respectively. Late pregnancy prediction models performed better at ROC-AUCs than early and middle pregnancy models for all birthweight standards (Figure 3; Table 2). The China standard had intermediate predictive ROC-AUCs for SGA across the three pregnancy intervals and ML models, with ROC-AUCs similar to the Intergrowth 21st standard, better than the GROW standard, but not as good as the FMF standard (Figure 3; Table 2). The highest ROC-AUC values observed for the late pregnancy models were 0.74 for logistic regression with the China standard, and ROC-AUCs ranging from 0.64 to 0.79 for the other standards (Table 2). For the China standard, the late pregnancy model developed by the logistic regression had the highest F1 score, with a value of 0.30. Based on predefined criteria, the best performing model was the late pregnancy model based on logistic regression for the China standard (ROC-AUC 0.74, PR-AUC 0.16), and the late pregnancy model based on Random Forest showed superior performance for the FMF standard (ROC-AUC 0.79, PR-AUC 0.28), with sensitivity of 0.78, PPV of 0.20, and F1 score of 0.45. Their calibration curves and hyper-parameter settings are shown in Supplementary Figure S8 and Supplementary Table S7, respectively. The calibration curves of the top-performing models (Supplementary Figure S8) demonstrated systematic overestimation, deviating above the line of perfect calibration. This is evidenced by Brier scores of 0.2281 (China standard, logistic regression), 0.2359 (INTERGROWTH-21st standard, logistic regression), 0.2325 (GROW standard, logistic regression), and 0.1949 (FMF standard, Random Forest). The model for the FMF standard exhibited the best calibration. The ROC curves of the training set and testing set of the complete data indicated consistent predictive performance for the China, INTERGROWTH-21st, and GROW standards, with minimal AUC differences. However, a more notable performance gap was observed for the FMF standard (training AUC: 0.982, testing AUC: 0.789), suggesting a degree of overfitting for this specific model (Supplementary Figure S9). The predictive performance of models developed using the imputed dataset was largely consistent with that observed in the complete dataset, showing similar trends across pregnancy intervals and birthweight standards. The optimal models for the China, Intergrowth 21st, and FMF standards remained logistic regression, while XGBoost performed best for the GROW standard, with the performance, hyper-parameter settings, and ROC curves of the training set and testing set provided in Supplementary Tables S8, S9 and Supplementary Figure S10, respectively.

**Table 2:** Bootstrap Validation of prediction model performance using testing data set from the complete data.

Models	China standard							Intergrowth 21st standard						
	AUC (95% CI)	Sen. (95% CI)	Spe. (95% CI)	Acc. (95% CI)	PPV (95% CI)	NPV (95% CI)	F1 score (95% CI)	AUC (95% CI)	Sen. (95% CI)	Spe. (95% CI)	Acc. (95% CI)	PPV (95% CI)	NPV (95% CI)	F1 score (95% CI)
Early pregnancy														
Catboost	0.59 (0.56, 0.62)	0.70 (0.46, 0.84)	0.46 (0.30, 0.68)	0.47 (0.33, 0.67)	0.07 (0.06, 0.08)	0.96 (0.95, 0.97)	0.16 (0.11, 0.20)	0.56 (0.53, 0.60)	0.46 (0.29, 0.61)	0.68 (0.55, 0.84)	0.67 (0.55, 0.82)	0.06 (0.05, 0.08)	0.97 (0.96, 0.97)	0.14 (0.09, 0.20)
XGBoost	0.60 (0.58, 0.63)	0.62 (0.46, 0.72)	0.57 (0.47, 0.72)	0.57 (0.48, 0.71)	0.08 (0.07, 0.10)	0.96 (0.95, 0.97)	0.19 (0.14, 0.24)	0.59 (0.56, 0.63)	0.52 (0.31, 0.78)	0.64 (0.36, 0.81)	0.64 (0.38, 0.79)	0.06 (0.05, 0.08)	0.97 (0.96, 0.98)	0.16 (0.11, 0.22)
LightBoost	0.59 (0.56, 0.62)	0.63 (0.36, 0.84)	0.52 (0.29, 0.78)	0.52 (0.32, 0.76)	0.07 (0.06, 0.09)	0.96 (0.95, 0.97)	0.15 (0.10, 0.18)	0.57 (0.54, 0.61)	0.59 (0.45, 0.84)	0.55 (0.31, 0.66)	0.55 (0.33, 0.65)	0.05 (0.04, 0.07)	0.97 (0.96, 0.98)	0.14 (0.09, 0.20)
Random Forest	0.64 (0.61, 0.67)	0.66 (0.55, 0.87)	0.56 (0.34, 0.66)	0.57 (0.37, 0.65)	0.08 (0.07, 0.10)	0.97 (0.96, 0.98)	0.22 (0.18, 0.27)	0.60 (0.57, 0.63)	0.74 (0.54, 0.85)	0.44 (0.35, 0.61)	0.45 (0.37, 0.61)	0.05 (0.05, 0.06)	0.98 (0.97, 0.98)	0.18 (0.12, 0.22)
ANN	0.57 (0.55, 0.60)	0.57 (0.39, 0.69)	0.57 (0.48, 0.73)	0.57 (0.49, 0.71)	0.08 (0.07, 0.09)	0.96 (0.95, 0.96)	0.15 (0.10, 0.20)	0.58 (0.54, 0.61)	0.67 (0.32, 0.86)	0.48 (0.31, 0.79)	0.48 (0.33, 0.77)	0.05 (0.04, 0.07)	0.97 (0.96, 0.98)	0.14 (0.09, 0.19)
Stacking ensemble	0.59 (0.56, 0.62)	0.57 (0.49, 0.66)	0.61 (0.51, 0.66)	0.60 (0.52, 0.65)	0.08 (0.07, 0.09)	0.96 (0.95, 0.96)	0.17 (0.13, 0.22)	0.56 (0.53, 0.59)	0.60 (0.34, 0.82)	0.51 (0.29, 0.78)	0.52 (0.31, 0.76)	0.05 (0.04, 0.06)	0.97 (0.96, 0.98)	0.12 (0.07, 0.16)
Logistic regression	0.67 (0.65, 0.7)	0.68 (0.55, 0.76)	0.60 (0.49, 0.73)	0.60 (0.5, 0.72)	0.09 (0.08, 0.12)	0.97 (0.96, 0.97)	0.27 (0.24, 0.31)	0.66 (0.62, 0.69)	0.64 (0.48, 0.81)	0.62 (0.44, 0.76)	0.62 (0.46, 0.75)	0.07 (0.05, 0.09)	0.98 (0.97, 0.98)	0.26 (0.20, 0.31)
Middle pregnancy														
Catboost	0.64 (0.62, 0.67)	0.74 (0.45, 0.83)	0.49 (0.41, 0.78)	0.50 (0.43, 0.76)	0.08 (0.07, 0.11)	0.97 (0.96, 0.98)	0.23 (0.19, 0.27)	0.57 (0.54, 0.60)	0.50 (0.30, 0.60)	0.65 (0.53, 0.83)	0.64 (0.53, 0.81)	0.06 (0.05, 0.07)	0.97 (0.96, 0.97)	0.14 (0.09, 0.21)
XGBoost	0.62 (0.60, 0.65)	0.68 (0.50, 0.78)	0.52 (0.42, 0.70)	0.53 (0.44, 0.68)	0.08 (0.07, 0.09)	0.96 (0.96, 0.97)	0.20 (0.16, 0.25)	0.62 (0.59, 0.66)	0.52 (0.35, 0.68)	0.68 (0.54, 0.84)	0.67 (0.54, 0.82)	0.07 (0.05, 0.08)	0.97 (0.97, 0.98)	0.20 (0.15, 0.27)
LightBoost	0.62 (0.60, 0.65)	0.69 (0.37, 0.86)	0.51 (0.34, 0.81)	0.52 (0.37, 0.79)	0.08 (0.07, 0.11)	0.97 (0.95, 0.98)	0.19 (0.16, 0.24)	0.60 (0.56, 0.63)	0.55 (0.45, 0.65)	0.63 (0.56, 0.72)	0.63 (0.57, 0.71)	0.06 (0.05, 0.07)	0.97 (0.97, 0.98)	0.19 (0.13, 0.24)
Random Forest	0.67 (0.65, 0.70)	0.70 (0.61, 0.80)	0.57 (0.48, 0.65)	0.58 (0.50, 0.65)	0.09 (0.08, 0.1)	0.97 (0.96, 0.98)	0.28 (0.24, 0.33)	0.64 (0.60, 0.67)	0.65 (0.54, 0.87)	0.58 (0.34, 0.68)	0.58 (0.36, 0.67)	0.06 (0.05, 0.07)	0.98 (0.97, 0.98)	0.23 (0.17, 0.27)
ANN	0.60 (0.57, 0.63)	0.65 (0.46, 0.85)	0.51 (0.28, 0.70)	0.51 (0.31, 0.69)	0.07 (0.06, 0.09)	0.96 (0.95, 0.97)	0.16 (0.12, 0.20)	0.56 (0.52, 0.59)	0.53 (0.33, 0.87)	0.59 (0.21, 0.76)	0.59 (0.24, 0.74)	0.05 (0.04, 0.07)	0.97 (0.96, 0.97)	0.12 (0.07, 0.17)
Stacking ensemble	0.62 (0.59, 0.65)	0.53 (0.39, 0.86)	0.67 (0.30, 0.79)	0.66 (0.33, 0.76)	0.09 (0.07, 0.10)	0.96 (0.95, 0.97)	0.19 (0.15, 0.24)	0.58 (0.55, 0.61)	0.54 (0.35, 0.87)	0.60 (0.25, 0.78)	0.6 (0.28, 0.76)	0.06 (0.04, 0.07)	0.97 (0.96, 0.98)	0.14 (0.09, 0.19)
Logistic regression	0.70 (0.68, 0.73)	0.70 (0.58, 0.78)	0.62 (0.56, 0.75)	0.63 (0.57, 0.74)	0.10 (0.09, 0.12)	0.97 (0.97, 0.98)	0.32 (0.28, 0.37)	0.67 (0.66, 0.68)	0.56 (0.52, 0.63)	0.69 (0.63, 0.72)	0.68 (0.63, 0.71)	0.09 (0.08, 0.09)	0.97 (0.97, 0.97)	0.25 (0.24, 0.27)
Late pregnancy														
Catboost	0.69 (0.66, 0.71)	0.65 (0.48, 0.78)	0.63 (0.49, 0.80)	0.64 (0.51, 0.78)	0.10 (0.08, 0.13)	0.97 (0.96, 0.97)	0.29 (0.25, 0.33)	0.66 (0.63, 0.70)	0.61 (0.52, 0.71)	0.67 (0.57, 0.72)	0.67 (0.57, 0.72)	0.07 (0.06, 0.09)	0.98 (0.97, 0.98)	0.29 (0.23, 0.35)
XGBoost	0.70 (0.68, 0.72)	0.77 (0.62, 0.88)	0.54 (0.44, 0.68)	0.56 (0.46, 0.68)	0.09 (0.08, 0.11)	0.98 (0.97, 0.98)	0.32 (0.28, 0.36)	0.68 (0.65, 0.72)	0.71 (0.52, 0.79)	0.60 (0.53, 0.78)	0.61 (0.54, 0.77)	0.07 (0.06, 0.09)	0.98 (0.97, 0.98)	0.32 (0.26, 0.37)
LightBoost	0.69 (0.66, 0.71)	0.70 (0.58, 0.84)	0.60 (0.45, 0.70)	0.60 (0.47, 0.70)	0.10 (0.08, 0.11)	0.97 (0.96, 0.98)	0.30 (0.26, 0.34)	0.66 (0.63, 0.70)	0.69 (0.57, 0.79)	0.59 (0.52, 0.73)	0.59 (0.53, 0.72)	0.07 (0.06, 0.08)	0.98 (0.97, 0.98)	0.28 (0.22, 0.34)
Random Forest	0.73 (0.71, 0.76)	0.72 (0.64, 0.82)	0.65 (0.54, 0.72)	0.65 (0.56, 0.72)	0.11 (0.09, 0.13)	0.98 (0.97, 0.98)	0.37 (0.33, 0.42)	0.71 (0.68, 0.75)	0.74 (0.65, 0.82)	0.61 (0.54, 0.69)	0.62 (0.56, 0.69)	0.08 (0.06, 0.09)	0.98 (0.98, 0.99)	0.35 (0.30, 0.41)
ANN	0.64 (0.61, 0.66)	0.59 (0.52, 0.66)	0.63 (0.57, 0.71)	0.63 (0.57, 0.70)	0.09 (0.08, 0.10)	0.96 (0.96, 0.97)	0.23 (0.18, 0.28)	0.66 (0.62, 0.69)	0.63 (0.41, 0.83)	0.61 (0.40, 0.81)	0.61 (0.42, 0.80)	0.07 (0.05, 0.09)	0.98 (0.97, 0.98)	0.24 (0.20, 0.29)
Stacking ensemble	0.69 (0.66, 0.71)	0.72 (0.65, 0.86)	0.58 (0.42, 0.63)	0.59 (0.44, 0.63)	0.09 (0.08, 0.11)	0.97 (0.97, 0.98)	0.30 (0.25, 0.35)	0.64 (0.61, 0.67)	0.69 (0.59, 0.80)	0.56 (0.49, 0.65)	0.57 (0.50, 0.65)	0.06 (0.05, 0.08)	0.98 (0.97, 0.98)	0.25 (0.19, 0.31)
Logistic regression	0.74 (0.72, 0.77)	0.74 (0.60, 0.79)	0.65 (0.61, 0.78)	0.65 (0.62, 0.77)	0.11 (0.10, 0.14)	0.98 (0.97, 0.98)	0.39 (0.35, 0.43)	0.73 (0.69, 0.76)	0.71 (0.59, 0.83)	0.65 (0.50, 0.75)	0.65 (0.52, 0.74)	0.08 (0.06, 0.10)	0.98 (0.98, 0.99)	0.36 (0.30, 0.40)

Models	GROW Standard							FMF Standard						
	AUC (95% CI)	Sen. (95% CI)	Spe. (95% CI)	Acc. (95% CI)	PPV (95% CI)	NPV (95% CI)	F1 score (95% CI)	AUC (95% CI)	Sen. (95% CI)	Spe. (95% CI)	Acc. (95% CI)	PPV (95% CI)	NPV (95% CI)	F1 score (95% CI)
Early pregnancy														
Catboost	0.50 (0.47, 0.52)	0.75 (0.43, 0.94)	0.29 (0.09, 0.62)	0.32 (0.15, 0.61)	0.06 (0.06, 0.07)	0.95 (0.94, 0.97)	0.04 (0.02, 0.07)	0.60 (0.58, 0.62)	0.61 (0.47, 0.71)	0.56 (0.46, 0.69)	0.56 (0.48, 0.67)	0.13 (0.12, 0.14)	0.93 (0.92, 0.94)	0.17 (0.13, 0.20)
XGBoost	0.52 (0.49, 0.54)	0.41 (0.11, 0.90)	0.65 (0.16, 0.93)	0.64 (0.21, 0.88)	0.08 (0.06, 0.11)	0.95 (0.94, 0.96)	0.06 (0.04, 0.09)	0.60 (0.59, 0.62)	0.77 (0.64, 0.87)	0.41 (0.29, 0.52)	0.44 (0.35, 0.54)	0.12 (0.11, 0.13)	0.94 (0.93, 0.96)	0.17 (0.14, 0.21)
LightBoost	0.54 (0.51, 0.57)	0.57 (0.27, 0.81)	0.52 (0.27, 0.81)	0.52 (0.30, 0.78)	0.07 (0.06, 0.09)	0.95 (0.94, 0.96)	0.09 (0.05, 0.13)	0.65 (0.63, 0.67)	0.65 (0.55, 0.76)	0.58 (0.47, 0.67)	0.59 (0.50, 0.66)	0.14 (0.13, 0.16)	0.94 (0.93, 0.95)	0.24 (0.20, 0.27)
Random Forest	0.54 (0.51, 0.56)	0.60 (0.41, 0.88)	0.49 (0.20, 0.66)	0.50 (0.23, 0.65)	0.07 (0.06, 0.08)	0.95 (0.94, 0.97)	0.09 (0.05, 0.13)	0.68 (0.66, 0.70)	0.69 (0.56, 0.79)	0.59 (0.49, 0.70)	0.60 (0.52, 0.69)	0.15 (0.14, 0.18)	0.95 (0.94, 0.96)	0.28 (0.25, 0.31)
ANN	0.52 (0.49, 0.55)	0.42 (0.15, 0.79)	0.65 (0.28, 0.89)	0.64 (0.32, 0.85)	0.08 (0.06, 0.10)	0.95 (0.94, 0.96)	0.07 (0.03, 0.10)	0.63 (0.61, 0.66)	0.58 (0.51, 0.75)	0.64 (0.46, 0.71)	0.64 (0.49, 0.69)	0.15 (0.13, 0.16)	0.93 (0.93, 0.95)	0.22 (0.19, 0.26)
Stacking ensemble	0.54 (0.51, 0.56)	0.47 (0.26, 0.75)	0.61 (0.32, 0.81)	0.60 (0.35, 0.78)	0.07 (0.06, 0.09)	0.95 (0.94, 0.95)	0.08 (0.04, 0.12)	0.66 (0.64, 0.68)	0.70 (0.59, 0.81)	0.56 (0.45, 0.65)	0.57 (0.48, 0.65)	0.14 (0.13, 0.16)	0.95 (0.94, 0.96)	0.26 (0.23, 0.29)
Logistic regression	0.58 (0.56, 0.61)	0.53 (0.32, 0.65)	0.62 (0.5, 0.82)	0.62 (0.5, 0.79)	0.08 (0.07, 0.1)	0.95 (0.95, 0.96)	0.15 (0.11, 0.20)	0.66 (0.64, 0.68)	0.66 (0.59, 0.73)	0.62 (0.54, 0.66)	0.62 (0.56, 0.66)	0.15 (0.14, 0.17)	0.94 (0.94, 0.95)	0.27 (0.24, 0.31)
Middle pregnancy														
Catboost	0.58 (0.55, 0.61)	0.54 (0.36, 0.88)	0.60 (0.23, 0.77)	0.60 (0.27, 0.74)	0.08 (0.07, 0.10)	0.95 (0.95, 0.97)	0.14 (0.09, 0.18)	0.65 (0.63, 0.67)	0.59 (0.47, 0.77)	0.65 (0.49, 0.77)	0.64 (0.52, 0.74)	0.15 (0.13, 0.18)	0.94 (0.93, 0.95)	0.24 (0.20, 0.28)
XGBoost	0.58 (0.55, 0.60)	0.82 (0.39, 0.94)	0.31 (0.20, 0.74)	0.34 (0.25, 0.72)	0.07 (0.06, 0.09)	0.97 (0.95, 0.98)	0.14 (0.11, 0.17)	0.64 (0.62, 0.66)	0.67 (0.61, 0.78)	0.56 (0.44, 0.62)	0.57 (0.47, 0.62)	0.14 (0.13, 0.16)	0.94 (0.93, 0.95)	0.23 (0.20, 0.27)
LightBoost	0.60 (0.57, 0.62)	0.68 (0.53, 0.82)	0.49 (0.35, 0.64)	0.50 (0.38, 0.63)	0.08 (0.07, 0.09)	0.96 (0.95, 0.97)	0.17 (0.12, 0.21)	0.70 (0.68, 0.72)	0.68 (0.53, 0.79)	0.63 (0.52, 0.78)	0.63 (0.55, 0.76)	0.17 (0.14, 0.21)	0.95 (0.94, 0.96)	0.31 (0.28, 0.34)
Random Forest	0.61 (0.58, 0.63)	0.62 (0.41, 0.83)	0.56 (0.33, 0.77)	0.56 (0.36, 0.75)	0.08 (0.07, 0.10)	0.96 (0.95, 0.97)	0.17 (0.13, 0.21)	0.72 (0.70, 0.74)	0.70 (0.56, 0.80)	0.64 (0.55, 0.77)	0.65 (0.57, 0.75)	0.18 (0.15, 0.21)	0.95 (0.94, 0.96)	0.34 (0.31, 0.38)
ANN	0.56 (0.54, 0.59)	0.69 (0.40, 0.88)	0.43 (0.25, 0.71)	0.45 (0.29, 0.69)	0.07 (0.06, 0.09)	0.96 (0.95, 0.97)	0.12 (0.09, 0.16)	0.68 (0.66, 0.70)	0.62 (0.54, 0.68)	0.70 (0.65, 0.77)	0.69 (0.65, 0.75)	0.18 (0.16, 0.21)	0.95 (0.94, 0.95)	0.32 (0.28, 0.35)
Stacking ensemble	0.57 (0.55, 0.60)	0.73 (0.57, 0.87)	0.41 (0.27, 0.55)	0.43 (0.31, 0.55)	0.07 (0.07, 0.08)	0.96 (0.95, 0.97)	0.14 (0.10, 0.18)	0.69 (0.67, 0.71)	0.65 (0.60, 0.73)	0.67 (0.59, 0.71)	0.66 (0.60, 0.70)	0.17 (0.15, 0.19)	0.95 (0.94, 0.95)	0.32 (0.28, 0.35)
Logistic regression	0.65 (0.62, 0.67)	0.63 (0.41, 0.77)	0.60 (0.45, 0.78)	0.60 (0.47, 0.76)	0.09 (0.08, 0.11)	0.96 (0.95, 0.97)	0.23 (0.18, 0.28)	0.69 (0.67, 0.71)	0.65 (0.54, 0.78)	0.65 (0.52, 0.74)	0.65 (0.54, 0.72)	0.17 (0.14, 0.19)	0.95 (0.94, 0.96)	0.29 (0.26, 0.33)
Late pregnancy														
Catboost	0.65 (0.63, 0.68)	0.54 (0.46, 0.74)	0.71 (0.52, 0.77)	0.70 (0.53, 0.75)	0.11 (0.09, 0.13)	0.96 (0.95, 0.97)	0.25 (0.21, 0.30)	0.72 (0.69, 0.74)	0.69 (0.57, 0.78)	0.65 (0.56, 0.76)	0.65 (0.58, 0.75)	0.18 (0.15, 0.21)	0.95 (0.94, 0.96)	0.34 (0.30, 0.38)
XGBoost	0.62 (0.60, 0.65)	0.55 (0.44, 0.78)	0.64 (0.41, 0.75)	0.64 (0.43, 0.73)	0.09 (0.07, 0.11)	0.96 (0.95, 0.97)	0.20 (0.15, 0.24)	0.71 (0.70, 0.73)	0.73 (0.64, 0.78)	0.62 (0.58, 0.69)	0.63 (0.59, 0.69)	0.17 (0.15, 0.19)	0.95 (0.95, 0.96)	0.34 (0.31, 0.38)
LightBoost	0.66 (0.64, 0.69)	0.68 (0.51, 0.80)	0.58 (0.46, 0.75)	0.59 (0.48, 0.74)	0.10 (0.08, 0.11)	0.97 (0.96, 0.97)	0.26 (0.22, 0.30)	0.77 (0.75, 0.79)	0.71 (0.63, 0.83)	0.71 (0.60, 0.78)	0.71 (0.62, 0.77)	0.21 (0.17, 0.24)	0.96 (0.95, 0.97)	0.42 (0.38, 0.45)
Random Forest	0.68 (0.66, 0.70)	0.61 (0.53, 0.74)	0.67 (0.53, 0.73)	0.66 (0.55, 0.72)	0.11 (0.09, 0.12)	0.96 (0.96, 0.97)	0.28 (0.24, 0.32)	0.79 (0.77, 0.81)	0.78 (0.71, 0.83)	0.67 (0.63, 0.75)	0.68 (0.65, 0.75)	0.20 (0.18, 0.24)	0.97 (0.96, 0.97)	0.45 (0.42, 0.49)
ANN	0.61 (0.58, 0.63)	0.57 (0.42, 0.79)	0.60 (0.39, 0.73)	0.59 (0.42, 0.72)	0.09 (0.07, 0.1)	0.96 (0.95, 0.97)	0.17 (0.13, 0.21)	0.74 (0.72, 0.76)	0.69 (0.62, 0.75)	0.71 (0.66, 0.76)	0.71 (0.66, 0.75)	0.20 (0.18, 0.23)	0.96 (0.95, 0.96)	0.40 (0.36, 0.43)
Stacking ensemble	0.64 (0.62, 0.66)	0.70 (0.47, 0.77)	0.53 (0.47, 0.75)	0.54 (0.49, 0.73)	0.09 (0.08, 0.11)	0.96 (0.96, 0.97)	0.23 (0.19, 0.27)	0.71 (0.69, 0.73)	0.74 (0.65, 0.85)	0.59 (0.45, 0.68)	0.60 (0.49, 0.68)	0.16 (0.14, 0.18)	0.95 (0.95, 0.97)	0.33 (0.29, 0.36)
Logistic regression	0.70 (0.68, 0.73)	0.60 (0.45, 0.79)	0.71 (0.51, 0.83)	0.70 (0.52, 0.81)	0.12 (0.09, 0.16)	0.97 (0.96, 0.98)	0.30 (0.26, 0.35)	0.74 (0.72, 0.76)	0.69 (0.57, 0.76)	0.69 (0.62, 0.80)	0.69 (0.63, 0.77)	0.19 (0.17, 0.23)	0.95 (0.94, 0.96)	0.37 (0.34, 0.41)

ANN, artificial neural networks. AUC, area under the ROC curve; Sen., Sensitivity; Spe., Specificity; Acc., Accuracy; PPV, Positive predictive value; NPV, Negative predictive value.

To further assess model generalizability, we evaluated the optimal models using a more stringent SGA definition in which a newborn was classified as SGA only when identified as such by all four birthweight standards (*n* = 846; Figure 2B). Under this overlapping criterion, performance was comparable across standards, with mean AUCs of 0.741, 0.741, 0.729, and 0.721 for models based on the China, Intergrowth-21st, GROW, and FMF standards, respectively (Supplementary Figures S11A–D). However, bootstrap analysis revealed important differences in model performance: the FMF standard model demonstrated markedly superior discriminative ability, achieving a mean AUC of 0.981 (95% CI: 0.978–0.985). This substantially exceeded the performance of models based on the China (mean AUC: 0.749), Intergrowth-21st (0.753), and GROW (0.739) standards under their respective original definitions. The performance advantage of the FMF-based model was consistent across multiple metrics, including sensitivity (0.923 vs. 0.715–0.764), specificity (0.928 vs. 0.612–0.679), and accuracy (0.927 vs. 0.617–0.680) (Supplementary Figure S11E). The statistical superiority of the FMF-based model was further confirmed by DeLong tests, which revealed significant differences in ROC-AUC between the FMF standard model and all other models (all *p* < 0.001), while no significant difference was observed between the China and INTERGROWTH-21st standards (*p* = 0.739) (Supplementary Table S10).

### Model interpretation

**Figure 4:**
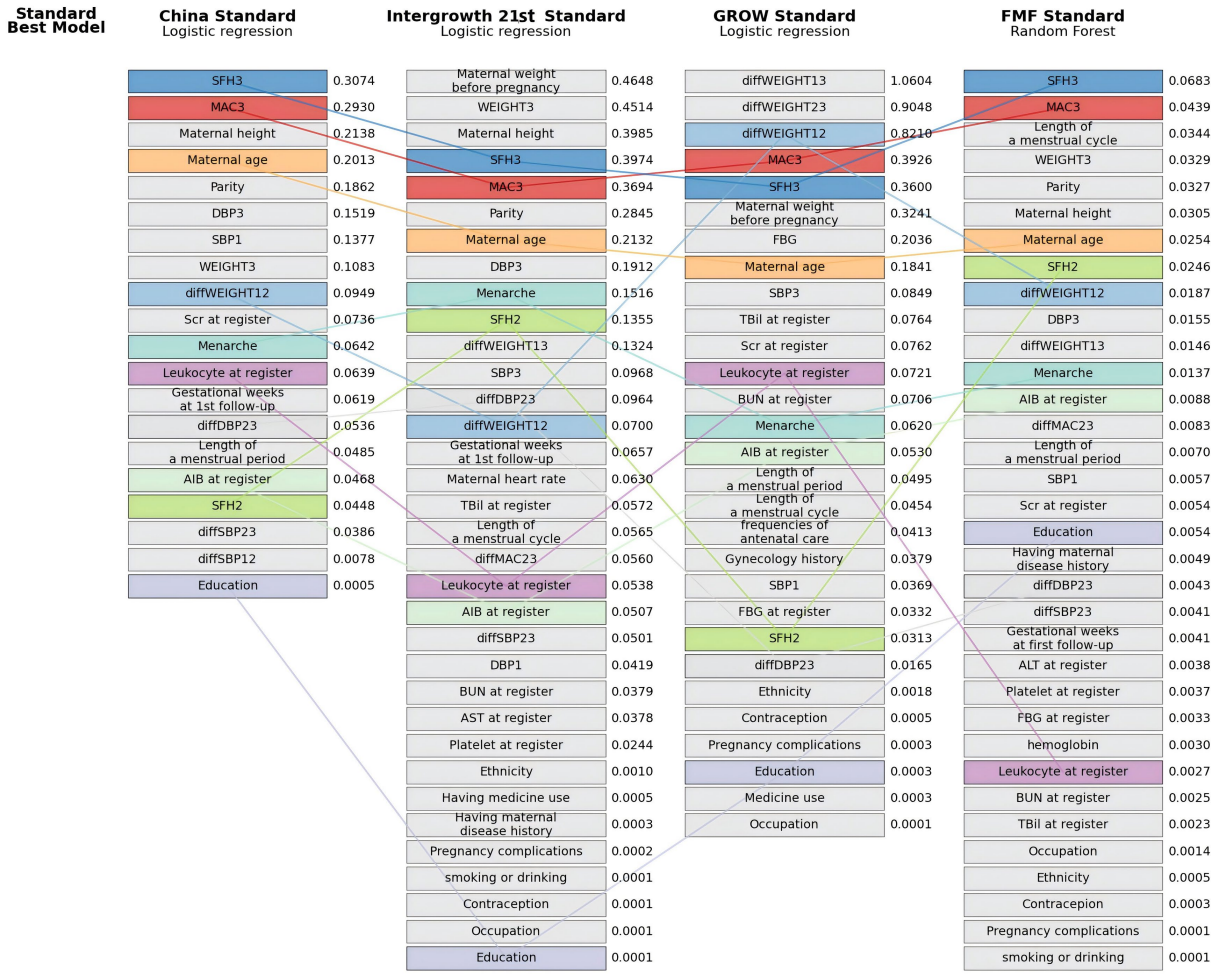
Predictor importance ranking for the optimal models across the four birthweight standards using the testing set of the complete data. Feature importance is ranked vertically by the mean absolute SHAP value, with the specific value labeled to the right of each bar. The China, Intergrowth-21st, and GROW standards used logistic regression as the optimal model, while the FMF standard used random forest. Common predictors across standards are connected by solid lines of the same color to facilitate comparison of rankings. AlB, albumin; ALT, alanine aminotransferase; AST, aspartate aminotransferase; BUN, serum urea nitrogen; DBP, diastolic blood pressure; FBG, fasting blood glucose; MAC, maternal abdominal circumference; Scr, serum creatinine; SBP, systolic blood pressure; SFH, symphysis fundal height; SGA, small for gestational age (birthweight <10th centile for gestational age); SHAP, Shapley Additive Explanations; TBil, total bilirubin. 1st pregnancy interval is the period before 18 gestational weeks. 2th pregnancy interval is the period between 18 and 25^+6^ gestational weeks. 3rd pregnancy interval is the period between 26 and 36^+6^ gestational weeks.

Variable importance, ranked by the mean absolute SHAP value for the best-performing model under each birthweight standard, is presented in Figure 4. The analysis identified consistent key predictors across the standards. Late-pregnancy symphysis-fundal height (SFH3) was the most important predictor for the China and FMF standards, and ranked fourth and fifth for the Intergrowth-21st and GROW standards, respectively. Similarly, late-pregnancy maternal abdominal circumference (MAC3) was the second-ranked predictor for the China and FMF standards and ranked within the top five for the other two standards. Maternal age was also identified as a highly influential variable, ranking within the top eight predictors for all standards. Furthermore, maternal height and weight, and parity were among the most important predictors for the China, Intergrowth-21st, and FMF standards. The connected lines in the figure visually demonstrate the variation in the relative ranking of these key predictors across the different standards. Based on the mean SHAP values from the best-fitting models for each birthweight standard, older maternal age was consistently associated with an increased risk of SGA, as indicated by positive mean SHAP values across all four standards. In contrast, features including SFH3, MAC3, maternal height and weight, and parity showed inconsistent directional associations with SGA risk, with positive influences under some standards and negative under others (Supplementary Table S11; Supplementary Figure S12). Based on the analysis of the imputed dataset, the ranking of predictor importance was largely consistent with that observed in the complete dataset, with late-pregnancy SFH, MAC, maternal age, height and weight, and parity remaining among the most influential features across the four standards (Supplementary Figure S13). The direction of association for key predictors, as indicated by the mean SHAP values, also showed patterns similar to those in the complete data (Supplementary Table S12; Supplementary Figure S14).

## Discussion

The SGA rate among Chinese newborns based on the China standard was 6.1%, which was similar to the GROW standard (6.0%), higher than the Intergrowth 21st standard (4.3%) and lower than the FMF standard (9.7%). Late pregnancy models had the best power to predict SGA, compared to middle and early pregnancy models, which is likely due to the additional relevant features/predictors that become available during the course of pregnancy, such as additional MAC and SFH. Our analysis revealed that optimal model performance was standard-dependent: logistic regression achieved the best performance for the China standard (ROC-AUC 0.74, PR-AUC 0.16), while Random Forest demonstrated superior performance for the FMF standard (ROC-AUC 0.79, PR-AUC 0.29, with sensitivity of 0.78, PPV of 0.20 and F1 score of 0.45). Symphysis fundal height, maternal abdominal circumference, maternal age, maternal height and weight, and parity were identified as key predictors of SGA.

In our study, the FMF standard classified the highest proportion of newborns as SGA, in line with two previous publications (Kabiri et al., 2020; Savirón-Cornudella et al., 2021). This elevated SGA rate can be attributed to the standard’s methodology, which integrates term birth data with estimated preterm birthweights based on the assumption that estimated fetal weight and birthweight share the same median across gestational ages (Nicolaides et al., 2018). Since preterm births are often associated with pathological conditions and fetal growth restriction, the FMF standard tends to classify more preterm infants as SGA (Nicolaides et al., 2018). The customized GROW standard classified an intermediate proportion of infants as SGA in our Chinese data set compared to population-based standards. This contrasts with reports from high-income countries, where the GROW standard typically identifies the highest proportion of SGA at birth (Odibo et al., 2018; Fernández-Alba et al., 2022; Zhang et al., 2007). The GROW standard used was constructed with China selected as the country of origin, resulting in SGA proportions similar to those identified by the China standard. Moreover, we observed substantial overlap in SGA classification between the Intergrowth 21st and China standards. This may partly be explained by their similar study designs as both were developed using data from low-risk, well-nourished women. Additionally, the Intergrowth 21st project included participants from Beijing, China, whose socioeconomic context aligns closely with that of population used to construct the China standard (Villar et al., 2014).

Our ML models have greatly improved predictive power for SGA, especially the Random forest-based model based on the FMF standard, compared to previous studies (Bai et al., 2022; Cho et al., 2022; Kuhle et al., 2018). A big data study comparing ML methods showed that a model using logistic regression with predictors available at 26 weeks appeared the best-fitting tool to predict SGA birth, with a ROC-AUC value of 0.66 for primiparous women (Kuhle et al., 2018). Our models developed at 26 weeks achieved better prediction, with an ROC-AUC of 0.70 based on the China standard and a ROC-AUC of 0.72 based on the FMF standard. Compared to the China standard, the superior performance of the Random Forest model with the FMF standard stems from its algorithmic advantage in handling complex data patterns, as it excels at capturing non-linear relationships and complex interactions among predictive features. This capability is critical for leveraging the nuanced information within the input variables, leading to more powerful discrimination for the specific task of SGA identification under any given standard (Couronné et al., 2018). In contrast, logistic regression remained the optimal or non-inferior model for the China, Intergrowth 21st, and GROW standards, suggesting predominantly linear predictor-outcome relationships, as supported by SHAP analysis. Furthermore, its structural simplicity mitigates overfitting and enhances generalizability (Christodoulou et al., 2019; Deo, 2015), while its inherent interpretability—providing transparent, quantifiable risk associations—offers a distinct advantage for potential clinical implementation (Rudin, 2019). The collective findings indicate that model superiority is context-dependent, hinging on a specific alignment between the model’s form and the prediction task’s requirements.

Our study demonstrates that the best-fitting models show potential for predicting SGA at birth, with performance varying by gestational age and birthweight standard. Specifically, the Random Forest model under the FMF standard achieved a ROC-AUC of 0.72 at 26 weeks of gestation, which represents a clinically promising performance for early risk stratification, and further improved to 0.79 in late pregnancy, which falls within the ‘acceptable’ to ‘excellent’ range according to common diagnostic benchmarks. Beyond prediction, our analysis also identified several key clinical predictors, including symphysis-fundal height, maternal abdominal circumference, maternal age, maternal height and weight, and parity, which may inform opportunities for individualized antenatal management. Within the framework of the Chinese tiered prenatal care system, this level of predictive capability at middle pregnancy could enable practical clinical triage by identifying high-risk pregnancies for intensified monitoring, such as through frequent serial symphysis-fundal height measurements and third-trimester ultrasound biometry, while maintaining standard care for lower-risk women, thereby optimizing resource allocation. These models thus provide a quantitative tool for improving SGA detection and management, while the identified key predictors further inform individualized antenatal management strategies.

An important next step will be the validation of our ML prediction models in independent data sets. Further improvements to our prediction models may be achieved by incorporating additional variables, such as previous pregnancy outcome details, glucose monitoring data, and ultrasound measurements. When validated and refined, ML prediction models need to be assessed prospectively, ideally in the context of an RCT, to ascertain improved prediction of SGA at birth. Ultimately, improved SGA prediction combined with interventions needs to be demonstrated to improve perinatal morbidity and mortality.

This study has several strengths. To our knowledge this is the first study to compare four population-based and customized birthweight standards using a large population-based data set, and develop ML models to predict SGA at birth at different stages of pregnancy. This is also the first study to compare the CatBoost method with widely used Random Forest, Stacked ensemble model, and ANN methods to determine the best-fitting model for each birthweight standard. Finally, our study is transparent in the methodology used for data processing, feature selection, prediction model development, assessment and interpretation, thereby reducing the potential for analytical bias.

This study has some limitations. While routine pregnancy surveillance data has the advantages of scale and inclusivity, it has the disadvantage of a relatively limited number of variables, which may have limited model performances. Second, the pregnancies in our study were from a single city in China, which may not be representative of all Chinese singleton pregnancies, which may limit the generalisability of the proposed models. Third, we assessed four representative birthweight standards. However, other standards, such as the World Health Organization Fetal Growth Charts and the NICHD fetal growth standard, assess antenatal fetal weight, relying on ultrasound scan rather than the newborn size, which may lead to more accurate estimation of SGA risk (Grantz et al., 2018; Kiserud et al., 2017). However, comparison of different fetal and birthweight standards showed that all standards assessed had poor performance for predicting adverse perinatal outcomes among an Australian population (Choi et al., 2021). Fourth, we did not compare the performance of the ML models to existing prediction methods that may currently be in use in Wenzhou and therefore cannot comment on ML performance relative to existing prediction methods. Finally, we did not have access to antenatal fetal ultrasound measurements, which might have improved our prediction models. However, routine access to antenatal ultrasound is not available in many LMICs, which have the highest burden of SGA and may benefit most from improved antenatal SGA prediction models.

In conclusion, this study reveals substantial variation in SGA classification across birthweight standards. Both sophisticated machine learning algorithms and conventional logistic regression demonstrated comparable predictive performance for SGA identification. These findings highlight the potential to enhance prenatal care through computational approaches that enable risk-stratified management.

## Data Availability

The data analyzed in this study is subject to the following licenses/restrictions: privacy and ethical restrictions. Requests to access these datasets should be directed to qiuyan_yu@wmu.edu.cn.
