# Supplementary material for: Antenatal prediction of small for gestational age at birth based on four birthweight standards using machine learning algorithms
Source: Front Artif Intell. 2026 Jan 12;8:1679979. doi: 10.3389/frai.2025.1679979 (PMC12832878; doi:10.3389/frai.2025.1679979)
Supplement: Supplementary file 1 [file Supplementary_file_1.pdf]

Title

Antenatal prediction of small for gestational age at birth with artificial intelligence: a modelling study based on four birthweight standards

Supplementary Figure S1. Selection process of participants.....3

Supplementary Figure S2. The overlap of non-SGA infants classified by four birthweight standards using the complete data set 4

Supplementary Figure S3. Feature selection by lasso regression in three pregnancy intervals for the China standard.....5

Supplementary Figure S4. Feature selection by lasso regression in three pregnancy intervals for the Intergrowth 21st standard.....6

Supplementary Figure S5. Feature selection by lasso regression in three pregnancy intervals for the GROW standard. 7

Supplementary Figure S6. Feature selection by lasso regression in three pregnancy intervals for the FMF standard. 8

Supplementary Figure S7. Receiver operating characteristic and precision-recall curves for prediction of small for gestational age at three pregnancy intervals in the testing set of imputed data according to four birthweight standards using seven prediction models.....9

Supplementary Figure S8. Calibration curves in the testing data set using the best-fitting model for each birthweight standard. 10

Supplementary Figure S9. Receiver operating characteristic curves of the optimal models on training and testing sets of the complete data for the four birthweight standards.11

Supplementary Figure S10. Receiver operating characteristic curves of the optimal models on training and testing sets of the imputed data for the four birthweight standards.12

Supplementary Figure S11. Comprehensive comparison of the best-fitting model of each birthweight standard on Performance, calibration, and feature importance based on the complete data set that SGAs were defined as the overlapping SGA classification of cases by the four birthweight standards. 13

Supplementary Figure S12. SHAP summary plot of the best-fitting model of each birthweight standard using the testing set of the complete data.....14

Supplementary Figure S13. Predictor importance ranking for the optimal models across the four birthweight standards using the testing set of imputed data. 15

Supplementary Figure S14. SHAP summary plot of the best-fitting model of each birthweight standard using the testing set of the imputed data. 16

Supplementary Table S1. Percentiles of gestational weeks at the registration visit.....17

Supplementary Table S2. List of maternal features recorded in pregnancy surveillance data. 18

Supplementary Table S3. The 53 features retained for predictive modelling. 20

Supplementary Table S4. Hyper-parameter spaces for random search for each base model in imputed data and complete data for the four birthweight standards. 21

Supplementary Table S5. Comparison of variable distribution before and after multiple imputation among 225,523 singleton pregnancies. 22

Supplementary Table S6. Feature selection from the candidate predictors available in each pregnancy intervals for each standard in imputed data and complete data. 24

Supplementary Table S7. Hyper-parameters of each prediction model using the complete data for the four birthweight standards. 26

Supplementary Table S8. Bootstrap Validation of prediction model performance using testing data set from the imputed data.. 30

Supplementary Table S9. Hyper-parameters of each prediction model using the imputed data for the four birthweight standards. 32

Supplementary Table S10. Delong test between the best-fitting model of each birthweight standard using the data with SGA defined as all of the four birthweight standards..36

Supplementary Table S11. Mean SHAP value of each feature for four birthweight standards in the complete data. 37

Supplementary Table S12. Mean SHAP value of each feature for four birthweight standards in the imputed data. 39

Supplementary Table S13 The comparison of delivery characteristics between the included pregnancies and excluded pregnancies. 40

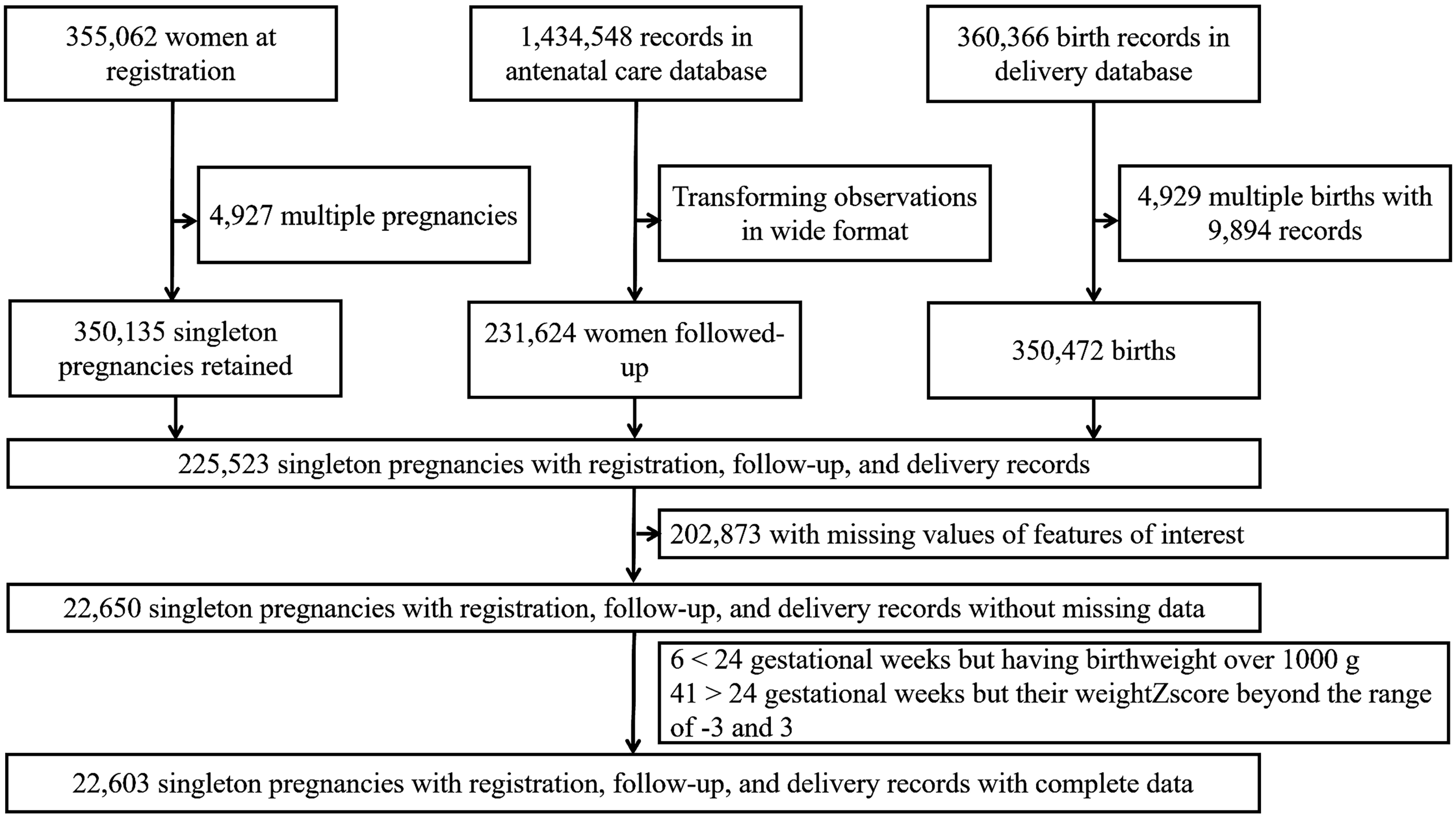

Supplementary Figure S1. Selection process of participants.

41 pregnancies > 24 gestational weeks but their weight Zscore beyond the range of -3 and 3 where the Z score was calculated according to Intergrowth21 standard.

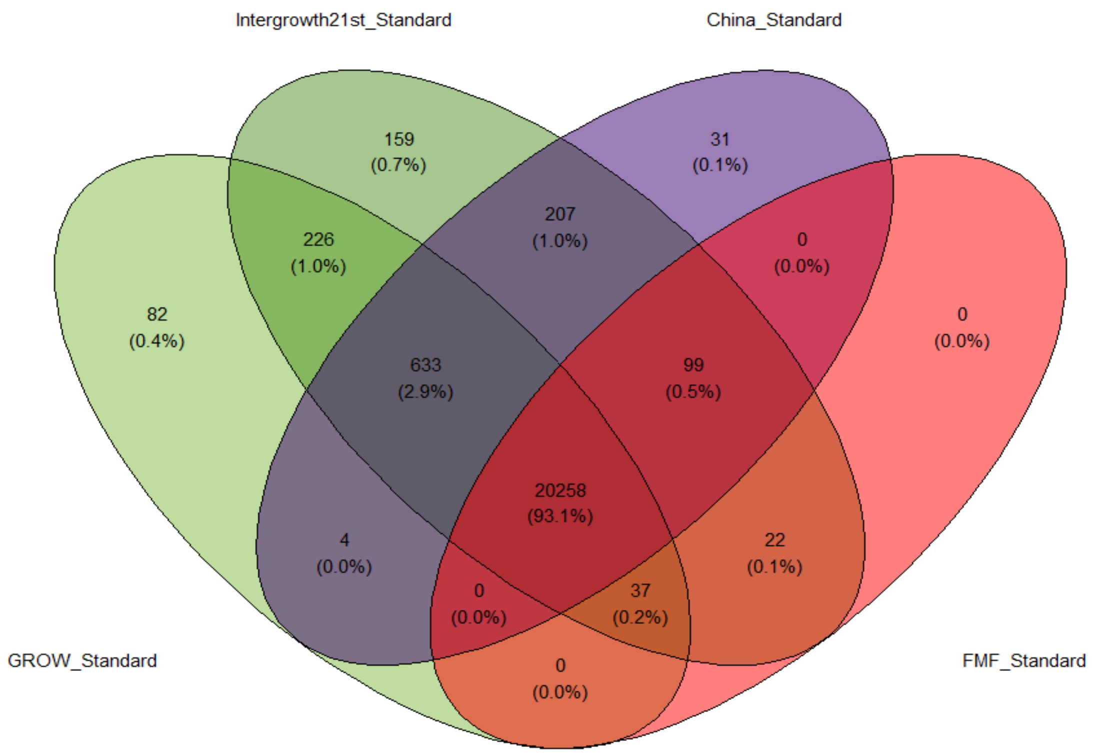

Supplementary Figure S2. The overlap of non-SGA infants classified by four birthweight standards using the complete data set .

This four-way Venn diagram illustrates the concordance and discordance among the INTERGROWTH-21st, China, GROW, and FMF birthweight standards in classifying non-SGA infants.Each circle represents the total number of infants classified as non-SGA by a specific standard.The overlapping areas represent infants who were concurrently classified as non-SGA by two, three, or all four standards.The number in each segment indicates the count of infants, with the corresponding percentage of the total population in parentheses.

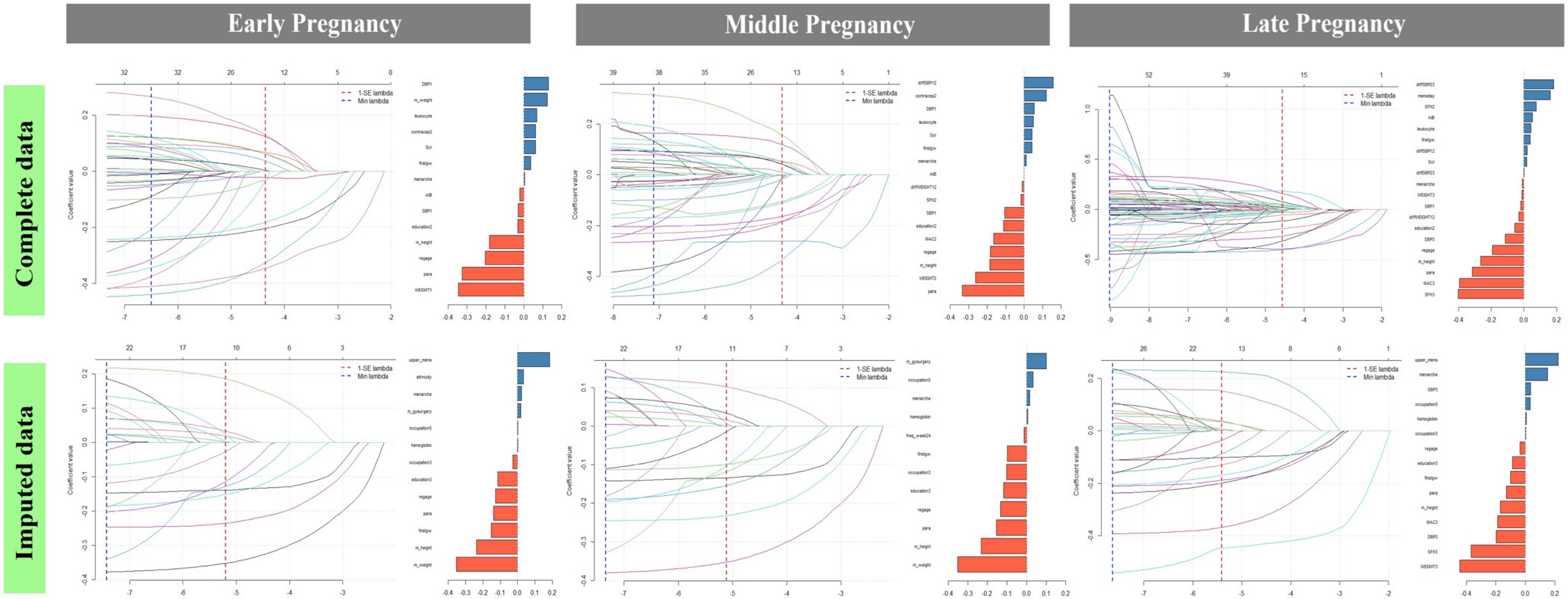

Supplementary Figure S3. Feature selection by lasso regression in three pregnancy intervals for the China standard.

This figure illustrates the feature selection process for the early, middle, and late pregnancy models. For each trimester, the results are presented for both the complete case data (top row) and the imputed data (bottom row). For all sub-figures, left panels are the lasso coefficient profiles that each line traces the coefficient path of a feature as the regularization penalty (lambda) increases. Red vertical dotted line represents the value of lambda chosen by one standard error rule (lambda.1se), which selects the most parsimonious model within one standard error of the minimum cross-validated error. Blue vertical dotted line represents the value of lambda that gives the minimum mean cross-validated error (lambda.min). This model typically has the best predictive performance; Right panels are variable Importance Rankings. This bar plot displays the top features selected at the lambda.1se value, ranked by the absolute value of their standardized coefficients.Bars on the RIGHT side represent features with positive coefficients, sorted by the absolute value of their coefficient in descending order, and bars on the LEFT side represent features with negative coefficients, sorted by the absolute value of their coefficient in ascending order

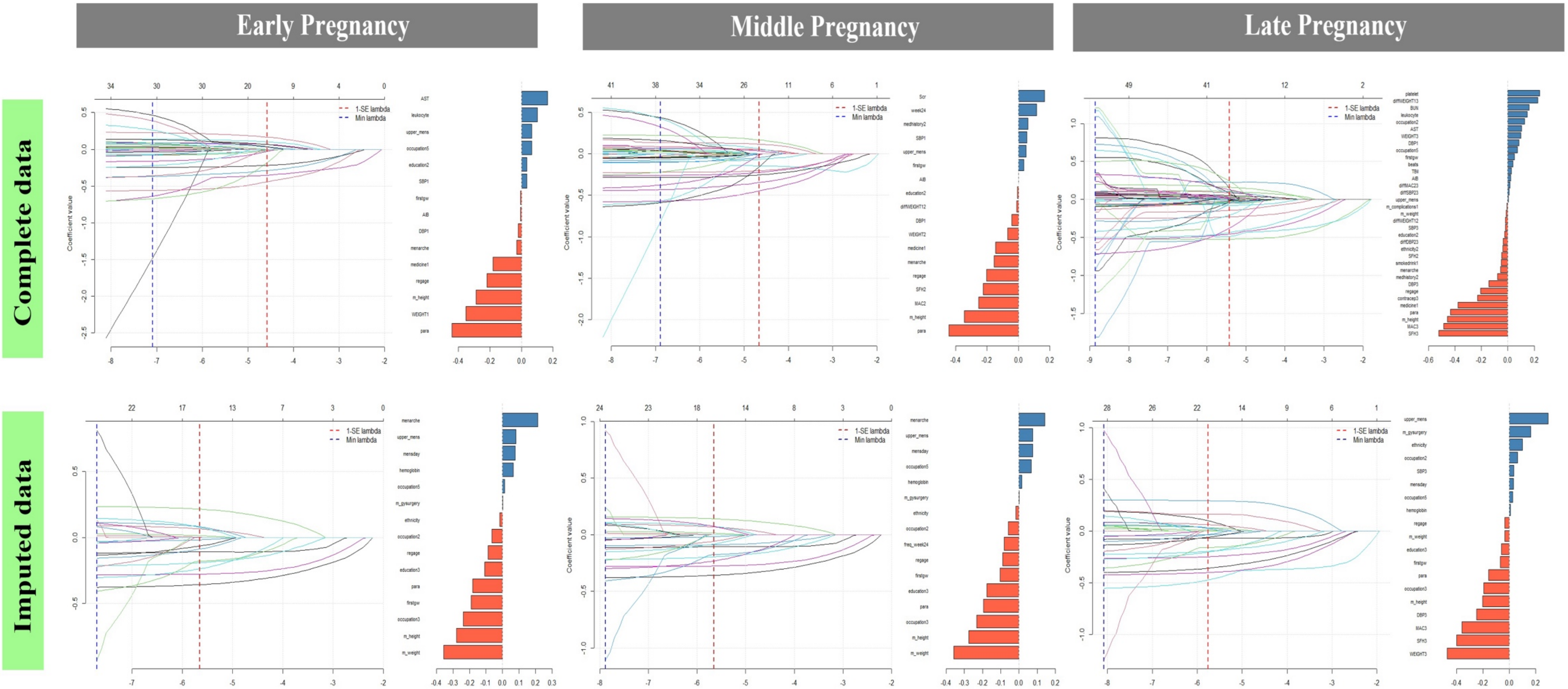

Supplementary Figure S4. Feature selection by lasso regression in three pregnancy intervals for the Intergrowth 21st standard.

This figure illustrates the feature selection process for the early, middle, and late pregnancy models. For each trimester, the results are presented for both the complete case data (top row) and the imputed data (bottom row). For all sub-figures, left panels are the lasso coefficient profiles that each line traces the coefficient path of a feature as the regularization penalty (lambda) increases. Red vertical dotted line represents the value of lambda chosen by one standard error rule (lambda.1se), which selects the most parsimonious model within one standard error of the minimum cross-validated error. Blue vertical dotted line represents the value of lambda that gives the minimum mean cross-validated error (lambda.min). This model typically has the best predictive performance; Right panels are variable Importance Rankings. This bar plot displays the top features selected at the lambda.1se value, ranked by the absolute value of their standardized coefficients.Bars on the RIGHT side represent features with positive coefficients, sorted by the absolute value of their coefficient in descending order, and bars on the LEFT side represent features with negative coefficients, sorted by the absolute value of their coefficient in ascending order.

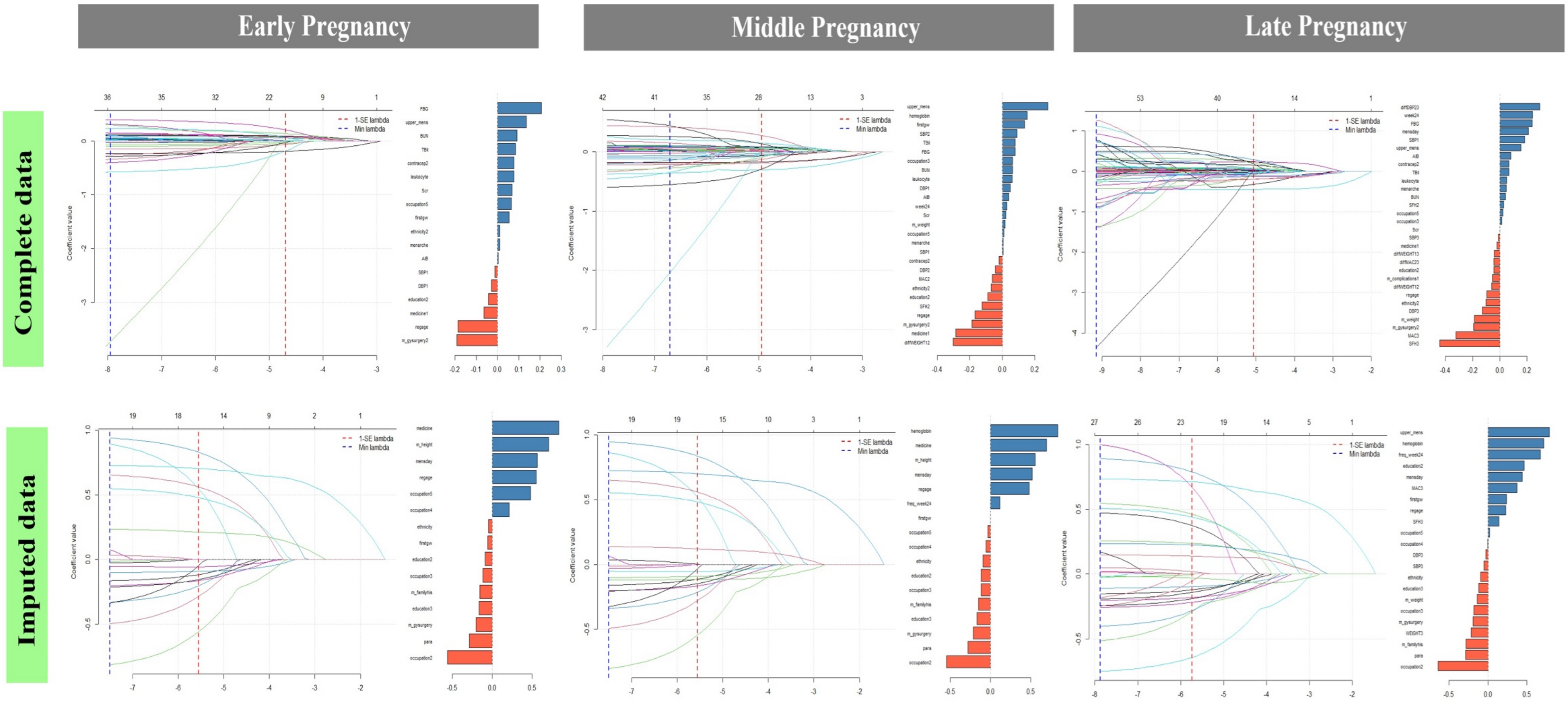

Supplementary Figure S5. Feature selection by lasso regression in three pregnancy intervals for the GROW standard.

This figure illustrates the feature selection process for the early, middle, and late pregnancy models. For each trimester, the results are presented for both the complete case data (top row) and the imputed data (bottom row). For all sub-figures, left panels are the lasso coefficient profiles that each line traces the coefficient path of a feature as the regularization penalty (lambda) increases. Red vertical dotted line represents the value of lambda chosen by one standard error rule (lambda.1se), which selects the most parsimonious model within one standard error of the minimum cross-validated error. Blue vertical dotted line represents the value of lambda that gives the minimum mean cross-validated error (lambda.min). This model typically has the best predictive performance; Right panels are variable Importance Rankings. This bar plot displays the top features selected at the lambda.1se value, ranked by the absolute value of their standardized coefficients.Bars on the RIGHT side represent features with positive coefficients, sorted by the absolute value of their coefficient in descending order, and bars on the LEFT side represent features with negative coefficients, sorted by the absolute value of their coefficient in ascending order.

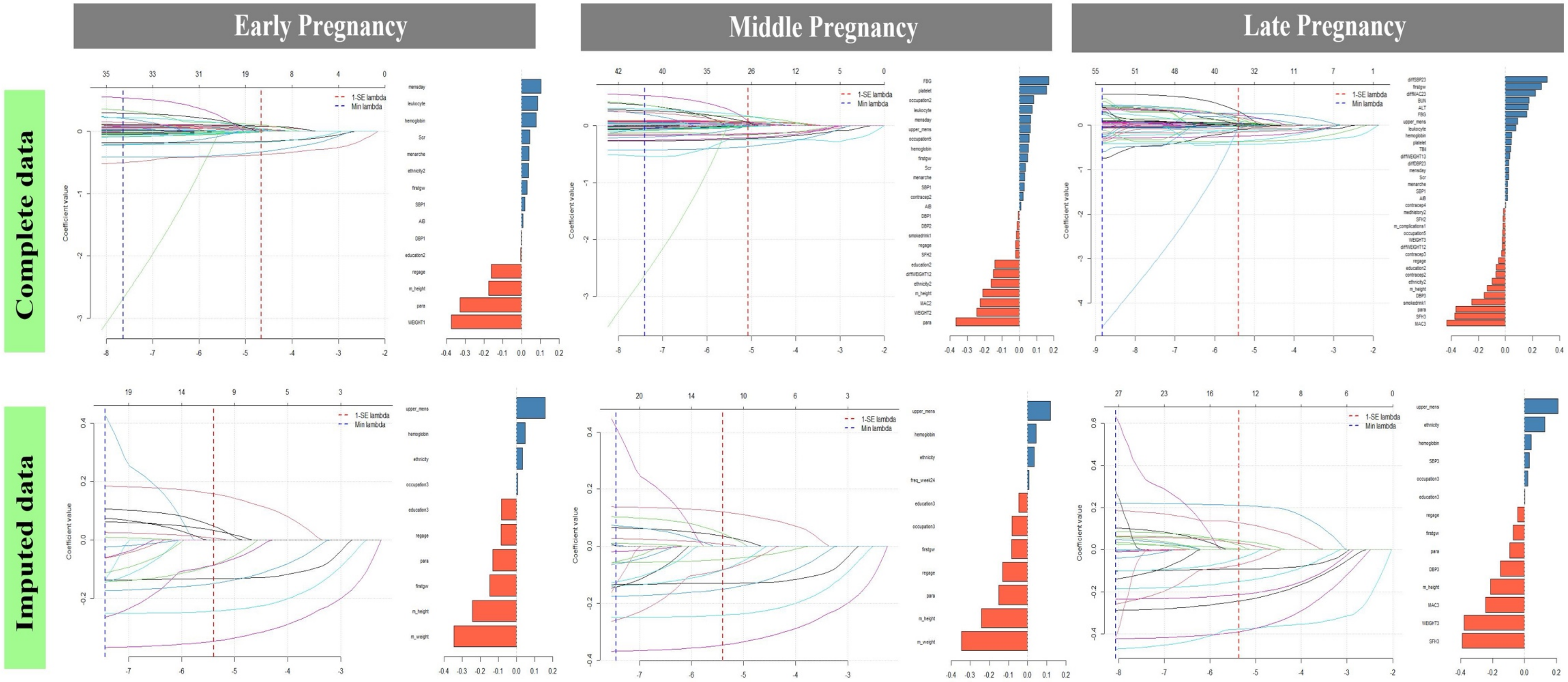

Supplementary Figure S6. Feature selection by lasso regression in three pregnancy intervals for the FMF standard.

This figure illustrates the feature selection process for the early, middle, and late pregnancy models. For each trimester, the results are presented for both the complete case data (top row) and the imputed data (bottom row). For all sub-figures, left panels are the lasso coefficient profiles that each line traces the coefficient path of a feature as the regularization penalty (lambda) increases. Red vertical dotted line represents the value of lambda chosen by one standard error rule (lambda.1se), which selects the most parsimonious model within one standard error of the minimum cross-validated error. Blue vertical dotted line represents the value of lambda that gives the minimum mean cross-validated error (lambda.min). This model typically has the best predictive performance; Right panels are variable Importance Rankings. This bar plot displays the top features selected at the lambda.1se value, ranked by the absolute value of their standardized coefficients. Bars on the RIGHT side represent features with positive coefficients, sorted by the absolute value of their coefficient in descending order, and bars on the LEFT side represent features with negative coefficients, sorted by the absolute value of their coefficient in ascending order.

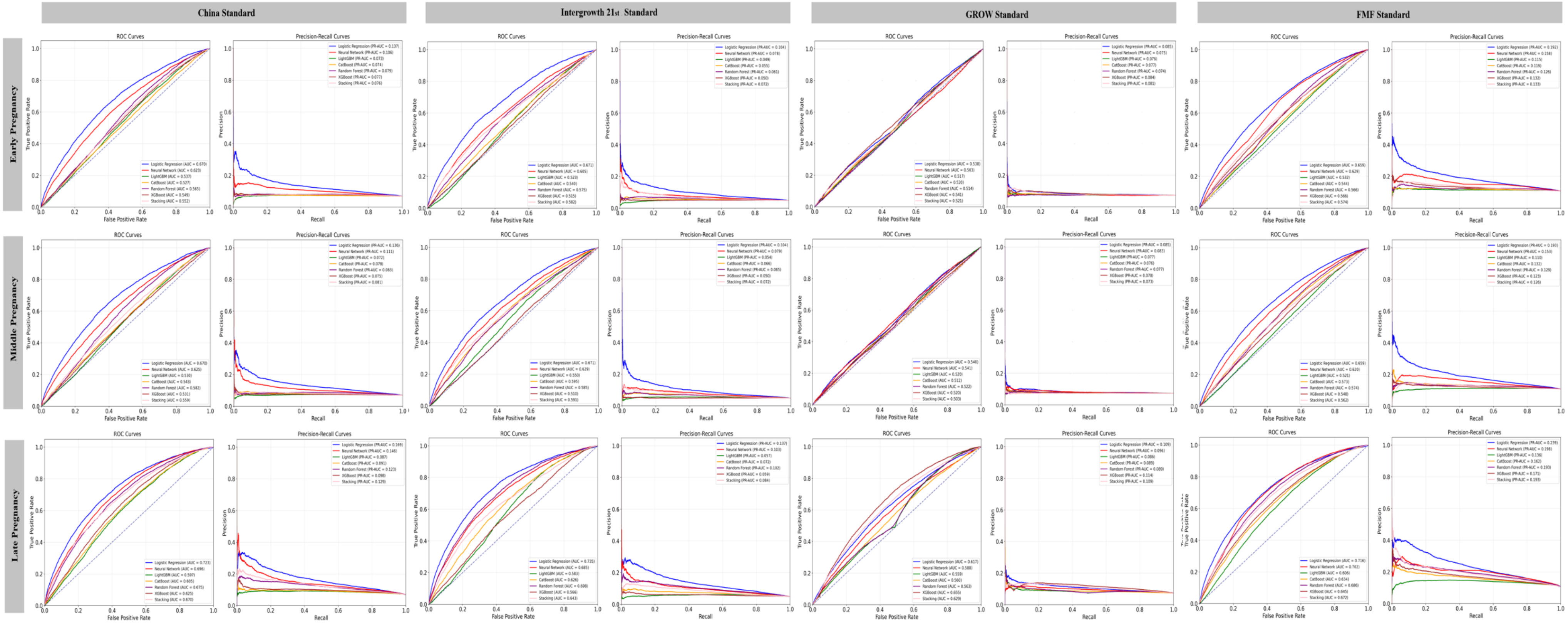

Supplementary Figure S7. Receiver operating characteristic and precision-recall curves for prediction of small for gestational age at three pregnancy intervals in the testing set of imputed data according to four birthweight standards using seven prediction models.

The evaluation is stratified into three distinct pregnancy intervals. For each interval, the predictive performance is assessed according to four different birthweight standards (China, INTERGROWTH-21st, GROW, and FMF). Each sub-panel displays the results of seven different prediction models, allowing for a direct comparison of their performance. Left Column: ROC Curves that The Area Under the Curve (AUC) quantifies the model's overall ability to discriminate between SGA and non-SGA infants, irrespective of class distribution. A higher AUC indicates better performance, with 1.0 representing perfect discrimination and 0.5 representing a random guess. Right Column: Precision-Recall Curves that The Area Under the PR Curve (PR-AUC) is a more informative metric than AUC for imbalanced datasets, where SGA cases are typically the minority class. A higher PR-AUC indicates better performance, with a baseline representing the proportion of SGA infants in the data set.

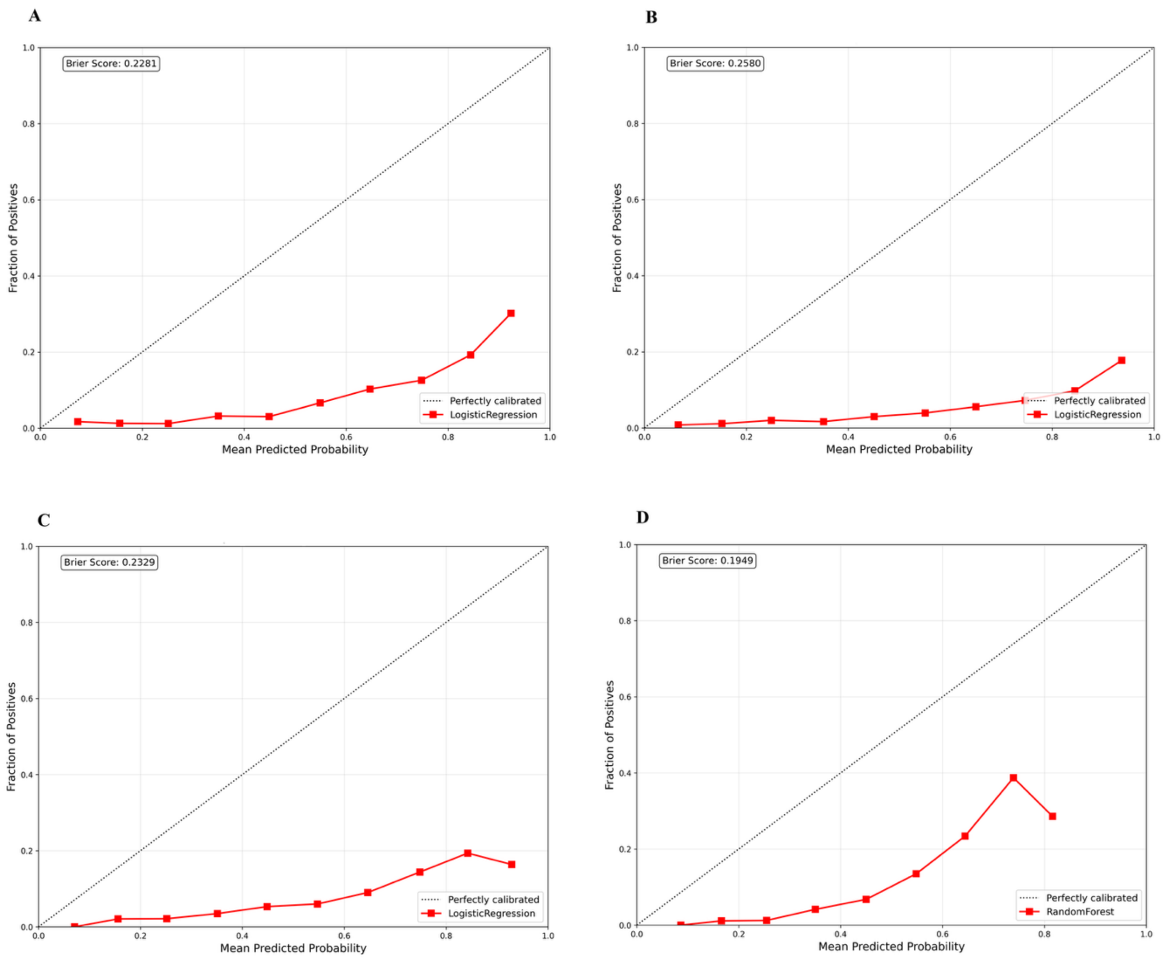

Supplementary Figure S8. Calibration curves in the testing data set using the best-fitting model for each birthweight standard.

(A)The best-fitting model for the China standard based on the Logistic regression. (B)The best-fitting model for the Intergrowth21st standard based on the Logistic regression. (C)The best-fitting model for the GROW standard based on the Logistic Regression. (D) The best-fitting model for the FMF standard based on the Random Forest algorithm. GROW = Gestation-related Optimal Weight, FMF = Fetal Medicine Foundation, SVM = Support Vector Machine.

The solid diagonal line represents the ideal scenario where the model's predicted probability of an infant being SGA perfectly matches the observed proportion in the data.The red solid line represents the actual calibration performance of the fitted model. The closer the red line is to the diagonal dashed line, the better the model is calibrated. The Brier Score is the mean squared difference between the predicted probability and the actual outcome (0 or 1). A lower Brier Score indicates better overall performance, with a value of 0 representing perfect accuracy.

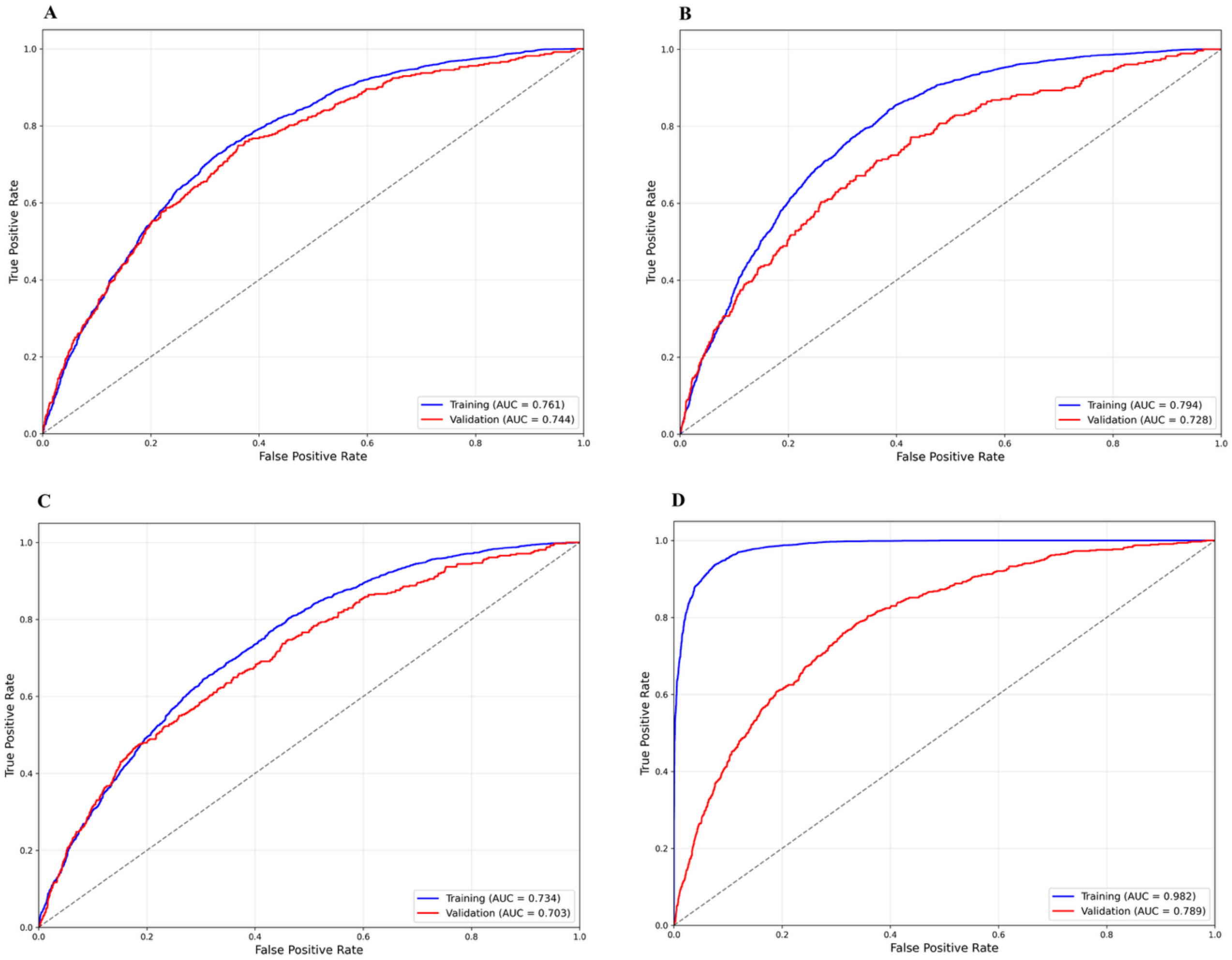

Supplementary Figure S9. Receiver operating characteristic curves of the optimal models on training and testing sets of the complete data for the four birthweight standards. (A) China standard, (B) INTERGROWTH-21st standard, (C) GROW standard, (D) FMF standard

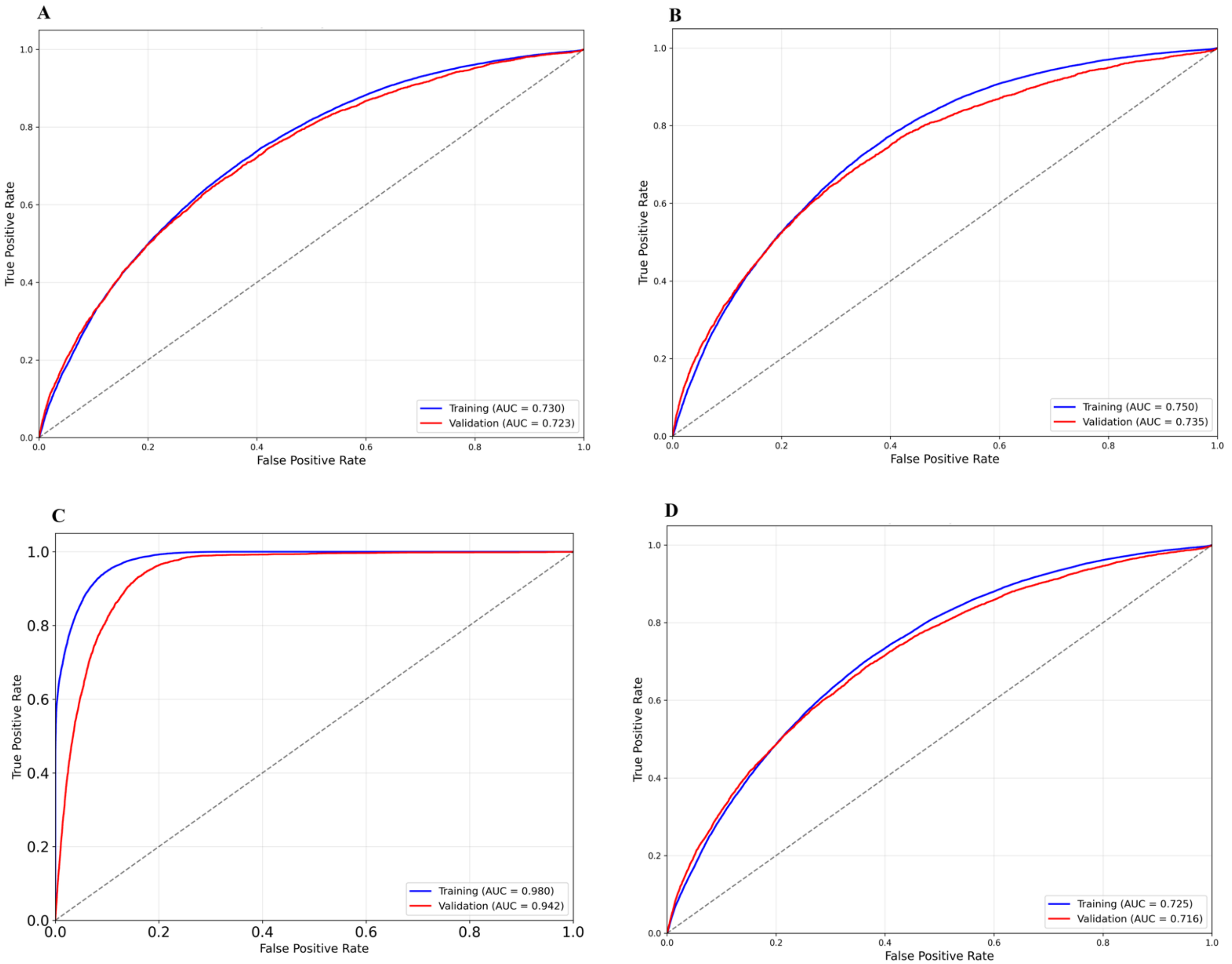

Supplementary Figure S10. Receiver operating characteristic curves of the optimal models on training and testing sets of the imputed data for the four birthweight standards. (A) China standard, (B) INTERGROWTH-21st standard, (C) GROW standard, (D) FMF standard.

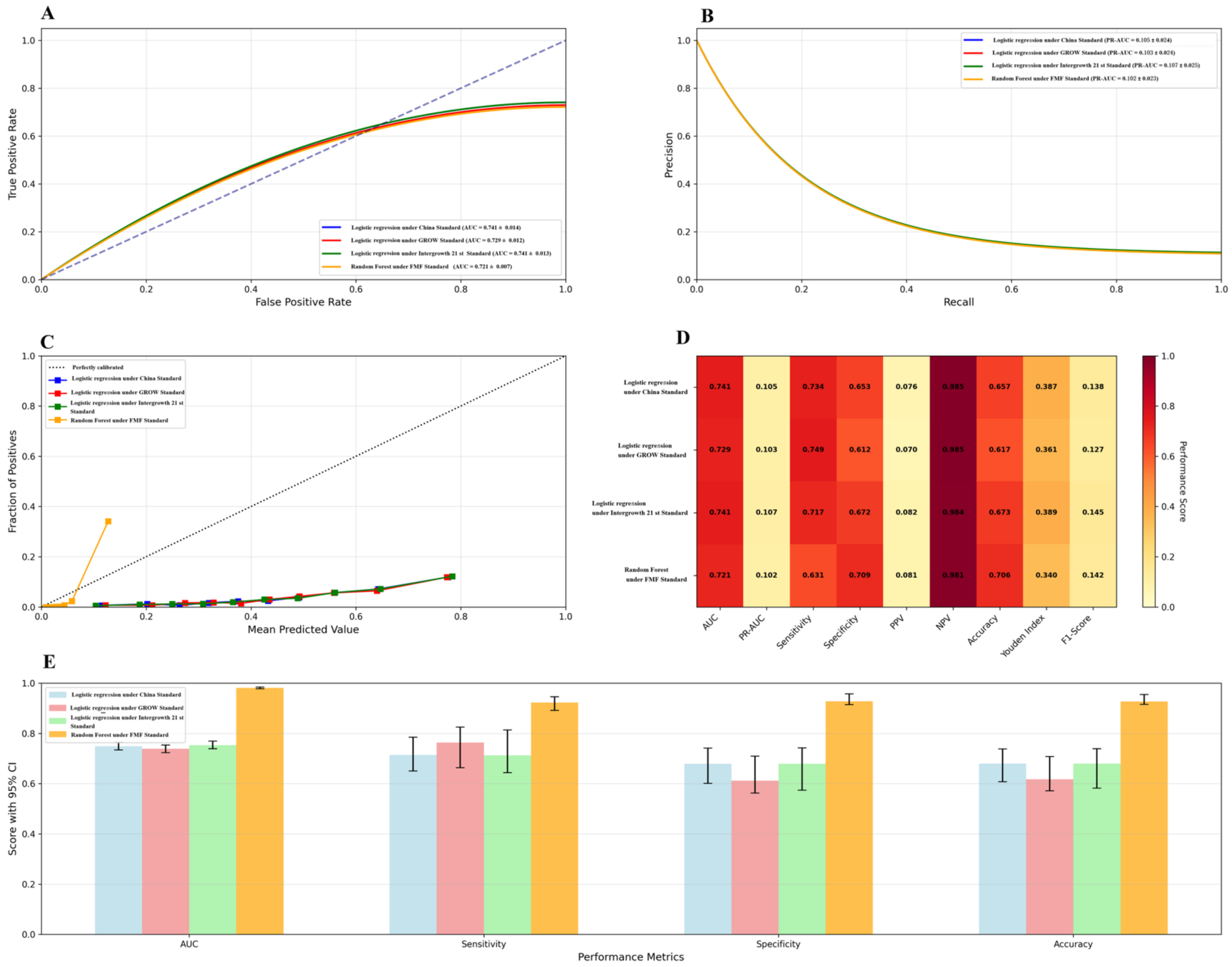

Supplementary Figure S11. Comprehensive comparison of the best-fitting model of each birthweight standard on Performance, calibration, and feature importance based on the complete data set that SGAs were defined as the overlapping SGA classification of cases by the four birthweight standards.

(A) ROC curve comparison, (B) Precision-Recall curve comparison, (C) Calibration curve comparison, (D) Performance matrics heatmap. (E) bootstrap confidence interval comparison

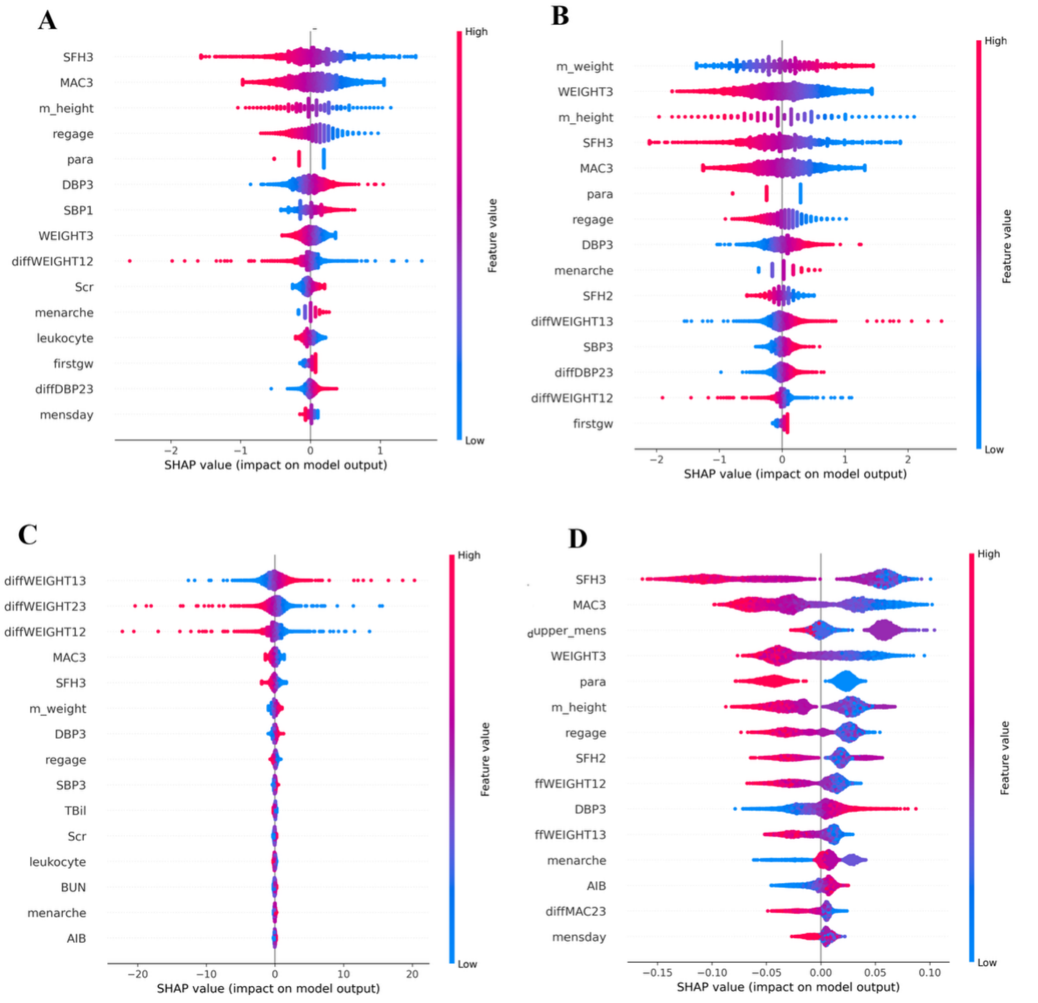

Supplementary Figure S12. SHAP summary plot of the best-fitting model of each birthweight standard using the testing set of the complete data.

(A) China standard, (B) INTERGROWTH-21st standard, (C) GROW standard, (D) FMF standard.

SHAP = Shapley Additive Explanations.Features are listed on the y-axis, ranked from top to bottom by their global importance of the mean absolute value of their SHAP values across all instances). Each point represents the SHAP value for a single prediction of a single individual in the test set. The x-axis shows the SHAP value, which represents the impact of a feature on the model's output for that specific prediction. A positive SHAP value increases the predicted birthweight, while a negative SHAP value decreases it.The color of each point indicates the feature value for that individual, from low (blue) to high (red), scaled to a standard normal distribution.

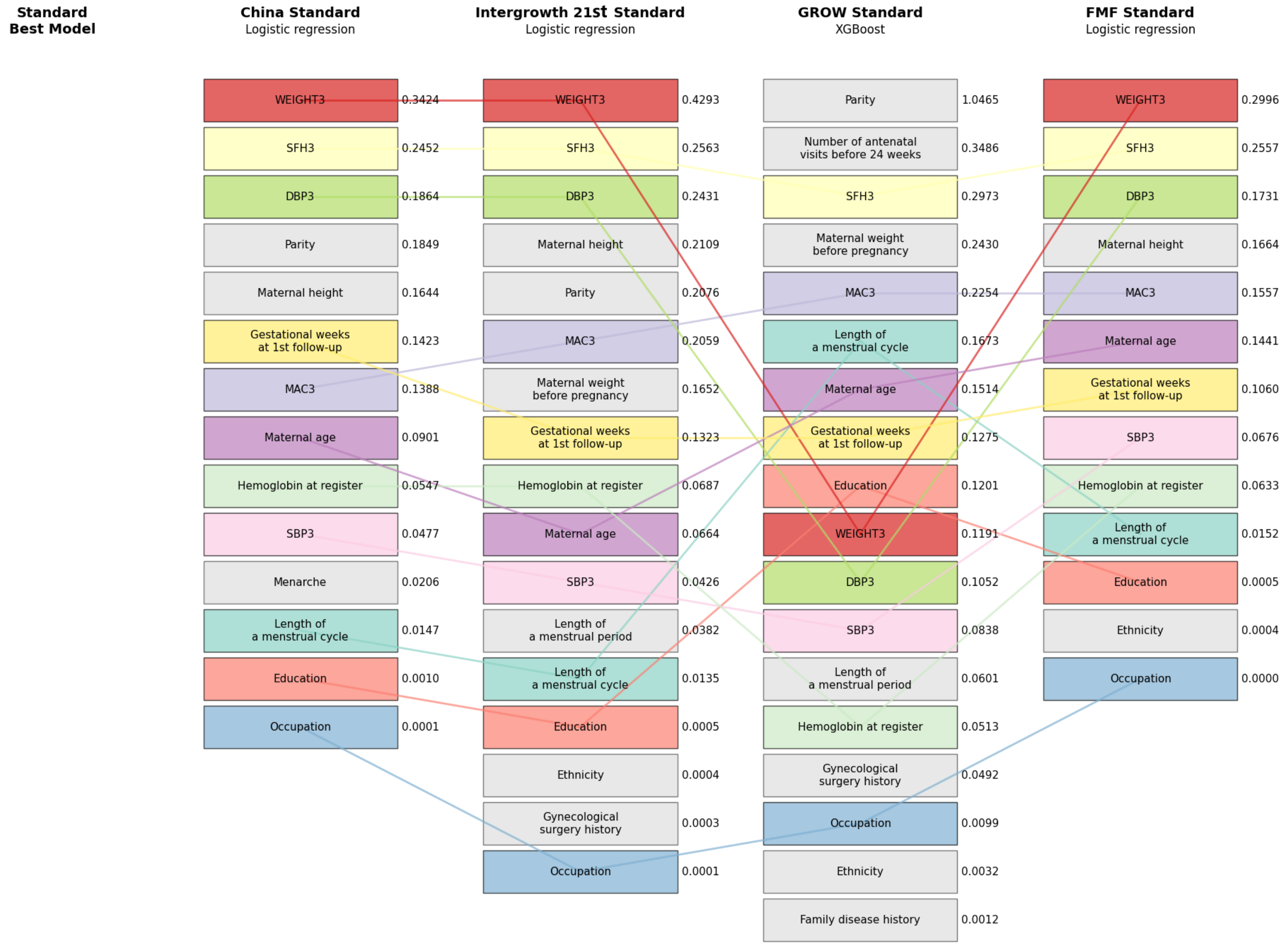

Supplementary Figure S13. Predictor importance ranking for the optimal models across the four birthweight standards using the testing set of imputed data. Feature importance is ranked vertically by the mean absolute SHAP value, with the specific value labeled to the right of each bar. China, Intergrowth 21st, and FMF standards used logistic regression as the optimal model, and GROW standard used XGBoost as the optimal model. Common predictors across standards are connected by solid lines of the same color to facilitate comparison of ranking differences.

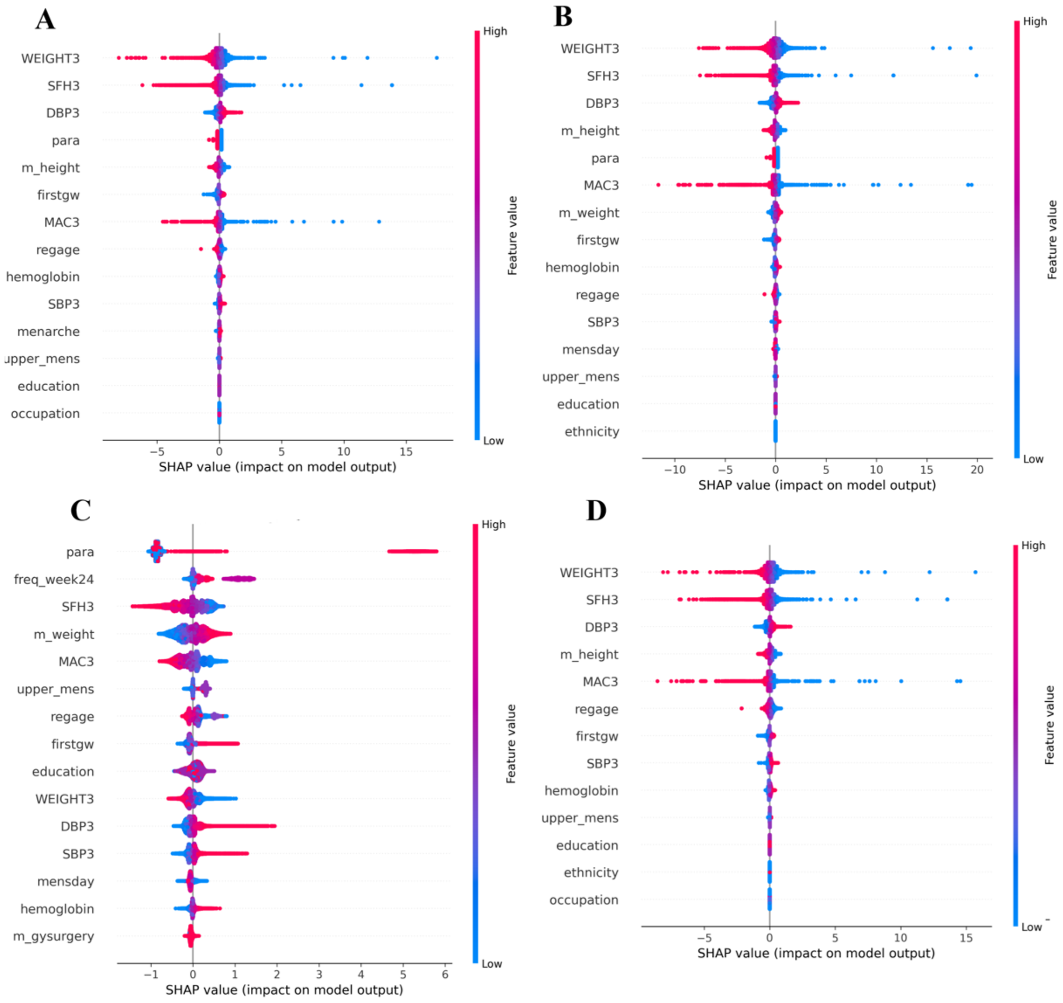

Supplementary Figure S14. SHAP summary plot of the best-fitting model of each birthweight standard using the testing set of the imputed data.

(A) China standard, (B) INTERGROWTH-21st standard, (C) GROW standard, (D) FMF standard.

SHAP = Shapley Additive Explanations.Features are listed on the y-axis, ranked from top to bottom by their global importance of the mean absolute value of their SHAP values across all instances). Each point represents the SHAP value for a single prediction of a single individual in the test set. The x-axis shows the SHAP value, which represents the impact of a feature on the model's output for that specific prediction. A positive SHAP value increases the predicted birthweight, while a negative SHAP value decreases it.The color of each point indicates the feature value for that individual, from low (blue) to high (red), scaled to a standard normal distribution

Supplementary Table S1. Percentiles of gestational weeks at the registration visit.

Year	Percentiles, weeks						
	5	10	25	50	75	90	95
2014	11.00	13.00	16.00	18.00	25.00	33.00	36.00
2015	9.00	12.00	14.00	17.00	23.00	32.00	35.00
2016	10.00	12.00	14.00	17.00	22.00	31.00	35.00

Supplementary Table S2. List of maternal features recorded in pregnancy surveillance data.

F.code	F.name	F.type	F.source
regage	Maternal age at registration, y	Numeric	Registration data
week24	Number of antenatal visits before 24 weeks	Numeric	Follow-up data
firstgw	Ultrasound gestational weeks at 1st visit	Numeric	Follow-up data
gwage	Ultrasound corrected gestational age, weeks	Numeric	Delivery records
m_weight	Maternal weight, kg	Numeric	Registration data
m_height	Maternal height, cm	Numeric	Registration data
menarche	Age of menarche, y	Numeric	Registration data
upper_mens	Length of a menstrual cycle, days	Numeric	Registration data
mensday	Length of a menstrual period, days	Numeric	Registration data
para	Parity	Numeric	Registration data
beats	Maternal heart rate, times per minute	Numeric	Registration data
Hemoglobin	Hemoglobin, g/L	Numeric	Registration data
Leukocyte	White blood cell count, 10^9/L	Numeric	Registration data
Platelet	Platelet count, 10^9/L	Numeric	Registration data
FBG	Fasting blood glucose, mmol/L	Numeric	Registration data
ALT	Alanine aminotransferase, U/L	Numeric	Registration data
AST	Aspartate aminotransferase, U/L	Numeric	Registration data
AIB	Albumin, g/L	Numeric	Registration data
TBil	Total bilirubin, μmol/L	Numeric	Registration data
Scr	Serum creatinine, μmol/L	Numeric	Registration data
BUN	Serum urea nitrogen, mmol/L	Numeric	Registration data
Occupation	Occupation	Nominal	Registration data
Education	Education	Nominal	Registration data
Ethnicity	Ethnicity	Binary	Registration data
smokealcohol	Maternal smoking/alcohol use	Binary	Registration data
medhistory	Maternal disease history	Binary	Registration data
medicine	Medicine use	Binary	Registration data
m_gysurgery	Gynecological surgery history	Binary	Registration data
contracep	Contraception history	Binary	Registration data

Pregnancy complications	Clinical diagnosis with previa, eclampsia, or pregnancy-induced hypertension	Binary	Delivery data
SBP1	SBP1[#], mmHg	Numeric	Follow-up data
SBP2	SBP2[##], mmHg	Numeric	Follow-up data
SBP3	SBP3[###], mmHg	Numeric	Follow-up data
DBP1	DBP1[#], mmHg	Numeric	Follow-up data
DBP2	DBP2[##], mmHg	Numeric	Follow-up data
DBP3	DBP3[###], mmHg	Numeric	Follow-up data
Weight1	Weight1[#], kg	Numeric	Follow-up data
Weight2	Weight2[##], kg	Numeric	Follow-up data
Weight3	Weight3[###], kg	Numeric	Follow-up data
SFH1	SFH1[##], cm	Numeric	Follow-up data
SFH2	SFH2[##], cm	Numeric	Follow-up data
SFH3	SFH3[###], cm	Numeric	Follow-up data
MAC1	MAC1[##], cm	Numeric	Follow-up data
MAC2	MAC2[##], cm	Numeric	Follow-up data
MAC3	MAC3[###], cm	Numeric	Follow-up data
birthweight	Birthweight was measured within 1 hour of birth, g	Numeric	Delivery data
Neo_sex	Neonatal sex	Binary	Delivery data

1st pregnancy interval is the period before 17^{+6} gestational weeks.

2th pregnancy interval is the period between 18 and 25^{+6} gestational weeks.

3rd pregnancy interval is the period between 26 and 36^{+6} gestational weeks.

Abbreviations: AIB = Albumin, ALT= Alanine aminotransferase, AST = Aspartate aminotransferase. BUN = Serum urea nitrogen, beats = maternal heart rate, DBP = diastolic blood pressure, FBG = fasting blood glucose, MAC = maternal abdominal circumference, Maternal complications = clinical diagnosis with previa, eclampsia, or pregnancy-induced hypertension. SBP = systolic blood pressure, Scr = Serum creatinine, SGA = small for gestational age (birthweight <10th centile for gestational age), TBil = Total bilirubin, upper_mens = Length of a menstrual cycle, days, week24 = number of antenatal visits before 24 gestational weeks..

Supplementary Table S3. The 53 features retained for predictive modelling.

Data resource	F.code
Registration data (26 items)	regage, m_weight, m_height, menarche, upper_mens, mensday, para, beats, Hemoglobin, Leukocyte, Platelet, FBG, ALT, AST, AIB, TBil, Scr, BUN, Occupation, Education, Ethnicity, smokealcohol, medhistory, medicine, m_gysurgery, contracep.
Follow up data (26 items)	Week24, firstgw,<br>SBP1, SBP2, SBP3, DBP1, DBP2, DBP3, Weight1,Weight2, Weight3, SFH2, SFH3, MAC2, MAC3;<br>diffSBP12, diffSBP23, diffSBP13, diffDBP12, diffDBP23, diffDBP13, diffWEIGHT12, diffWEIGHT13, diffWEIGHT23, diffSFH23, diffMAC23;
Delivery data (1 items)	Pregnancy complications

The explanation of F.code refer to Supplementary Table S2.

Supplementary Table S4. Hyper-parameter spaces for random search for each base model in imputed data and complete data for the four birthweight standards.

Models	Hyper-parameter space
LightBoost	'num_boost_round': [100, 200, 300, 500],<br>'learning_rate': [0.01, 0.05, 0.1, 0.15, 0.2],<br>'num_leaves': [31, 63, 127, 255],<br>'min_data_in_leaf': [20, 50, 100, 200],<br>'feature_fraction': [0.6, 0.7, 0.8, 0.9, 1.0],<br>'bagging_fraction': [0.6, 0.7, 0.8, 0.9, 1.0],<br>'bagging_freq': [0, 1, 5, 10],<br>'reg_alpha': [0, 0.1, 0.5, 1.0],<br>'reg_lambda': [0, 0.1, 0.5, 1.0]
CatBoost	'iterations': [100, 200, 300, 500],<br>'learning_rate': [0.01, 0.05, 0.1, 0.15, 0.2],<br>'depth': [4, 6, 8, 10],<br>'l2_leaf_reg': [1, 3, 5, 7, 9],<br>'random_strength': [0, 0.5, 1.0, 2.0],<br>'bagging_temperature': [0, 0.5, 1.0],<br>'leaf_estimation_iterations': [1, 5, 10]
Random Forest	'n_estimators': [100, 200, 300, 500],<br>'max_depth': [5, 10, 15, 20, None],<br>'min_samples_split': [2, 5, 10, 20],<br>'min_samples_leaf': [1, 2, 4, 8],<br>'max_features': ['auto', 'sqrt', 'log2', 0.5, 0.7, 0.9],<br>'bootstrap': [True, False]
Artificial Neural Networks	'hidden_layer_sizes': [(64, 32), (128, 64), (64,), (128,), (256, 128), (32, 16, 8), (128, 64, 32), (256, 128, 64)],<br>'activation': ['relu', 'tanh', 'logistic'],<br>'alpha': [0.0001, 0.0005, 0.001, 0.005, 0.01, 0.05],<br>'learning_rate_init': [0.0001, 0.001, 0.01, 0.1],<br>'batch_size': [16, 32, 64, 128],<br>'solver': ['adam', 'sgd'],<br>'beta_1': [0.8, 0.9, 0.95],<br>'beta_2': [0.99, 0.999, 0.9999]
Logistic regression	'C': [0.001, 0.01, 0.1, 1, 10, 100, 1000],<br>'penalty': ['l1', 'l2', 'elasticnet'],<br>'solver': ['liblinear', 'saga', 'lbfgs'],<br>'max_iter': [1000, 2000, 3000],<br>'class_weight': [None, 'balanced']

The format of hyper-parameter spaces for each model represents as python codes.

Supplementary Table S5. Comparison of variable distribution before and after multiple imputation among 225,523 singleton pregnancies.

Characteristic	Data with MI	Data without MI	*P* value	SMD
Neonatal sex as boys	120514 (53.9)	120519 (53.9)	1.000	<0.001
Neonatal weight, g	3,300.00 [3,050, 3,600]	3,300 [3,050, 3,600]	1.000	<0.001
Neonatal length, cm	50.00 [50.00, 50.00]	50.00 [50.00, 50.00]	0.997	<0.001
Maternal age at register, yr	26.00 [23.00, 30.00]	26.00 [23.00, 30.00]	1.000	<0.001
Occupation (%)			1.000	<0.001
Farmer or fishermen	123613 (55.3)	123613 (55.3)		
Employee	13395 (6.0)	13395 (6.0)		
Business and service industry	17239 (7.7)	17239 (7.7)		
Households	44765 (20.0)	44765 (20.0)		
Others	24580 (11.0)	24580 (11.0)		
Education			0.832	0.002
Primary school and below	12202 (5.5)	12261 (5.5)		
Secondary school and high school	154967 (69.7)	155593 (69.6)		
College and above	55279 (24.9)	55738 (24.9)		
Han ethnicity	213949 (95.6)	213589 (95.5)	0.425	0.002
No Disease history	221699 (99.2)	221699 (99.2)	1.000	<0.001
Having family history	1185 (0.5)	1185 (0.5)	1.000	<0.001
No congenital disease	223592 (100.0)	223592 (100.0)	-	<0.001
Ever smoking	206 (0.1)	206 (0.1)	1.000	<0.001
Ever drinking	268 (0.1)	268 (0.1)	1.000	<0.001
Ever smoking or drinking	389 (0.2)	389 (0.2)	1.000	<0.001
Medicine use during pregnancy	938 (0.4)	938 (0.4)	1.000	<0.001
No Gynecology surgery history	216205 (96.7)	216205 (96.7)	1.000	<0.001
Age of menarche,yr	14.00 [13.00, 15.00]	14.00 [13.00, 15.00]	0.956	<0.001
Upper days for a menstrual cycle	30.00 [28.00, 30.00]	30.00 [28.00, 30.00]	0.230	0.004
Days for a menstrual period	5.00 [4.00, 6.00]	5.00 [4.00, 6.00]	0.741	<0.001
Contraception			-	<0.001
Never	219611 (98.2)	219611 (98.2)		
Physical contraception	3235 (1.4)	3235 (1.4)		
Chemical contraception	690 (0.3)	690 (0.3)		
Both	56 (0.0)	56 (0.0)		
Parity	0.00 [0.00, 1.00]	0.00 [0.00, 1.00]	0.834	0.001
Maternal height at register	159.00 [156.00, 162.00]	159.00 [156.00, 162.00]	0.873	<0.001
Maternal weight at register	53.00 [49.00, 59.00]	53.30 [49.00, 59.00]	0.044	0.007
Hemoglobin at register	122.00 [115.00, 129.00]	122.00 [114.00, 129.00]	<0.001	0.015
SBP3#	113.43 [107.00, 120.00]	113.33 [106.80, 120.00]	0.019	0.007
DBP3#	68.50 [63.67, 73.20]	68.50 [63.67, 73.25]	0.254	0.003
WEIGHT3#	62.62 [57.70, 68.20]	62.62 [57.67, 68.21]	0.603	<0.001
SFH3#	30.00 [28.50, 31.40]	30.00 [28.40, 31.33]	<0.001	0.018
MAC3#	92.00 [88.00, 96.00]	92.00 [88.00, 96.00]	0.001	0.002
Frequency of prenatal visit before delivery	6.00 [3.00, 9.00]	6.00 [3.00, 9.00]	1.000	<0.001
Gestational weeks at register	17.00 [14.00, 23.00]	17.00 [14.00, 23.00]	1.000	<0.001
Number of antenatal visits before 24 weeks	2.00 [1.00, 3.00]	2.00 [1.00, 3.00]	1.000	<0.001

Numeric variables represent as median[First Quartile, Third Quartile], and Mann-Whitney U test was to compare distribution of Numeric variable.

Categorical variables represent as n(%), and Chi-square test was used to compare proportion of Categorical variable.

3rd pregnancy interval is the period between 26 and 36+6 gestational weeks.

Abbreviations:MI, multiple imputation; SMD,Standardized Mean Difference;SBP, Systolic blood pressure; DBP, diastolic blood pressure; MAC, maternal abdominal circumference;SFH, Symphysis fundal height; WEIGHT, maternal weight during antenatal visit;

Supplementary Table S6. Feature selection from the candidate predictors available in each pregnancy intervals for each standard in imputed data and complete data.

Pregnancy intervals	Datasets	Candidate predictors available	Predictors identified by Lasso regression			
			China Standard	Intergrowth 21th standard	FMF standard	GROW standard
Early pregnancy	Imputed data	n = 19<br>[1] "regage" "menarche" "upper_mens" "mensday" "para"<br>[6] "m_height" "m_weight" "hemoglobin" "firstgw" "m_gysurgery"<br>[11] "occupation" "education" "ethnicity" "medhistory" "m_familyhis"<br>[16] "m_smoke" "m_drink" "smokedrink" "medicine"	n = 11, lambda = 0.0061<br>upper_mens，ethnicity，menarche， m_gysurgery，occupation，hemoglobin，education，regage，para，firstgw，m_height，m_weight	n = 15, lambda = 0.0035<br>menarche, upper_mens, mensday, hemoglobin, occupation, m_gysugery, ethnicity, regage, education, para, firstgw, m_height, m_weight	n = 10, lambda = 0.0045<br>upper_mens, hemoglobin, ethnicity,occupation, education,regage, para, firstgw, m_height, m_weight	n = 15, lambda = 0.0039<br>medcine,m_height,mensday, regage, occupation, ethnicity, firstgw, education, m_familyhis,m_gysurgey, para
	Complete data	n = 30<br>[1] "regage" "menarche" "upper_mens" "mensday" "m_height"<br>[6] "m_weight" "beats" "hemoglobin" "leukocyte" "platelet"<br>[11] "FBG" "ALT" "AST" "AIB" "TBil"<br>[16] "Scr" "BUN" "SBP1" "DBP1" "WEIGHT2"<br>[21] "firstgw" "m_gysurgery "para" "occupation" "education"<br>[26] "ethnicity" "smokedrink" "contracep" "medhistory" "medicine"	n = 14, lambda = 0.013<br>DBP1,m_weight, leukocyte, contraception, Scr, firstgw, menarche, AIB, SBP1, education, m_height, regage, para, Weight1	n = 15, lambda = 0.010<br>AST, leukocyte, upper_mens,occupation, education, SBP1, firstgw, AIB, DBP1, menarche, medicine use, regage, m_height,WEIGHT1, para	n =15, lambda = 0.0095<br>mensday, leukocyte, Scr, menarche, ethnicity, firstgw, SBP1, AIB, DBP1, education, regage, m_height, para, WEIGHT1	n = 18, lambda = 0.0092<br>FBG, upper_mens, BUN, TBil, contraception, leukocyte, Scr, occupation, firstgw, ethnicity, menarche, AIB, SBP1, DBP1, education, medicine, regage, m_gysurgery
Middle pregnancy	Imputed data	n = 20<br>[1] "regage" "menarche" "upper_mens" "mensday" "para"<br>[6] "m_height" "m_weight" "hemoglobin" "firstgw" "freq_week24"<br>[11] "m_gysurgery" "occupation" "education" "ethnicity" "medhistory"<br>[16] "m_familyhis" "m_smoke" "m_drink" "smokedrink" "medicine"	n = 12, lambda = 0.0061<br>m_gysurgery,occupation,menarche, hemoglobin, week24, firstgw,education, regage,para, m_height,m_weight	n = 16, lambda = 0.0035<br>menarche, upper_mens, mensday,occupation, hemoglobin, m_gysurgery, ethnicity, week24, regage, firstgw, education, para, m_height, m_weight	n = 11,lambda = 0.0045<br>upper_mens, hemoglobin, ethnicity, week24, education, occupation, firstgw, regage, para, m_height, m_weight	n = 17, lambda = 0.0039<br>hemoglobin, medicine, m_height, mensday, regage, week24, firstgw, occupation, ethnicity, education, m_familyhis, m_gysurgery, para
	Complete data	n =39<br>[1] "week24" "regage" "menarche" "upper_mens" "mensday"<br>[6] "m_height" "m_weight" "beats" "hemoglobin" "leukocyte"<br>[11] "platelet" "FBG" "ALT" "AST" "AIB"<br>[16] "TBil" "Scr" "BUN" "SBP1" "SBP2"<br>[21] "DBP1" "DBP2" "WEIGHT1" "WEIGHT2" "SFH2"<br>[26] "MAC2" "firstgw" "diffSBP12" "diffDBP12" "diffWEIGHT12"<br>[31] "m_gysurgery" "para" "occupation" "education" "ethnicity"<br>[36] "smokedrink" "contracep" "medhistory" "medicine"	n = 17, lambda = 0.013<br>diffSBP12,contraception, DBP1, lekocyte, Scr, firstgw, menarche, AIB, diffWEIGHT12, SFH2, SBP1,education, MAC2, regage, m_height, WEIGHT2, para	n = 18, lambda = 0.0094<br>Scr, week24, medhistory, SBP1, upper_mens, firstgw, AIB, education, diffWEIGHT12, DBP1, WEIGHT2, medicine use, menarche, regage, SFH2, MAC2, m_height, para	n =26, lambda = 0.0062<br>FBG,platelet,occupation, leukocyte, mensday, upper_mens, hemoglobin, firstgw,Scr, menarche, SBP1, contraception, AIB, DBP1,DBP2, smokedrink, regage, SFH2, education, diffWEIGHT12, ethnicity, m_height, MAC2, WEIGHT2, para	n = 27, lambda = 0.0071<br>upper_mens, hemoglobin, firstgw, SBP2, TBil, FBG, occupation, BUN, leukocyte, DBP1, AIB, week24, Scr, m_weight, occupation, menarche, SBP1, contraception, DBP2, MAC2, ethnicity, education, SFH2, regage, m_gysurgery, medicine, diffWEIGHT12
Late pregnancy	Imputed data	n =25<br>[1] "regage" "menarche" "upper_mens" "mensday" "para"<br>[6] "m_height" "m_weight" "hemoglobin" "SBP3" "DBP3"<br>[11] "WEIGHT3" "SFH3" "MAC3" "firstgw" "freq_week24"<br>[16] "m_gysurgery" "occupation" "education" "ethnicity" "medhistory"<br>[21] "m_familyhis" "m_smoke" "m_drink" "smokedrink" "medicine"	n = 15, lambda = 0.0045<br>upper_mens, menarche, SBP3, occupation, hemoglobin, regage, education, firstgw, para, m_height, MAC3, DBP3, SFH3, WEIGHT3	n = 19, lambda = 0.0032<br>upper_mens, m_gysurgery, ethnicity, occupation, SBP3, mensday, hemoglobin, regage, m_weight, education, firsgw, para, m_height, DBP3, MAC3, SFH3, WEIGHT3	n = 14, lambda = 0.0047<br>upper_mens, hemoglobin, ehnicity, week24, education, occupation, firstgw, regage, para, m_height, m_weight	n = 22, lambda = 0.0032<br>upper_mens,hemoglobin, week24, education, mensday, MAC3, firstgw, regage, SFH3, occupation, DBP3,SBP3,ethnicity, m_weight, m_gysurgery, WEIGHT3, m_familyhis, para

Complete data	n =53<br>[1] "week24" "regage" "menarche" "upper_mens"<br>[5] "mensday" "m_height" "m_weight" "beats"<br>[9] "hemoglobin" "leukocyte" "platelet" "FBG"<br>[13] "ALT" "AST" "AIB" "TBil"<br>[17] "Scr" "BUN" "SBP1" "SBP2"<br>[21] "SBP3" "DBP1" "DBP2" "DBP3"<br>[25] "WEIGHT1" "WEIGHT2" "WEIGHT3" "SFH2"<br>[29] "SFH3" "MAC2" "MAC3" "firstgw"<br>[33] "diffSBP12" "diffSBP23" "diffSBP13" "diffDBP12"<br>[37] "diffDBP23" "diffDBP13" "diffWEIGHT12" "diffWEIGHT23"<br>[41] "diffWEIGHT13" "diffSFH23" "diffMAC23" "m_complications"<br>[45] "para" "occupation" "education" "m_gysurgery"<br>[49] "ethnicity" "smokedrink" "contracep" "medhistory"<br>[53] "medicine"	n = 20, lambda = 0.011<br>diffSBP12, mensday, SFH2, AIB, leukocyte, firstgw,diffSBP12, Scr, diffDBP23, menarche, WEIGHT3, SBP1,diffWEIGHT12, education, DBP3, regage, m_height, para, MAC3, SFH3	n = 35, lambda = 0.0044<br>platelet, diffWEIGHT13, BUN,leukocyte, occupation, AST, WEIGHT3, DBP1, firstgw,beats, TBil, AIB,diffMAC23, diffSBP23, upper_mens,m_complication, m_weight,diffWEIGHT12, SBP3,education, diffDBP23, ethnicity, SFH2, smokedrink1, menarche, medhistory, DBP3,regage, contraception,medicine, para, m_height, MAC3, SFH3	n = 36, lambda = 0.0045<br>diffSBP23, firstgw, diffMAC23, BUN, ALT, FBG, upper_mens, leukocyte, hemoglobin, platelet, TBil, diffWEIGHT13, diffDBP23, menday, Scr, menarche, SBP1, AIB, contracep, medhistory, SFH2, m_complications, occupation, WEIGHT3, diffWEIGHT12, regage, education, ethnicity, m_height, DBP3, smokedrink, para, SFH3, MAC3	n = 30, lambda = 0.0064<br>diffDBP23, week24, FBG, mensday, SBP1, upper_mens,AIB, contraception, TBil, leukocyte, menarche, BUN, SFH2, occupation, SCr, SBP3, medicine, diffWEIGHT13, diffWEIGHT23, education, m_complications, diffWEIGHT12, regage, ethnicity, DBP3, m_weight, m_gusugery, MAC3, SFH3

Supplementary Table S7. Hyper-parameters of each prediction model using the complete data for the four birthweight standards.

Pregnancy intervals	Models	Hyper-parameters used in prediction models			
		China Standard	Intergrowth 21th standard	GROW standard	FMF standard
Early pregnancy	Logistic Regression	solver = lbfgs<br>penalty = l2<br>max_iter = 1000<br>class_weight = balanced<br>C = 1000	solver = saga<br>penalty = l2<br>max_iter = 3000<br>class_weight = None<br>C = 100	solver = lbfgs<br>penalty = l2<br>max_iter = 1000<br>class_weight = None<br>C = 1000	solver = liblinear<br>penalty = l2<br>max_iter = 2000<br>class_weight = balanced<br>C = 0.01
	ANN	solver =adam<br>learning_rate_init = 0.01<br>hidden_layer_sizes = (50, 25)<br>batch_size = 32<br>alpha = 0.0001<br>activation = relu	solver =adam<br>learning_rate_init = 0.01<br>hidden_layer_sizes = (50, 25)<br>batch_size = 32<br>alpha = 0.0001<br>activation = relu	solver =adam<br>learning_rate_init = 0.01<br>hidden_layer_sizes = (50, 25)<br>batch_size = 32<br>alpha = 0.0001<br>activation = relu	solver =adam<br>learning_rate_init = 0.01<br>hidden_layer_sizes = (50, 25)<br>batch_size = 32<br>alpha = 0.0001<br>activation = relu
	LightGBM	subsample = 0.7<br>reg_lambda = 0.5<br>reg_alpha = 0.1<br>num_leaves = 63<br>n_estimators = 200<br>min_child_samples = 20<br>learning_rate = 0.05<br>colsample_bytree = 0.9	subsample = 0.7<br>reg_lambda = 0.5<br>reg_alpha = 0.1<br>num_leaves = 63<br>n_estimators = 200<br>min_child_samples = 20<br>learning_rate = 0.05<br>colsample_bytree = 0.9	subsample = 0.7<br>reg_lambda = 0.5<br>reg_alpha = 0.1<br>num_leaves = 63<br>n_estimators = 200<br>min_child_samples = 20<br>learning_rate = 0.05<br>colsample_bytree = 0.9	subsample = 0.7<br>reg_lambda = 0.5<br>reg_alpha = 0.1<br>num_leaves = 63<br>n_estimators = 200<br>min_child_samples = 20<br>learning_rate = 0.05<br>colsample_bytree = 0.9
	Random Forest	n_estimators = 300<br>min_samples_split = 2<br>min_samples_leaf = 1<br>max_samples = 0.8<br>max_features = sqrt<br>max_depth = 10<br>bootstrap = TRUE	n_estimators = 300<br>min_samples_split = 2<br>min_samples_leaf = 1<br>max_samples = 0.8<br>max_features = sqrt<br>max_depth = 10<br>bootstrap = TRUE	n_estimators = 300<br>min_samples_split = 2<br>min_samples_leaf = 1<br>max_samples = 0.8<br>max_features = sqrt<br>max_depth = 10<br>bootstrap = TRUE	n_estimators = 300<br>min_samples_split = 2<br>min_samples_leaf = 1<br>max_samples = 0.8<br>max_features = sqrt<br>max_depth = 10<br>bootstrap = TRUE
	CatBoost	random_strength = 1<br>learning_rate = 0.1<br>l2_leaf_reg = 3<br>iterations = 300<br>depth = 6	random_strength = 1<br>learning_rate = 0.1<br>l2_leaf_reg = 3<br>iterations = 300<br>depth = 6	random_strength = 1<br>learning_rate = 0.1<br>l2_leaf_reg = 3<br>iterations = 300<br>depth = 6	random_strength = 1<br>learning_rate = 0.1<br>l2_leaf_reg = 3<br>iterations = 300<br>depth = 6
	XGBoost	subsample = 0.7<br>reg_lambda = 0.1<br>reg_alpha = 0<br>n_estimators = 300<br>min_child_weight = 1<br>max_depth = 5<br>learning_rate = 0.05<br>gamma = 0.2<br>colsample_bytree = 0.8	subsample = 0.7<br>reg_lambda = 0.1<br>reg_alpha = 0<br>n_estimators = 300<br>min_child_weight = 1<br>max_depth = 5<br>learning_rate = 0.05<br>gamma = 0.2<br>colsample_bytree = 0.8	subsample = 0.7<br>reg_lambda = 0.1<br>reg_alpha = 0<br>n_estimators = 300<br>min_child_weight = 1<br>max_depth = 5<br>learning_rate = 0.05<br>gamma = 0.2<br>colsample_bytree = 0.8	subsample = 0.7<br>reg_lambda = 0.1<br>reg_alpha = 0<br>n_estimators = 300<br>min_child_weight = 1<br>max_depth = 5<br>learning_rate = 0.05<br>gamma = 0.2<br>colsample_bytree = 0.8
	Stacking ensemble	Base models: the above six optimal models	Base models: the above six optimal models<br>meta_learner =LogisticRegression	Base models: the above six optimal models	Base models: the above six optimal models<br>meta_learner =LogisticRegression

		meta_learner =LogisticRegression<br>(C=0.1, random_state=self.random_state,<br>max_iter=500)	(C=0.1, random_state=self.random_state,<br>max_iter=500)	meta_learner =LogisticRegression<br>(C=0.1,random_state=self.random_state,<br>max_iter=500)	(C=0.1, random_state=self.random_state,<br>max_iter=500)
Middle pregnancy	Logistic Regression	solver = liblinear<br>penalty = l2<br>max_iter = 2000<br>class_weight = balanced<br>C = 0.01	solver = saga<br>penalty = l2<br>max_iter = 3000<br>class_weight = balanced<br>C = 100	solver = liblinear<br>penalty = l1<br>max_iter = 2000<br>class_weight = balanced<br>C = 1000	solver = saga<br>penalty = l2<br>max_iter = 2000<br>class_weight = balanced<br>C = 0.1
	ANN	solver =adam<br>learning_rate_init = 0.01<br>hidden_layer_sizes = (50, 25)<br>batch_size = 32<br>alpha = 0.0001<br>activation = relu	solver =adam<br>learning_rate_init = 0.01<br>hidden_layer_sizes = (50, 25)<br>batch_size = 32<br>alpha = 0.0001<br>activation = relu	solver =adam<br>learning_rate_init = 0.01<br>hidden_layer_sizes = (50, 25)<br>batch_size = 32<br>alpha = 0.0001<br>activation = relu	solver =adam<br>learning_rate_init = 0.01<br>hidden_layer_sizes = (50, 25)<br>batch_size = 32<br>alpha = 0.0001<br>activation = relu
	LightGBM	subsample = 0.7<br>reg_lambda = 0.5<br>reg_alpha = 0.1<br>num_leaves = 63<br>n_estimators = 200<br>min_child_samples = 20<br>learning_rate = 0.05<br>colsample_bytree = 0.9	subsample = 0.7<br>reg_lambda = 0.5<br>reg_alpha = 0.1<br>num_leaves = 63<br>n_estimators = 200<br>min_child_samples = 20<br>learning_rate = 0.05<br>colsample_bytree = 0.9	subsample = 0.7<br>reg_lambda = 0.5<br>reg_alpha = 0.1<br>num_leaves = 63<br>n_estimators = 200<br>min_child_samples = 20<br>learning_rate = 0.05<br>colsample_bytree = 0.9	subsample = 0.7<br>reg_lambda = 0.5<br>reg_alpha = 0.1<br>num_leaves = 63<br>n_estimators = 200<br>min_child_samples = 20<br>learning_rate = 0.05<br>colsample_bytree = 0.9
	Random Forest	n_estimators = 300<br>min_samples_split = 2<br>min_samples_leaf = 1<br>max_samples = 0.8<br>max_features = sqrt<br>max_depth = 10<br>bootstrap = TRUE	n_estimators = 300<br>min_samples_split = 2<br>min_samples_leaf = 1<br>max_samples = 0.8<br>max_features = sqrt<br>max_depth = 10<br>bootstrap = TRUE	n_estimators = 300<br>min_samples_split = 2<br>min_samples_leaf = 1<br>max_samples = 0.8<br>max_features = sqrt<br>max_depth = 10<br>bootstrap = TRUE	n_estimators = 300<br>min_samples_split = 2<br>min_samples_leaf = 1<br>max_samples = 0.8<br>max_features = sqrt<br>max_depth = 10<br>bootstrap = TRUE
	CatBoost	random_strength = 1<br>learning_rate = 0.1<br>l2_leaf_reg = 3<br>iterations = 300<br>depth = 6	random_strength = 1<br>learning_rate = 0.1<br>l2_leaf_reg = 3<br>iterations = 300<br>depth = 6	random_strength = 1<br>learning_rate = 0.1<br>l2_leaf_reg = 3<br>iterations = 300<br>depth = 6	random_strength = 1<br>learning_rate = 0.1<br>l2_leaf_reg = 3<br>iterations = 300<br>depth = 6
	XGBoost	subsample = 0.7<br>reg_lambda = 0.1<br>reg_alpha = 0<br>n_estimators = 300<br>min_child_weight = 1<br>max_depth = 5<br>learning_rate = 0.05<br>gamma = 0.2<br>colsample_bytree = 0.8	subsample = 0.7<br>reg_lambda = 0.1<br>reg_alpha = 0<br>n_estimators = 300<br>min_child_weight = 1<br>max_depth = 5<br>learning_rate = 0.05<br>gamma = 0.2<br>colsample_bytree = 0.8	subsample = 0.9<br>reg_lambda = 0.1<br>reg_alpha = 0<br>n_estimators = 100<br>min_child_weight = 3<br>max_depth = 7<br>learning_rate = 0.05<br>gamma = 0<br>colsample_bytree = 0.9	subsample = 0.7<br>reg_lambda = 0.1<br>reg_alpha = 0<br>n_estimators = 300<br>min_child_weight = 1<br>max_depth = 5<br>learning_rate = 0.05<br>gamma = 0.2<br>colsample_bytree = 0.8

	Stacking ensemble	Base models: the above six optimal models<br>meta_learner =LogisticRegression (C=0.1, random_state=self.random_state, max_iter=500)	Base models: the above six optimal models<br>meta_learner =LogisticRegression (C=0.1, random_state=self.random_state, max_iter=500)	Base models: the above six optimal models<br>meta_learner =LogisticRegression (C=0.1, random_state=self.random_state, max_iter=500)	Base models: the above six optimal models<br>meta_learner =LogisticRegression (C=0.1, random_state=self.random_state, max_iter=500)
Late pregnancy	Logistic Regression	solver = liblinear<br>penalty = l2<br>max_iter = 2000<br>class_weight = balanced<br>C = 0.01	solver = lbfgs<br>penalty = l2<br>max_iter = 2000<br>class_weight = balanced<br>C = 1000	solver = liblinear<br>penalty = l1<br>max_iter = 2000<br>class_weight = balanced<br>C = 1000	solver = liblinear<br>penalty = l2<br>max_iter = 2000<br>class_weight = balanced<br>C = 0.01
	ANN	solver =adam<br>learning_rate_init = 0.01<br>hidden_layer_sizes = (50, 25)<br>batch_size = 32<br>alpha = 0.0001<br>activation = relu	solver =adam<br>learning_rate_init = 0.01<br>hidden_layer_sizes = (50, 25)<br>batch_size = 32<br>alpha = 0.0001<br>activation = relu	solver =adam<br>learning_rate_init = 0.01<br>hidden_layer_sizes = (50, 25)<br>batch_size = 32<br>alpha = 0.0001<br>activation = relu	solver =adam<br>learning_rate_init = 0.01<br>hidden_layer_sizes = (50, 25)<br>batch_size = 32<br>alpha = 0.0001<br>activation = relu
	LightGBM	subsample = 0.7<br>reg_lambda = 0.5<br>reg_alpha = 0.1<br>num_leaves = 63<br>n_estimators = 200<br>min_child_samples = 20<br>learning_rate = 0.05<br>colsample_bytree = 0.9	subsample = 0.7<br>reg_lambda = 0.5<br>reg_alpha = 0.1<br>num_leaves = 63<br>n_estimators = 200<br>min_child_samples = 20<br>learning_rate = 0.05<br>colsample_bytree = 0.9	subsample = 0.7<br>reg_lambda = 0.5<br>reg_alpha = 0.1<br>num_leaves = 63<br>n_estimators = 200<br>min_child_samples = 20<br>learning_rate = 0.05<br>colsample_bytree = 0.9	subsample = 0.7<br>reg_lambda = 0.5<br>reg_alpha = 0.1<br>num_leaves = 63<br>n_estimators = 200<br>min_child_samples = 20<br>learning_rate = 0.05<br>colsample_bytree = 0.9
	Random Forest	n_estimators = 300<br>min_samples_split = 2<br>min_samples_leaf = 1<br>max_samples = 0.8<br>max_features = sqrt<br>max_depth = 10<br>bootstrap = TRUE	n_estimators = 300<br>min_samples_split = 2<br>min_samples_leaf = 1<br>max_samples = 0.8<br>max_features = sqrt<br>max_depth = 10<br>bootstrap = TRUE	n_estimators = 300<br>min_samples_split = 2<br>min_samples_leaf = 1<br>max_samples = 0.8<br>max_features = sqrt<br>max_depth = 10<br>bootstrap = TRUE	n_estimators = 300<br>min_samples_split = 2<br>min_samples_leaf = 1<br>max_samples = 0.8<br>max_features = sqrt<br>max_depth = 10<br>bootstrap = TRUE
	CatBoost	random_strength = 1<br>learning_rate = 0.1<br>l2_leaf_reg = 3<br>iterations = 300<br>depth = 6	random_strength = 1<br>learning_rate = 0.1<br>l2_leaf_reg = 3<br>iterations = 300<br>depth = 6	random_strength = 1<br>learning_rate = 0.1<br>l2_leaf_reg = 3<br>iterations = 300<br>depth = 6	random_strength = 1<br>learning_rate = 0.1<br>l2_leaf_reg = 3<br>iterations = 300<br>depth = 6
	XGBoost	subsample = 0.7<br>reg_lambda = 0.1<br>reg_alpha = 0<br>n_estimators = 300<br>min_child_weight = 1<br>max_depth = 5<br>learning_rate = 0.05<br>gamma = 0.2<br>colsample_bytree = 0.8	subsample = 0.7<br>reg_lambda = 0.1<br>reg_alpha = 0<br>n_estimators = 300<br>min_child_weight = 1<br>max_depth = 5<br>learning_rate = 0.05<br>gamma = 0.2<br>colsample_bytree = 0.8	subsample = 0.7<br>reg_lambda = 0.1<br>reg_alpha = 0<br>n_estimators = 300<br>min_child_weight = 1<br>max_depth = 5<br>learning_rate = 0.05<br>gamma = 0.2<br>colsample_bytree = 0.8	subsample = 0.7<br>reg_lambda = 0.1<br>reg_alpha = 0<br>n_estimators = 300<br>min_child_weight = 1<br>max_depth = 5<br>learning_rate = 0.05<br>gamma = 0.2<br>colsample_bytree = 0.8

	Stacking ensemble	Base models: the above six optimal models<br>meta_learner =LogisticRegression (C=0.1, random_state=self.random_state, max_iter=500)	Base models: the above six optimal models<br>meta_learner =LogisticRegression (C=0.1, random_state=self.random_state, max_iter=500)	Base models: the above six optimal models<br>meta_learner =LogisticRegression (C=0.1, random_state=self.random_state, max_iter=500)	Base models: the above six optimal models<br>meta_learner =LogisticRegression (C=0.1, random_state=self.random_state, max_iter=500)

ANN= artificial neural networks..

The hyper-paramters for each model represents as the format of Python code.

Supplementary Table S8. Bootstrap Validation of prediction model performance using testing data set from the imputed data..

Models	China Standard							Intergrowth 21th Standard						
	AUC (95%CI)	Sen. (95%CI)	Spe. (95%CI)	Acc. (95%CI)	PPV (95%CI)	NPV (95%CI)	F1 score (95%CI)	AUC (95%CI)	Sen. (95%CI)	Spe. (95%CI)	Acc. (95%CI)	PPV (95%CI)	NPV (95%CI)	F1 score (95%CI)
**Early pregnancy**														
Catboost	0.53 (0.52, 0.53)	0.67 (0.41, 0.84)	0.37 (0.20, 0.63)	0.39 (0.24, 0.61)	0.07 (0.07, 0.08)	0.94 (0.93, 0.94)	0.05 (0.03, 0.06)	0.54 (0.53, 0.55)	0.62 (0.38, 0.87)	0.44 (0.18, 0.69)	0.45 (0.22, 0.67)	0.05 (0.05, 0.06)	0.96 (0.95, 0.96)	0.06 (0.05, 0.08)
XGBoost	0.55 (0.54, 0.56)	0.73 (0.59, 0.80)	0.35 (0.28, 0.50)	0.38 (0.32, 0.50)	0.08 (0.07, 0.08)	0.95 (0.94, 0.95)	0.08 (0.07, 0.09)	0.52 (0.51, 0.52)	0.71 (0.49, 0.78)	0.33 (0.27, 0.55)	0.35 (0.29, 0.55)	0.05 (0.05, 0.05)	0.96 (0.95, 0.96)	0.04 (0.02, 0.05)
LightBoost	0.54 (0.53, 0.54)	0.69 (0.61, 0.76)	0.38 (0.31, 0.45)	0.40 (0.34, 0.46)	0.08 (0.07, 0.08)	0.94 (0.94, 0.95)	0.07 (0.06, 0.08)	0.52 (0.51, 0.53)	0.71 (0.61, 0.77)	0.35 (0.03, 0.46)	0.37 (0.32, 0.47)	0.05 (0.05, 0.06)	0.96 (0.96, 0.96)	0.06 (0.05, 0.08)
Random Forest	0.56 (0.56, 0.57)	0.69 (0.66, 0.76)	0.42 (0.35, 0.45)	0.44 (0.38, 0.47)	0.08 (0.08, 0.08)	0.95 (0.95, 0.95)	0.11 (0.10, 0.13)	0.58 (0.56, 0.58)	0.58 (0.51, 0.66)	0.55 (0.47, 0.62)	0.55 (0.48, 0.62)	0.06 (0.06, 0.07)	0.96 (0.96, 0.96)	0.13 (0.11, 0.15)
ANN	0.62 (0.61, 0.63)	0.62 (0.57, 0.72)	0.56 (0.46, 0.60)	0.56 (0.48, 0.60)	0.09 (0.09, 0.10)	0.95 (0.95, 0.96)	0.18 (0.16, 0.19)	0.58 (0.58, 0.59)	0.76 (0.74, 0.80)	0.40 (0.36, 0.41)	0.41 (0.38, 0.43)	0.06 (0.06, 0.06)	0.97 (0.97, 0.97)	0.15 (0.14, 0.17)
Stacking ensemble	0.55 (0.55, 0.56)	0.68 (0.60, 0.75)	0.42 (0.33, 0.50)	0.43 (0.36, 0.50)	0.08 (0.08, 0.08)	0.95 (0.94, 0.95)	0.09 (0.08, 0.11)	0.58 (0.57, 0.59)	0.44 (0.38, 0.50)	0.70 (0.65, 0.76)	0.69 (0.64, 0.75)	0.07 (0.07, 0.08)	0.96 (0.96, 0.96)	0.14 (0.13, 0.16)
Logistic regression	0.67 (0.66, 0.68)	0.64 (0.58, 0.73)	0.62 (0.53, 0.67)	0.62 (0.54, 0.66)	0.11 (0.10, 0.12)	0.96 (0.96, 0.96)	0.25 (0.24, 0.27)	0.67 (0.66, 0.68)	0.55 (0.52, 0.59)	0.71 (0.66, 0.72)	0.70 (0.66, 0.71)	0.09 (0.08, 0.09)	0.97 (0.97, 0.97)	0.25 (0.24, 0.27)
**Middle pregnancy**														
Catboost	0.54 (0.54, 0.55)	0.75 (0.48, 0.83)	0.32 (0.24, 0.57)	0.35 (0.28, 0.57)	0.08 (0.07, 0.08)	0.95 (0.94, 0.95)	0.07 (0.06, 0.08)	0.60 (0.59, 0.61)	0.59 (0.52, 0.71)	0.55 (0.43, 0.61)	0.56 (0.45, 0.61)	0.06 (0.06, 0.07)	0.96 (0.96, 0.97)	0.15 (0.13, 0.16)
XGBoost	0.53 (0.52, 0.54)	0.58 (0.51, 0.67)	0.48 (0.38, 0.54)	0.48 (0.40, 0.54)	0.08 (0.07, 0.08)	0.94 (0.94, 0.94)	0.06 (0.04, 0.07)	0.51 (0.50, 0.52)	0.65 (0.51, 0.87)	0.38 (0.15, 0.52)	0.40 (0.18, 0.52)	0.05 (0.05, 0.05)	0.96 (0.95, 0.96)	0.03 (0.02, 0.05)
LightBoost	0.53 (0.52, 0.54)	0.71 (0.58, 0.84)	0.35 (0.22, 0.48)	0.37 (0.26, 0.49)	0.07 (0.07, 0.08)	0.94 (0.94, 0.95)	0.06 (0.05, 0.07)	0.55 (0.54, 0.56)	0.68 (0.63, 0.75)	0.42 (0.34, 0.47)	0.43 (0.36, 0.47)	0.06 (0.05, 0.06)	0.96 (0.96, 0.96)	0.09 (0.08, 0.11)
Random Forest	0.58 (0.57, 0.59)	0.67 (0.61, 0.74)	0.46 (0.38, 0.52)	0.48 (0.41, 0.53)	0.09 (0.08, 0.09)	0.95 (0.95, 0.95)	0.14 (0.12, 0.15)	0.59 (0.57, 0.60)	0.52 (0.44, 0.67)	0.62 (0.46, 0.70)	0.62 (0.47, 0.68)	0.07 (0.06, 0.07)	0.96 (0.96, 0.97)	0.14 (0.12, 0.15)
ANN	0.65 (0.64, 0.65)	0.61 (0.50, 0.70)	0.61 (0.51, 0.70)	0.61 (0.52, 0.69)	0.10 (0.10, 0.11)	0.95 (0.95, 0.96)	0.21 (0.20, 0.23)	0.60 (0.60, 0.62)	0.57 (0.51, 0.65)	0.59 (0.51, 0.65)	0.59 (0.51, 0.65)	0.07 (0.06, 0.07)	0.96 (0.96, 0.97)	0.16 (0.14, 0.18)
Stacking ensemble	0.56 (0.55, 0.57)	0.58 (0.42, 0.70)	0.51 (0.39, 0.67)	0.51 (0.41, 0.65)	0.08 (0.08, 0.09)	0.94 (0.94, 0.95)	0.09 (0.08, 0.11)	0.59 (0.58, 0.60)	0.48 (0.35, 0.61)	0.67 (0.54, 0.80)	0.66 (0.54, 0.77)	0.07 (0.06, 0.08)	0.96 (0.96, 0.96)	0.15 (0.14, 0.17)
Logistic regression	0.67 (0.66, 0.68)	0.63 (0.58, 0.69)	0.63 (0.57, 0.68)	0.63 (0.58, 0.67)	0.11 (0.10, 0.12)	0.96 (0.95, 0.96)	0.26 (0.24, 0.27)	0.67 (0.66, 0.68)	0.56 (0.52, 0.63)	0.69 (0.63, 0.72)	0.68 (0.63, 0.71)	0.09 (0.08, 0.09)	0.97 (0.97, 0.97)	0.25 (0.24, 0.27)
**Late pregnancy**														
Catboost	0.61 (0.6, 0.61)	0.65 (0.57, 0.72)	0.52 (0.44, 0.59)	0.52 (0.46, 0.58)	0.09 (0.09, 0.09)	0.95 (0.95, 0.96)	0.16 (0.15, 0.18)	0.63 (0.62, 0.63)	0.68 (0.54, 0.73)	0.52 (0.47, 0.65)	0.52 (0.48, 0.65)	0.07 (0.06, 0.07)	0.97 (0.97, 0.97)	0.19 (0.18, 0.21)
XGBoost	0.62 (0.62, 0.63)	0.69 (0.61, 0.77)	0.50 (0.42, 0.57)	0.51 (0.45, 0.58)	0.09 (0.09, 0.10)	0.96 (0.95, 0.96)	0.19 (0.18, 0.20)	0.57 (0.56, 0.58)	0.53 (0.48, 0.67)	0.57 (0.42, 0.62)	0.57 (0.44, 0.62)	0.06 (0.06, 0.06)	0.96 (0.96, 0.96)	0.10 (0.09, 0.12)
LightBoost	0.60 (0.59, 0.60)	0.67 (0.58, 0.74)	0.49 (0.42, 0.57)	0.50 (0.44, 0.57)	0.09 (0.08, 0.09)	0.95 (0.95, 0.96)	0.15 (0.14, 0.16)	0.58 (0.58, 0.59)	0.81 (0.76, 0.85)	0.35 (0.31, 0.40)	0.37 (0.33, 0.42)	0.06 (0.06, 0.06)	0.97 (0.97, 0.98)	0.16 (0.15, 0.17)
Random Forest	0.67 (0.67, 0.68)	0.65 (0.62, 0.71)	0.61 (0.54, 0.63)	0.62 (0.55, 0.63)	0.11 (0.10, 0.11)	0.96 (0.96, 0.96)	0.26 (0.25, 0.28)	0.70 (0.69, 0.71)	0.68 (0.58, 0.74)	0.61 (0.56, 0.72)	0.62 (0.57, 0.71)	0.08 (0.08, 0.09)	0.97 (0.97, 0.98)	0.30 (0.28, 0.31)
ANN	0.71 (0.70, 0.72)	0.69 (0.66, 0.74)	0.61 (0.56, 0.64)	0.62 (0.57, 0.64)	0.12 (0.11, 0.12)	0.96 (0.96, 0.97)	0.30 (0.29, 0.32)	0.69 (0.68, 0.70)	0.66 (0.59, 0.72)	0.62 (0.57, 0.69)	0.62 (0.57, 0.68)	0.08 (0.08, 0.09)	0.97 (0.97, 0.98)	0.28 (0.27, 0.30)
Stacking ensemble	0.67 (0.66, 0.68)	0.62 (0.54, 0.68)	0.63 (0.57, 0.70)	0.63 (0.58, 0.68)	0.11 (0.10, 0.12)	0.96 (0.95, 0.96)	0.25 (0.24, 0.26)	0.64 (0.63, 0.65)	0.53 (0.49, 0.60)	0.69 (0.63, 0.72)	0.69 (0.63, 0.71)	0.08 (0.07, 0.09)	0.97 (0.96, 0.97)	0.22 (0.21, 0.24)
Logistic regression	0.72 (0.72, 0.73)	0.67 (0.63, 0.75)	0.66 (0.58, 0.70)	0.66 (0.59, 0.70)	0.13 (0.11, 0.14)	0.96 (0.96, 0.97)	0.33 (0.32, 0.34)	0.74 (0.73, 0.74)	0.69 (0.63, 0.78)	0.67 (0.58, 0.72)	0.67 (0.59, 0.71)	0.10 (0.09, 0.11)	0.98 (0.97, 0.98)	0.36 (0.34, 0.37)

Continue

Models	GROW Standard							FMF Standard						
	AUC (95%CI)	Sen. (95%CI)	Spe. (95%CI)	Acc. (95%CI)	PPV (95%CI)	NPV (95%CI)	F1 score (95%CI)	AUC (95%CI)	Sen. (95%CI)	Spe. (95%CI)	Acc. (95%CI)	PPV (95%CI)	NPV (95%CI)	F1 score (95%CI)
Early pregnancy														
Catboost	0.52 (0.51, 0.53)	0.46 (0.26, 0.81)	0.58 (0.22, 0.77)	0.57 (0.27, 0.74)	0.08 (0.07, 0.09)	0.93 (0.93, 0.94)	0.04 (0.02, 0.05)	0.54 (0.54, 0.55)	0.66 (0.42, 0.86)	0.41 (0.20, 0.64)	0.43 (0.27, 0.62)	0.12 (0.11, 0.13)	0.91 (0.90, 0.92)	0.06 (0.05, 0.08)
XGBoost	0.54 (0.53, 0.55)	0.38 (0.26, 0.63)	0.68 (0.42, 0.81)	0.66 (0.44, 0.77)	0.09 (0.08, 0.10)	0.93 (0.93, 0.94)	0.07 (0.05, 0.08)	0.57 (0.56, 0.57)	0.61 (0.45, 0.71)	0.48 (0.39, 0.65)	0.50 (0.42, 0.63)	0.13 (0.12, 0.13)	0.91 (0.91, 0.92)	0.10 (0.09, 0.11)
LightBoost	0.52 (0.51, 0.52)	0.75 (0.66, 0.78)	0.29 (0.26, 0.38)	0.33 (0.30, 0.40)	0.08 (0.07, 0.08)	0.94 (0.93, 0.94)	0.04 (0.03, 0.05)	0.53 (0.52, 0.54)	0.74 (0.62, 0.83)	0.31 (0.22, 0.44)	0.36 (0.28, 0.46)	0.11 (0.11, 0.12)	0.91 (0.90, 0.92)	0.05 (0.04, 0.06)
Random Forest	0.51 (0.51, 0.52)	0.71 (0.59, 0.86)	0.32 (0.17, 0.44)	0.35 (0.22, 0.45)	0.08 (0.07, 0.08)	0.94 (0.93, 0.94)	0.04 (0.03, 0.05)	0.57 (0.56, 0.57)	0.73 (0.67, 0.80)	0.38 (0.31, 0.45)	0.42 (0.36, 0.47)	0.12 (0.12, 0.13)	0.92 (0.92, 0.93)	0.11 (0.10, 0.12)
ANN	0.53 (0.52, 0.54)	0.68 (0.64, 0.71)	0.38 (0.35, 0.41)	0.40 (0.38, 0.43)	0.08 (0.08, 0.08)	0.94 (0.93, 0.94)	0.06 (0.05, 0.07)	0.64 (0.63, 0.64)	0.66 (0.63, 0.69)	0.55 (0.52, 0.57)	0.56 (0.54, 0.58)	0.15 (0.14, 0.16)	0.93 (0.93, 0.93)	0.21 (0.20, 0.22)
Stacking ensemble	0.52 (0.51, 0.53)	0.26 (0.16, 0.62)	0.78 (0.42, 0.88)	0.74 (0.43, 0.83)	0.09 (0.08, 0.10)	0.93 (0.93, 0.93)	0.04 (0.03, 0.05)	0.57 (0.57, 0.58)	0.50 (0.44, 0.61)	0.61 (0.50, 0.67)	0.60 (0.51, 0.65)	0.13 (0.13, 0.14)	0.91 (0.91, 0.92)	0.11 (0.10, 0.13)
Logistic regression	0.54 (0.53, 0.55)	0.61 (0.24, 0.77)	0.45 (0.30, 0.81)	0.46 (0.33, 0.77)	0.08 (0.08, 0.10)	0.94 (0.93, 0.94)	0.06 (0.05, 0.07)	0.66 (0.65, 0.67)	0.60 (0.52, 0.66)	0.63 (0.58, 0.71)	0.63 (0.58, 0.69)	0.16 (0.15, 0.18)	0.93 (0.92, 0.93)	0.23 (0.22, 0.24)
Middle pregnancy														
Catboost	0.51 (0.50, 0.52)	0.39 (0.26, 0.80)	0.64 (0.22, 0.77)	0.62 (0.26, 0.73)	0.08 (0.07, 0.08)	0.93 (0.93, 0.94)	0.03 (0.02, 0.04)	0.57 (0.57, 0.58)	0.64 (0.58, 0.74)	0.47 (0.37, 0.53)	0.49 (0.41, 0.53)	0.13 (0.12, 0.13)	0.92 (0.91, 0.92)	0.11 (0.10, 0.12)
XGBoost	0.52 (0.51, 0.53)	0.38 (0.25, 0.63)	0.66 (0.41, 0.78)	0.64 (0.42, 0.74)	0.08 (0.08, 0.09)	0.93 (0.93, 0.93)	0.04 (0.03, 0.05)	0.55 (0.54, 0.56)	0.66 (0.52, 0.71)	0.41 (0.36, 0.56)	0.44 (0.40, 0.55)	0.12 (0.12, 0.12)	0.91 (0.90, 0.91)	0.07 (0.06, 0.09)
LightBoost	0.52 (0.51, 0.53)	0.73 (0.28, 0.84)	0.31 (0.20, 0.76)	0.34 (0.25, 0.73)	0.08 (0.07, 0.08)	0.94 (0.93, 0.94)	0.04 (0.03, 0.05)	0.52 (0.51, 0.53)	0.83 (0.76, 0.85)	0.22 (0.20, 0.28)	0.28 (0.27, 0.33)	0.11 (0.11, 0.12)	0.91 (0.91, 0.92)	0.05 (0.04, 0.06)
Random Forest	0.52 (0.51, 0.53)	0.69 (0.64, 0.76)	0.36 (0.28, 0.41)	0.39 (0.31, 0.42)	0.08 (0.08, 0.08)	0.94 (0.93, 0.94)	0.05 (0.03, 0.06)	0.57 (0.57, 0.58)	0.70 (0.61, 0.78)	0.42 (0.34, 0.51)	0.45 (0.39, 0.52)	0.13 (0.12, 0.13)	0.92 (0.92, 0.93)	0.12 (0.11, 0.13)
ANN	0.51 (0.51, 0.52)	0.75 (0.66, 0.79)	0.29 (0.25, 0.39)	0.32 (0.29, 0.41)	0.08 (0.07, 0.08)	0.94 (0.93, 0.94)	0.04 (0.02, 0.05)	0.62 (0.61, 0.62)	0.63 (0.54, 0.71)	0.54 (0.47, 0.63)	0.55 (0.49, 0.62)	0.14 (0.14, 0.15)	0.92 (0.92, 0.93)	0.17 (0.16, 0.18)
Stacking ensemble	0.50 (0.49, 0.51)	0.55 (0.12, 0.70)	0.47 (0.31, 0.89)	0.47 (0.34, 0.83)	0.08 (0.07, 0.08)	0.93 (0.93, 0.93)	0.02 (0.00, 0.03)	0.56 (0.55, 0.57)	0.55 (0.51, 0.63)	0.54 (0.46, 0.60)	0.55 (0.48, 0.59)	0.13 (0.12, 0.13)	0.91 (0.91, 0.91)	0.10 (0.09, 0.11)
Logistic regression	0.54 (0.53, 0.55)	0.62 (0.27, 0.83)	0.44 (0.24, 0.79)	0.45 (0.28, 0.75)	0.08 (0.08, 0.09)	0.94 (0.93, 0.95)	0.06 (0.05, 0.07)	0.66 (0.65, 0.67)	0.60 (0.52, 0.65)	0.63 (0.58, 0.71)	0.63 (0.59, 0.69)	0.16 (0.16, 0.18)	0.93 (0.92, 0.93)	0.23 (0.22, 0.24)
Late pregnancy														
Catboost	0.56 (0.55, 0.57)	0.65 (0.58, 0.76)	0.44 (0.34, 0.51)	0.46 (0.37, 0.51)	0.08 (0.08, 0.09)	0.94 (0.94, 0.95)	0.09 (0.08, 0.1)	0.63 (0.63, 0.64)	0.65 (0.58, 0.74)	0.54 (0.46, 0.62)	0.56 (0.49, 0.61)	0.15 (0.14, 0.15)	0.93 (0.92, 0.94)	0.19 (0.18, 0.20)
XGBoost	0.65 (0.65, 0.66)	0.71 (0.65, 0.76)	0.53 (0.48, 0.58)	0.54 (0.50, 0.59)	0.11 (0.10, 0.11)	0.96 (0.95, 0.96)	0.24 (0.22, 0.25)	0.64 (0.64, 0.65)	0.64 (0.54, 0.70)	0.58 (0.51, 0.67)	0.58 (0.53, 0.66)	0.15 (0.14, 0.17)	0.93 (0.92, 0.93)	0.21 (0.20, 0.22)
LightBoost	0.56 (0.55, 0.57)	0.81 (0.75, 0.88)	0.30 (0.23, 0.36)	0.34 (0.28, 0.39)	0.08 (0.08, 0.09)	0.95 (0.95, 0.96)	0.11 (0.10, 0.12)	0.61 (0.60, 0.61)	0.71 (0.59, 0.76)	0.45 (0.40, 0.58)	0.48 (0.44, 0.58)	0.14 (0.13, 0.14)	0.93 (0.92, 0.93)	0.16 (0.15, 0.17)
Random Forest	0.56 (0.56, 0.57)	0.81 (0.76, 0.86)	0.31 (0.26, 0.35)	0.34 (0.30, 0.38)	0.08 (0.08, 0.09)	0.95 (0.95, 0.96)	0.12 (0.11, 0.13)	0.69 (0.68, 0.69)	0.71 (0.67, 0.76)	0.56 (0.52, 0.61)	0.58 (0.55, 0.61)	0.16 (0.16, 0.17)	0.94 (0.94, 0.95)	0.28 (0.27, 0.29)
ANN	0.58 (0.57, 0.59)	0.55 (0.51, 0.58)	0.58 (0.56, 0.62)	0.58 (0.56, 0.61)	0.09 (0.09, 0.10)	0.94 (0.94, 0.95)	0.14 (0.12, 0.15)	0.70 (0.69, 0.71)	0.69 (0.64, 0.74)	0.62 (0.57, 0.66)	0.63 (0.59, 0.66)	0.18 (0.17, 0.19)	0.94 (0.94, 0.95)	0.30 (0.29, 0.32)
Stacking ensemble	0.63 (0.62, 0.64)	0.64 (0.56, 0.73)	0.55 (0.45, 0.64)	0.56 (0.47, 0.63)	0.10 (0.09, 0.11)	0.95 (0.95, 0.96)	0.19 (0.18, 0.20)	0.67 (0.67, 0.68)	0.66 (0.62, 0.69)	0.60 (0.57, 0.64)	0.60 (0.59, 0.64)	0.16 (0.16, 0.17)	0.94 (0.93, 0.94)	0.26 (0.25, 0.27)
Logistic regression	0.62 (0.61, 0.62)	0.59 (0.51, 0.63)	0.58 (0.54, 0.66)	0.59 (0.55, 0.65)	0.10 (0.10, 0.11)	0.95 (0.94, 0.95)	0.18 (0.16, 0.19)	0.72 (0.71, 0.72)	0.69 (0.65, 0.74)	0.64 (0.59, 0.66)	0.64 (0.61, 0.66)	0.18 (0.18, 0.19)	0.94 (0.94, 0.95)	0.32 (0.31, 0.33)

ANN, artificial neural networks. AUC, area under the ROC curve; Sen., Sensitivity; Spe.,Specificity;Acc.,Accuracy;PPV, Positive predictive value;NPV, Negative predictive value.
.

Supplementary Table S9. Hyper-parameters of each prediction model using the imputed data for the four birthweight standards.

Pregnancy intervals	Models	Hyper-parameters used in prediction models			
		China Standard	Intergrowth 21th standard	GROW standard	FMF standard
Early pregnancy	Logistic Regression	solver = saga<br>penalty = l2<br>max_iter = 2000<br>class_weight = balanced<br>C = 0.1	solver = saga<br>penalty = l2<br>max_iter = 2000<br>class_weight = balanced<br>C = 0.1	solver = liblinear<br>penalty = l1<br>max_iter = 2000<br>class_weight = balanced<br>C = 0.01	solver = saga<br>penalty = l2<br>max_iter = 2000<br>class_weight = balanced<br>C = 0.1
	ANN	solver =adam<br>learning_rate_init = 0.001<br>hidden_layer_sizes = (50, 25)<br>batch_size = 32<br>alpha = 0.001<br>activation = tanh	solver =adam<br>learning_rate_init = 0.01<br>hidden_layer_sizes = (50, 25)<br>batch_size = 32<br>alpha = 0.0001<br>activation =relu	solver =adam<br>learning_rate_init = 0.001<br>hidden_layer_sizes = (50, 25)<br>batch_size = 64<br>alpha = 0.0001<br>activation =tanh	solver =adam<br>learning_rate_init = 0.01<br>hidden_layer_sizes = (50, 25)<br>batch_size = 32<br>alpha = 0.0001<br>activation =relu
	LightGBM	subsample = 0.9<br>reg_lambda = 0.1<br>reg_alpha = 0.5<br>num_leaves = 63<br>n_estimators = 200<br>min_child_samples = 20<br>learning_rate = 0.05<br>colsample_bytree = 0.7	subsample = 0.9<br>reg_lambda = 0.5<br>reg_alpha = 0.1<br>num_leaves = 63<br>n_estimators = 100<br>min_child_samples = 50<br>learning_rate = 0.05<br>colsample_bytree = 0.9	subsample = 0.8<br>reg_lambda =0<br>reg_alpha = 0.5<br>num_leaves = 63<br>n_estimators = 200<br>min_child_samples = 20<br>learning_rate = 0.1<br>colsample_bytree = 0.8	subsample = 0.9<br>reg_lambda = 0.1<br>reg_alpha = 0.5<br>num_leaves = 63<br>n_estimators = 200<br>min_child_samples = 20<br>learning_rate = 0.05<br>colsample_bytree = 0.7
	Random Forest	n_estimators = 100<br>min_samples_split = 2<br>min_samples_leaf = 1<br>max_samples = 0.7<br>max_features = sqrt<br>max_depth = 10<br>bootstrap = TRUE	n_estimators = 100<br>min_samples_split = 10<br>min_samples_leaf = 4<br>max_samples = 0.7<br>max_features = sqrt<br>max_depth = 10<br>bootstrap = TRUE	n_estimators = 100<br>min_samples_split = 2<br>min_samples_leaf = 1<br>max_samples = 0.7<br>max_features = sqrt<br>max_depth = 10<br>bootstrap = TRUE	n_estimators = 300<br>min_samples_split = 2<br>min_samples_leaf = 1<br>max_samples = 0.8<br>max_features = sqrt<br>max_depth = 10<br>bootstrap = TRUE
	CatBoost	random_strength = 1<br>learning_rate = 0.1<br>l2_leaf_reg = 3<br>iterations = 300<br>depth = 6	random_strength = 1<br>learning_rate = 0.1<br>l2_leaf_reg = 3<br>iterations = 300<br>depth = 6	random_strength = 1<br>learning_rate = 0.1<br>l2_leaf_reg = 3<br>iterations = 300<br>depth = 6	random_strength = 1<br>learning_rate = 0.1<br>l2_leaf_reg = 3<br>iterations = 300<br>depth = 6
	XGBoost	subsample = 0.8<br>reg_lambda = 0<br>reg_alpha = 0<br>n_estimators = 200<br>min_child_weight = 5<br>max_depth = 5<br>learning_rate = 0.1<br>gamma = 0.2<br>colsample_bytree = 0.7	subsample = 0.8<br>reg_lambda = 0<br>reg_alpha = 0<br>n_estimators = 200<br>min_child_weight = 5<br>max_depth = 5<br>learning_rate = 0.1<br>gamma = 0.2<br>colsample_bytree = 0.7	subsample = 0.9<br>reg_lambda = 0.5<br>reg_alpha = 0.1<br>n_estimators = 200<br>min_child_weight = 1<br>max_depth = 5<br>learning_rate = 0.05<br>gamma = 0.2<br>colsample_bytree = 0.7	subsample = 0.8<br>reg_lambda = 0<br>reg_alpha = 0<br>n_estimators = 200<br>min_child_weight = 5<br>max_depth = 5<br>learning_rate = 0.1<br>gamma = 0.2<br>colsample_bytree = 0.7

	Stacking ensemble	Base models: the above six optimal models<br>meta_learner =LogisticRegression (C=0.1, random_state=self.random_state, max_iter=500)	Base models: the above six optimal models<br>meta_learner =LogisticRegression (C=0.1, random_state=self.random_state, max_iter=500)	Base models: the above six optimal models<br>meta_learner =LogisticRegression (C=0.1, random_state=self.random_state, max_iter=500)	Base models: the above six optimal models<br>meta_learner =LogisticRegression (C=0.1, random_state=self.random_state, max_iter=500)
Middle pregnancy	Logistic Regression	solver = saga<br>penalty = l2<br>max_iter = 2000<br>class_weight = balanced<br>C = 0.1	solver = saga<br>penalty = l2<br>max_iter = 2000<br>class_weight = balanced<br>C = 0.1	solver = lbfgs<br>penalty = l2<br>max_iter = 2000<br>class_weight = None<br>C = 1000	solver = saga<br>penalty = l2<br>max_iter = 2000<br>class_weight = balanced<br>C = 0.1
	ANN	solver =adam<br>learning_rate_init = 0.001<br>hidden_layer_sizes = (50, 25)<br>batch_size = 32<br>alpha = 0.001<br>activation = relu	solver =adam<br>learning_rate_init = 0.001<br>hidden_layer_sizes = (50, 25)<br>batch_size = 64<br>alpha = 0.0001<br>activation = tanh	solver =adam<br>learning_rate_init = 0.001<br>hidden_layer_sizes = (50, 25)<br>batch_size = 32<br>alpha = 0.001<br>activation = tanh	solver =adam<br>learning_rate_init = 0.001<br>hidden_layer_sizes = (50, 25)<br>batch_size = 32<br>alpha = 0.001<br>activation =tanh
	LightGBM	subsample = 0.9<br>reg_lambda = 0.1<br>reg_alpha = 0.5<br>num_leaves = 63<br>n_estimators = 200<br>min_child_samples = 20<br>learning_rate = 0.05<br>colsample_bytree = 0.7	subsample = 0.9<br>reg_lambda = 0.5<br>reg_alpha = 0.1<br>num_leaves = 63<br>n_estimators = 100<br>min_child_samples = 50<br>learning_rate = 0.05<br>colsample_bytree = 0.9	subsample = 0.9<br>reg_lambda = 0.1<br>reg_alpha = 0.5<br>num_leaves = 63<br>n_estimators = 200<br>min_child_samples = 20<br>learning_rate = 0.05<br>colsample_bytree = 0.7	subsample = 0.9<br>reg_lambda = 0.1<br>reg_alpha = 0.5<br>num_leaves = 63<br>n_estimators = 200<br>min_child_samples = 20<br>learning_rate = 0.05<br>colsample_bytree = 0.7
	Random Forest	n_estimators = 300<br>min_samples_split = 2<br>min_samples_leaf = 1<br>max_samples = 0.8<br>max_features = sqrt<br>max_depth = 10<br>bootstrap = TRUE	n_estimators = 300<br>min_samples_split = 2<br>min_samples_leaf = 1<br>max_samples = 0.8<br>max_features = sqrt<br>max_depth = 10<br>bootstrap = TRUE	n_estimators = 100<br>min_samples_split = 2<br>min_samples_leaf = 1<br>max_samples = 0.7<br>max_features = sqrt<br>max_depth = 10<br>bootstrap = TRUE	n_estimators = 300<br>min_samples_split = 2<br>min_samples_leaf = 1<br>max_samples = 0.8<br>max_features = sqrt<br>max_depth = 10<br>bootstrap = TRUE
	CatBoost	random_strength = 0.7<br>learning_rate =0.1<br>l2_leaf_reg = 3<br>iterations = 300<br>depth = 6	random_strength = 1<br>learning_rate =0.1<br>l2_leaf_reg = 3<br>iterations = 300<br>depth = 6	random_strength = 1<br>learning_rate =0.1<br>l2_leaf_reg = 3<br>iterations = 300<br>depth = 6	random_strength = 1<br>learning_rate =0.1<br>l2_leaf_reg = 3<br>iterations = 300<br>depth = 6
	XGBoost	subsample = 0.8<br>reg_lambda = 0<br>reg_alpha = 0<br>n_estimators = 200<br>min_child_weight = 5<br>max_depth = 5<br>learning_rate = 0.1<br>gamma = 0.2<br>colsample_bytree = 0.7	subsample = 0.8<br>reg_lambda = 0<br>reg_alpha = 0<br>n_estimators = 200<br>min_child_weight = 5<br>max_depth = 5<br>learning_rate = 0.1<br>gamma = 0.2<br>colsample_bytree = 0.7	subsample = 0.7<br>reg_lambda = 0.1<br>reg_alpha = 0<br>n_estimators = 300<br>min_child_weight = 1<br>max_depth = 5<br>learning_rate = 0.05<br>gamma = 0.2<br>colsample_bytree = 0.8	subsample = 0.8<br>reg_lambda = 0<br>reg_alpha = 0<br>n_estimators = 200<br>min_child_weight = 5<br>max_depth = 5<br>learning_rate = 0.1<br>gamma = 0.2<br>colsample_bytree = 0.7

	Stacking ensemble	Base models: the above six optimal models<br>meta_learner =LogisticRegression (C=0.1,<br>random_state=self.random_state,<br>max_iter=500)	Base models: the above six optimal models<br>meta_learner =LogisticRegression (C=0.1,<br>random_state=self.random_state,<br>max_iter=500)	Base models: the above six optimal models<br>meta_learner =LogisticRegression (C=0.1,<br>random_state=self.random_state,<br>max_iter=500)	Base models: the above six optimal models<br>meta_learner =LogisticRegression (C=0.1,<br>random_state=self.random_state,<br>max_iter=500)
Late pregnancy	Logistic Regression	solver = saga<br>penalty = l2<br>max_iter = 2000<br>class_weight = balanced<br>C = 0.1	solver = lbfgs<br>penalty = l2<br>max_iter = 2000<br>class_weight = balanced<br>C = 1000	solver = lbfgs<br>penalty = l2<br>max_iter = 2000<br>class_weight = None<br>C = 1000	solver = saga<br>penalty = l1<br>max_iter = 2000<br>class_weight = None<br>C = 10
	ANN	solver =adam<br>learning_rate_init = 0.001<br>hidden_layer_sizes = (50, 25)<br>batch_size = 32<br>alpha = 0.001<br>activation = tanh	solver =adam<br>learning_rate_init = 0.001<br>hidden_layer_sizes = (50, 25)<br>batch_size = 32<br>alpha = 0.001<br>activation = tanh	solver =adam<br>learning_rate_init = 0.001<br>hidden_layer_sizes = (50, 25)<br>batch_size = 64<br>alpha = 0.0001<br>activation = tanh	solver =adam<br>learning_rate_init = 0.01<br>hidden_layer_sizes = (50, 25)<br>batch_size = 32<br>alpha = 0.0001<br>activation = relu
	LightGBM	subsample = 0.7<br>reg_lambda = 0.5<br>reg_alpha = 0.1<br>num_leaves = 63<br>n_estimators = 200<br>min_child_samples = 20<br>learning_rate = 0.05<br>colsample_bytree = 0.9	subsample = 0.7<br>reg_lambda = 0.5<br>reg_alpha = 0.1<br>num_leaves = 63<br>n_estimators = 200<br>min_child_samples = 20<br>learning_rate = 0.05<br>colsample_bytree = 0.9	subsample = 0.9<br>reg_lambda = 0.1<br>reg_alpha = 0.5<br>num_leaves = 63<br>n_estimators = 200<br>min_child_samples = 20<br>learning_rate = 0.05<br>colsample_bytree = 0.7	subsample = 0.7<br>reg_lambda = 0.5<br>reg_alpha = 0.1<br>num_leaves = 63<br>n_estimators = 200<br>min_child_samples = 20<br>learning_rate = 0.05<br>colsample_bytree = 0.9
	Random Forest	n_estimators = 100<br>min_samples_split = 2<br>min_samples_leaf = 1<br>max_samples = 0.7<br>max_features = sqrt<br>max_depth = 10<br>bootstrap = TRUE	n_estimators = 100<br>min_samples_split = 2<br>min_samples_leaf = 1<br>max_samples = 0.8<br>max_features = sqrt<br>max_depth = 10<br>bootstrap = TRUE	n_estimators = 100<br>min_samples_split = 10<br>min_samples_leaf = 4<br>max_samples = 0.7<br>max_features = sqrt<br>max_depth = 10<br>bootstrap = TRUE	n_estimators = 300<br>min_samples_split = 2<br>min_samples_leaf = 1<br>max_samples = 0.8<br>max_features = sqrt<br>max_depth = 10<br>bootstrap = TRUE
	CatBoost	random_strength = 1<br>learning_rate =0.1<br>l2_leaf_reg = 3<br>iterations = 300<br>depth = 6	random_strength = 1<br>learning_rate =0.1<br>l2_leaf_reg = 3<br>iterations = 300<br>depth = 6	random_strength = 1<br>learning_rate =0.1<br>l2_leaf_reg = 3<br>iterations = 300<br>depth = 6	random_strength = 1<br>learning_rate =0.1<br>l2_leaf_reg = 3<br>iterations = 300<br>depth = 6
	XGBoost	subsample = 0.8<br>reg_lambda = 0<br>reg_alpha = 0<br>n_estimators = 200<br>min_child_weight = 5<br>max_depth = 5<br>learning_rate = 0.1<br>gamma = 0.2<br>colsample_bytree = 0.7	subsample = 0.8<br>reg_lambda = 0<br>reg_alpha = 0<br>n_estimators = 200<br>min_child_weight = 5<br>max_depth = 5<br>learning_rate = 0.1<br>gamma = 0.2<br>colsample_bytree = 0.7	subsample = 0.7<br>reg_lambda = 0.1<br>reg_alpha = 0<br>n_estimators = 300<br>min_child_weight = 1<br>max_depth = 5<br>learning_rate = 0.05<br>gamma = 0.2<br>colsample_bytree = 0.8	subsample = 0.8<br>reg_lambda = 0<br>reg_alpha = 0<br>n_estimators = 200<br>min_child_weight = 5<br>max_depth = 5<br>learning_rate = 0.1<br>gamma = 0.2<br>colsample_bytree = 0.7

	Stacking ensemble	Base models: the above six optimal models<br>meta_learner =LogisticRegression (C=0.1,<br>random_state=self.random_state,<br>max_iter=500)	Base models: the above six optimal models<br>meta_learner =LogisticRegression (C=0.1,<br>random_state=self.random_state,<br>max_iter=500)	Base models: the above six optimal models<br>meta_learner =LogisticRegression (C=0.1,<br>random_state=self.random_state,<br>max_iter=500)	Base models: the above six optimal models<br>meta_learner =LogisticRegression (C=0.1,<br>random_state=self.random_state,<br>max_iter=500)

.ANN= artificial neural networks..

The hyper-paramters for each model represents as the format of Python code

Supplementary Table S10. Delong test between the best-fitting model of each birthweight standard using the data with SGA defined as all of the four birthweight standards.

Model 1	Model 2	Z Value	P Value
Logistic regression for the China Standard	Logistic regression for the Iintergrowth 21st Standard	5.182	<0.0001
Logistic regression for the China Standard	Logistic regression for the Iintergrowth 21st Standard	-0.3329	0.7392
Logistic regression for the China Standard	Random Forest for the FMF Standard	45.8164	<0.0001
Logistic regression for the GROW Standard	Logistic regression for the Iintergrowth 21st Standard	-8.2749	<0.0001
Logistic regression for the GROW Standard	Random Forest for the FMF Standard	5.7767	<0.0001
Logistic regression for the Iintergrowth 21st Standard	Random Forest for the FMF Standard	67.0624	<0.0001

Supplementary Table S11. Mean SHAP value of each feature for four birthweight standards in the complete data.

Logistic regression model for China Standard			Logistic regression model for Intergrowth 21th Standard			Logistic regression model for GROW Standard			Random Forest model for FMF Standard		
Rank	Feature name	Mean SHAP value	Rank	Feature name	Mean SHAP value	Rank	Feature name	Mean SHAP value	Rank	Feature name	Mean SHAP value
1	SFH3	0.0558	1	Maternal weight before pregnancy	-0.0202	1	diffWEIGHT13	0.0096	1	SFH3	-0.0126
2	MAC3	0.0420	2	WEIGHT3	0.0120	2	diffWEIGHT23	0.0543	2	MAC3	-0.0100
3	Maternal height	0.0113	3	Maternal height	0.0062	3	diffWEIGHT12	-0.0817	3	Length of a menstrual cycle	0.0314
4	Maternal age	0.0547	4	SFH3	-0.0182	4	MAC3	-0.0297	4	WEIGHT3	-0.0068
5	Parity	0.0278	5	MAC3	0.0139	5	SFH3	-0.058	5	Parity	-0.0071
6	DBP3	-0.0084	6	Parity	0.0424	6	Maternal weight before pregnancy	0.0037	6	Maternal height	-0.0022
7	SBP1	0.0077	7	Maternal age	0.0138	7	FBG	-0.0082	7	Maternal age	0.0029
8	WEIGHT3	0.0145	8	DBP3	0.0129	8	Maternal age	0.0173	8	SFH2	0.0054
9	diffWEIGHT12	-0.0127	9	Menarche	0.0128	9	SBP3	0.0010	9	diffWEIGHT12	-0.0021
10	Scr at register	-0.011	10	SFH2	-0.0064	10	TBil at register	0.0076	10	DBP3	-0.0017
11	Menarche	-0.0016	11	diffWEIGHT13	-0.0062	11	Scr at register	-0.0073	11	diffWEIGHT13	-0.0026
12	Leukocyte at register	-0.0037	12	SBP3	0.0036	12	Leukocyte at register	-0.0072	12	Menarche	0.0091
13	Gestational weeks at 1st follow-up	-0.0084	13	diffDBP23	0.0080	13	BUN at register	-0.0104	13	AIB at register	-0.0005
14	diffDBP23	0.0011	14	diffWEIGHT12	0.0019	14	Menarche	-0.0065	14	diffMAC23	-0.001
15	Length of a menstrual period	0.0053	15	Gestational weeks at 1st follow-up	-0.0012	15	AIB at register	-0.0112	15	Length of a menstrual period	0.0025
16	AIB at register	-0.0086	16	Maternal heart rate	-0.0054	16	Length of a menstrual period	0.0038	16	SBP1	-0.0009
17	SFH2	0.0088	17	TBil at register	-0.0117	17	Length of a menstrual cycle	-0.0003	17	Scr at register	-0.0005
18	diffSBP23	0.0031	18	Length of a menstrual cycle	-0.001	18	frequencies of antenatal care	-0.0002	18	Education	-0.0020
19	diffSBP12	0.0013	19	diffMAC23	0.0045	19	Gynecology history	0.0040	19	Having maternal disease history	0.0047
20	Education	0	20	Leukocyte at register	-0.0076	20	SBP1	0.0028	20	diffDBP23	-0.0006
			21	AIB at register	-0.0071	21	FBG at register	0.0031	21	diffSBP23	-0.0008
			22	diffSBP23	0.0115	22	SFH2	-0.0013	22	Gestational weeks at first follow-up	0.0014
			23	DBP1	-0.0037	23	diffDBP23	-0.0023	23	ALT at register	-0.0005

	24	BUN at register	0.0067	24	Ethnicity	-0.0004	24	Platelet at register	-0.0005
	25	AST at register	-0.0001	25	Contraception	0.0002	25	FBG at register	-0.0005
	26	Platelet at register	-0.0010	26	Pregnancy complications	-0.0002	26	hemoglobin	-0.0003
	27	Ethnicity	-0.0001	27	Education	0	27	Leukocyte at register	-0.0006
	28	Having medicine use	-0.0005	28	Medicine use	-0.0003	28	BUN at register	-0.0004
	29	Having maternal disease history	0	29	Occupation	0	29	TBil at register	-0.0002
	30	Pregnancy complications	0.0001				30	Occupation	-0.0007
	31	smoking or drinking	0.0001				31	Ethnicity	-0.0005
	32	Contraception	0				32	Contracepion	-0.0003
	33	Occupation	0				33	Pregnancy complications	-0.0001
	34	Education	0				34	smoking or drinking	-0.0001

Supplementary Table S12. Mean SHAP value of each feature for four birthweight standards in the imputed data.

Logistic regression model for China Standard			Logistic regression model for Intergrowth 21th Standard			XGBoost model for GROW Standard			Logistic regression model for FMF Standard		
Rank	Feature name	Mean SHAP value	Rank	Feature name	Mean SHAP value	Rank	Feature name	Mean SHAP value	Rank	Feature name	Mean SHAP value
1	WEIGHT3	-0.0415	1	WEIGHT3	0.0086	1	Parity	-0.588	1	WEIGHT3	0.0491
2	SFH3	0.0385	2	SFH3	-0.0326	2	Number of antenatal visits before 24 weeks	0.337	2	SFH3	0.0180
3	DBP3	0.0273	3	DBP3	-0.0278	3	SFH3	-0.0544	3	DBP3	-0.0272
4	Parity	-0.0049	4	Maternal height	-0.0365	4	Maternal weight before pregnancy	-0.0276	4	Maternal height	0.0312
5	Maternal height	-0.0044	5	Parity	0.0152	5	MAC3	-0.0489	5	MAC3	0.0226
6	Gestational weeks at 1st follow-up	-0.0043	6	MAC3	0.0043	6	Length of a menstrual cycle	0.1569	6	Maternal age	0.0045
7	MAC3	0.0169	7	Maternal weight before pregnancy	-0.0094	7	Maternal age	0.0962	7	Gestational weeks at 1st follow-up	0.0179
8	Maternal age	0.0089	8	Gestational weeks at 1st follow-up	-0.0086	8	Gestational weeks at 1st follow-up	-0.0188	8	SBP3	-0.0094
9	Hemoglobin at register	0.0069	9	Hemoglobin at register	-0.0044	9	Education	0.0408	9	Hemoglobin at register	0.0011
10	SBP3	0.0081	10	Maternal age	0.0036	10	WEIGHT3	-0.0074	10	Length of a menstrual cycle	0.0011
11	Menarche	0.0019	11	SBP3	-0.0025	11	DBP3	-0.0199	11	Education	0
12	Length of a menstrual cycle	0.002	12	Length of a menstrual period	-0.0096	12	SBP3	-0.0139	12	Ethnicity	-0.0001
13	Education	-0.0001	13	Length of a menstrual cycle	-0.0032	13	Length of a menstrual period,	-0.0354	13	Occupation	0
14	Occupation	0	14	Education	0	14	Hemoglobin at register	0.0023			
			15	Ethnicity	0	15	Gynecological surgery history	-0.0453			
			16	Gynecological surgery history	-0.0001	16	Occupation	0.0087			
			17	Occupation	0	17	Ethnicity	-0.0025			
						18	Family disease history	-0.0001			

Supplementary Table S13 The comparison of delivery characteristics between the included pregnancies and excluded pregnancies.

Characteristic	Excluded pregnancies (*n* =124,949)	Included pregnancies (*n* = 225,523)	*P* value
Maternal age at delivery	27 (24, 31)	26 (23, 30)	<0.001
The level of hospitals			<0.001
Provincial or city level hospitals	7467 (6.0)	10173 (4.5)	
county level hospitals	103196 (82.6)	154611 (68.6)	
township level hospitals	7191 (5.8)	9787 (4.3)	
Maternal and Child Health hospitals	7076 (5.7)	50893 (22.6)	
Others	19 (<0.05)	59 (<0.05)	
Maternal delivery methods			<0.001
Vaginal delivery	78886 (63.3)	144471 (64.4)	
cesarean delivery	44751 (35.9)	78012 (34.8)	
Forceps delivery	639 (0.5)	1204 (0.5)	
fetal head traction	86 (0.1)	162 (0.1)	
Assisted breech delivery	122 (0.1)	149 (0.1)	
others	137 (0.1)	196 (0.1)	
Gestational weeks at delivery	39 (38, 40)	39 (38,40)	<0.001
Preterm birth status			<0.001
Term birth	117880 (94.3)	215818 (95.7)	
Preterm birth	7069 (5.7)	9705 (4.3)	
Very preterm birth status			
Birth with gestational weeks ⩾ 30	124370 (99.5)	225010 (99.8)	<0.001
Very preterm birth	579 (0.5)	513 (0.2)	
Extreme preterm birth status			<0.001
Birth with gestational weeks ⩾ 28	124696 (99.8)	225292 (99.9)	
Extreme preterm birth	253 (0.2)	231 (0.1)	
Neonatal Sex			
Girl	57390 (45.9)	104049 (46.1)	
Boy	67537 (54.1)	121454 (53.9)	
Neonatal length (cm)	50 (50, 50)	50 (50, 50)	<0.001
Neonatal weight (g)	3300 (3000, 3580)	3300 (3050, 3600)	<0.001

LBW status			<0.001
Non-LBW	119950 (96)	219153 (97.2)	
LBW	4999 (4.0)	6370 (2.8)	
VLBW status			<0.001
Non-VLBW	124183 (99.4)	224791 (99.7)	
VLBW	766 (0.6)	732 (0.3)	
ELBW status			<0.001
Non-ELBW	124707 (99.8)	225255 (99.9)	
ELBW	242 (0.2)	268 (0.1)	
APGAR1	10 (9, 10)	10 (10, 10)	<0.001
APGAR5	10 (10, 10)	10 (10, 10)	<0.001
Fetal Death			0.093
Alive	124283 (99.5)	224395 (99.5)	
Neonatal Death	32 (<0.05)	36 (<0.05)	
Stillbirth	634 (0.5)	1092 (0.5)	
Fetal distress occurs			<0.001
No	123856 (99.1)	221400 (98.2)	
Yes	1093 (0.9)	4123 (1.8)	
Premature rupture of fetal membrances occurs			<0.001
No	122143 (97.8)	214801 (95.2)	
Yes	2806 (2.2)	10722 (4.8)	
Cord prolapse occurs			0.041
No	124936 (100)	225477 (100)	
Yes	13 (<0.05)	46 (<0.05)	
Placenta previa occurs			<0.001
No	124822 (99.9)	225105 (99.8)	
Yes	127 (0.1)	418 (0.2)	
Pregnancy-induced hypertension syndrome occurs			<0.001
No	124686 (99.8)	224820 (99.7)	
Yes	263 (0.2)	703 (0.3)	
Eclampsia occurs			<0.001

No	124833 (99.9)	225156 (99.8)	
Yes	116 (0.1)	367 (0.2)	
Abruptio placentae occurs			<0.001
No	124779 (99.9)	225102 (99.8)	
Yes	170 (0.1)	421 (0.2)	
Uterine rupture			0.879
No	124942 (100)	225508 (100)	
Yes	7 (<0.05)	15(<0.05)	

Data were represented as median (Quartile 1, Quartile 3) for quantitative variables not following normal distribution, and *n* (%) for qualitative variables. For inter-group comparison analysis, Mann-Whitney *U* test was used for data not following normal distribution or unequal variance, and chi-square test was used for qualitative variables.
